# Analysis of Antibody Markers as Immune Correlates of Risk of Severe COVID-19 in the PREVENT-19 Efficacy Trial of the NVX-CoV2373 Recombinant Protein Vaccine

**DOI:** 10.1093/cid/ciaf558

**Published:** 2025-10-25

**Authors:** Youyi Fong, Yunda Huang, Ying Huang, Wayne Woo, Alice McGarry, Germán Áñez, Lisa M Dunkle, Iksung Cho, Christopher R Houchens, Karen Martins, Lakshmi Jayashankar, Flora Castellino, Christos J Petropoulos, Andrew Leith, Deanne Haugaard, William Webb, Yiwen Lu, Chenchen Yu, Lindsay N Carpp, April K Randhawa, Michele P Andrasik, James G Kublin, Julia Hutter, Maryam Keshtkar-Jahromi, Tatiana H Beresnev, Carina A Rodriguez, Milagritos Tapia, Christine B Turley, Carmen D Zorrilla, Stuart H Cohen, Susan E Kline, Elizabeth Barranco, Lawrence Corey, Kathleen M Neuzil, Dean Follmann, Julie A Ake, Cynthia L Gay, Karen L Kotloff, Thomas Jones, Richard A Koup, Ruben O Donis, Peter B Gilbert

**Affiliations:** Vaccine and Infectious Disease Division, Fred Hutchinson Cancer Center, Seattle, Washington, USA; Public Health Sciences Division, Fred Hutchinson Cancer Center, Seattle, Washington, USA; Department of Biostatistics, University of Washington, Seattle, Washington, USA; Vaccine and Infectious Disease Division, Fred Hutchinson Cancer Center, Seattle, Washington, USA; Department of Global Health, University of Washington, Seattle, Washington, USA; Vaccine and Infectious Disease Division, Fred Hutchinson Cancer Center, Seattle, Washington, USA; Public Health Sciences Division, Fred Hutchinson Cancer Center, Seattle, Washington, USA; Department of Biostatistics, University of Washington, Seattle, Washington, USA; Department of Biostatistics, Novavax, Inc., Gaithersburg, Maryland, USA; Department of Biostatistics, Novavax, Inc., Gaithersburg, Maryland, USA; Department of Biostatistics, Novavax, Inc., Gaithersburg, Maryland, USA; Department of Biostatistics, Novavax, Inc., Gaithersburg, Maryland, USA; Department of Biostatistics, Novavax, Inc., Gaithersburg, Maryland, USA; Biomedical Advanced Research and Development Authority, Administration for Strategic Preparedness and Response, U.S. Department of Health and Human Services, Washington, DC, USA; Biomedical Advanced Research and Development Authority, Administration for Strategic Preparedness and Response, U.S. Department of Health and Human Services, Washington, DC, USA; Biomedical Advanced Research and Development Authority, Administration for Strategic Preparedness and Response, U.S. Department of Health and Human Services, Washington, DC, USA; Biomedical Advanced Research and Development Authority, Administration for Strategic Preparedness and Response, U.S. Department of Health and Human Services, Washington, DC, USA; LabCorp-Monogram Biosciences, South San Francisco, California, USA; Nexelis, Seattle, Washington, USA; Nexelis, Seattle, Washington, USA; Nexelis, Seattle, Washington, USA; Vaccine and Infectious Disease Division, Fred Hutchinson Cancer Center, Seattle, Washington, USA; Vaccine and Infectious Disease Division, Fred Hutchinson Cancer Center, Seattle, Washington, USA; Vaccine and Infectious Disease Division, Fred Hutchinson Cancer Center, Seattle, Washington, USA; Vaccine and Infectious Disease Division, Fred Hutchinson Cancer Center, Seattle, Washington, USA; Vaccine and Infectious Disease Division, Fred Hutchinson Cancer Center, Seattle, Washington, USA; Vaccine and Infectious Disease Division, Fred Hutchinson Cancer Center, Seattle, Washington, USA; Division of AIDS, National Institute of Allergy and Infectious Diseases, NIH, Rockville, Maryland, USA; Division of Microbiology and Infectious Diseases, National Institute of Allergy and Infectious Diseases, NIH, Rockville, Maryland, USA; Division of Microbiology and Infectious Diseases, National Institute of Allergy and Infectious Diseases, NIH, Rockville, Maryland, USA; Division of Pediatric Infectious Diseases, University of South Florida, Morsani College of Medicine, Tampa, Florida, USA; Center for Vaccine Development and Global Health, University of Maryland School of Medicine, Baltimore, Maryland, USA; Atrium Health Wake Forest School of Medicine, Charlotte, North Carolina, USA; Obstetrics and Gynecology Department, School of Medicine, University of Puerto Rico, Medical Sciences Campus, San Juan, Puerto Rico; Department of Internal Medicine, Division of Infectious Diseases, Sacramento, University of California Davis Medical Center, California, USA; Department of Medicine, Division of Infectious Diseases and International Medicine, University of Minnesota Medical School, Minneapolis, Minnesota, USA; Department of Internal Medicine, Ponce Health Sciences University, Ponce, Puerto Rico; Vaccine and Infectious Disease Division, Fred Hutchinson Cancer Center, Seattle, Washington, USA; Department of Laboratory Medicine and Pathology, University of Washington, Seattle, Washington, USA; Center for Vaccine Development and Global Health, University of Maryland School of Medicine, Baltimore, Maryland, USA; Biostatistics Research Branch, National Institute of Allergy and Infectious Diseases, National Institutes of Health, Bethesda, Maryland, USA; U.S. Military HIV Research Program, Center for Infectious Disease Research, Walter Reed Army Institute of Research, Silver Spring, Maryland, USA; Division of Infectious Diseases, University of North Carolina School of Medicine, Chapel Hill, North Carolina, USA; Center for Vaccine Development and Global Health, University of Maryland School of Medicine, Baltimore, Maryland, USA; Biomedical Advanced Research and Development Authority, Administration for Strategic Preparedness and Response, U.S. Department of Health and Human Services, Washington, DC, USA; Vaccine Research Center, National Institute of Allergy and Infectious Diseases, National Institutes of Health, Bethesda, Maryland, USA; Biomedical Advanced Research and Development Authority, Administration for Strategic Preparedness and Response, U.S. Department of Health and Human Services, Washington, DC, USA; Vaccine and Infectious Disease Division, Fred Hutchinson Cancer Center, Seattle, Washington, USA; Public Health Sciences Division, Fred Hutchinson Cancer Center, Seattle, Washington, USA; Department of Biostatistics, University of Washington, Seattle, Washington, USA

**Keywords:** case-cohort sampling, COVID-19 vaccine efficacy trial, exposure-proximal immune correlates analysis, neutralizing antibodies, severe COVID-19

## Abstract

**Background:**

We previously showed that ancestral-specific anti-Spike binding IgG concentration and 50% inhibitory dilution neutralizing antibody titer (nAb-ID50) measured at 2 weeks postdose 2 (∼peak) were inverse correlates of risk (CoRs) of COVID-19 over 2 months post ∼peak in the PREVENT-19 trial of the NVX-CoV2373 vaccine; there were not sufficient data to assess CoRs of severe COVID-19.

**Methods:**

Here, we assessed, in the same vaccinated cohort, Delta- and ancestral-specific Spike IgG and nAb-ID50 at ∼peak and over time as CoRs of severe COVID-19 and of Delta COVID-19 over 3.5–10 months post ∼peak (287 breakthrough Delta cases, including 8 severe; 446 noncases).

**Results:**

Peak antibody levels were much higher for noncases versus severe cases (all inferred Delta), with nAb-ID50 Delta geometric mean 209.5 arbitrary units (AU)/mL (95% CI: 176.1, 249.1) versus 9.6 AU/mL (95% CI: 2.4, 38.6), respectively. Frequency of detectable nAb-ID50 titer was 98.3% (97.2, 99.0) for noncases versus 62.5% (22.3, 93.9) for severe cases. All markers were inverse CoRs of severe COVID-19, with a ∼peak hazard ratio (HR) of 0.13 (95% CI: .03, .57) per 10-fold nAb-ID50 Delta increase. Severe COVID-19 risk through 305 days postday 35 was 0.0338 (0.0043, 0.206) at the nAb-ID50 Delta 2.5th percentile (8.4 AU/mL), and 0.002 (0.0000, 0.0108) and 0.0002 (0.0000, 0.0035) at the 50th and 95th percentiles (210, 2522 AU/mL).

**Conclusions:**

Postvaccination NVX-CoV2373 antibody levels are stronger predictors of severe COVID-19 than any-severity Delta COVID-19. Low antibody responses indicate vulnerability to severe COVID-19.

## Teaser

Neutralizing and binding antibodies elicited by the NVX-CoV2373 vaccine are stronger predictors of severe COVID-19 than of any-severity Delta COVID-19. Low antibody responses indicate high vulnerability to severe COVID-19.

A two-dose regimen of the NVX-CoV2373 (Novavax) recombinant Spike, adjuvanted nanoparticle vaccine was safe [1–3] and effective against RT-PCR–confirmed symptomatic COVID-19 (hereafter, “COVID-19”) in two randomized, blinded, placebo-controlled phase 3 trials [4–7]. This work focuses on one of these trials, PREVENT-19 (NCT04611802), conducted in the United States (US) and Mexico [5]. Participants (n = 29 949) were randomized 2:1 to NVX-CoV2373 or placebo; following demonstration of vaccine efficacy (VE) and safety, blinded crossover was implemented. In the final analysis of the placebo-controlled phase, estimated VE against COVID-19 from 7 days postdose 2 through ∼3 months in per-protocol participants who were seronegative for anti-SARS-CoV-2 nucleoprotein at baseline (had either a negative result or missing data) and who had a SARS-CoV-2 RNA RT-PCR-negative nasal swab at baseline (had either a negative result or missing data) (hereafter, “baseline SARS-CoV-2–negative”) was 90.4% [95% confidence interval (CI): 82.9%, 94.6%; *P* < .001] [5].

In our “stage 1” immune correlates analysis, we reported that IgG binding antibody (bAb) concentration against vaccine-strain (D614) Spike, IgG bAb concentration against D614 RBD, and neutralizing antibody (nAb) titer against D614G Spike-pseudotyped virus, measured in serum samples from the Day 35 visit (2 weeks postdose two, “D35”), each were correlates of risk (CoR) of COVID-19 and associated with VE against COVID-19 in US per-protocol baseline SARS-CoV-2–negative participants [8]. None of the 12 COVID-19 endpoints were severe, precluding stage 1 correlates analysis of severe COVID-19.

Here, we analyze data after the previous data cutoff until booster dose administration (“stage 2”), coinciding with the Delta wave. Estimated counterfactual placebo-controlled VE against Delta COVID-19 was 77% [95% CI: 44%, 90%] at 180 days postdose 2 (novel statistical methods were used to estimate VE given that follow-up extended postcrossover, ie absent a randomized placebo arm for comparison) [9]. As the follow-up period extended from approximately 3.5 through 10 months post-D35, ie, >6 months farther past D35 than our previous correlates analysis, sufficient severe cases (11, including 8 with D35 Ancestral and Delta antibody marker data) occurred to assess CoRs of severe COVID-19.

The first two objectives of this work were to assess D35 Delta-specific and ancestral-specific bAb and nAb markers as (1) CoRs of severe COVID-19 and (2) CoRs of Delta COVID-19. Our third objective accounted for antibody waning: (3) to assess time-varying Delta-specific and ancestral-specific bAb and nAb levels as correlates of instantaneous risk of Delta COVID-19. This “exposure-proximal correlates” analysis uses a hypothetical scenario where the studied antibody marker was measured daily from serum samples collected over follow-up, and assesses how the current-day value of the measured marker correlates with the likelihood of Delta COVID-19 occurring the next day.

## METHODS

### Analysis Cohort

These stage 2 correlates analyses restricted to per-protocol baseline SARS-CoV-2–negative (definitions as in [5]) original vaccine arm participants at US study sites with no evidence of SARS-CoV-2 infection through 108 days post-D35 (Supplementary Figures 1 and 2A).

### Severe and Delta COVID-19 Endpoints

Severe COVID-19 endpoints were as in [5]; Table 1 lists qualifying symptoms. Severe COVID-19 endpoints occurred after the previous data cut [8] (19 April 2021) and after 108 days post-D35 through to 10 December 2021 (Supplementary Figure 2B). Delta COVID-19 endpoints were adjudicated COVID-19 (the COVID-19 primary endpoint in [5], symptomatic RT-PCR–positive COVID-19) occurring in the above time frame, determined to be caused by the Delta variant via nasal swab SARS-CoV-2 genomic sequencing [5] or imputation. If the diagnosis date was on or later than 108 days post-D35 and preceded 10 December 2021, it was assigned Delta. All COVID-19 endpoints with missing lineage assigned Delta occurred after 1 June 2021. Study participants remained blinded during the follow-up considered in this analysis. Median follow-up was 273 (range, 108 to 312) days post-D35. In addition to censoring by follow-up loss, administrative censoring occurred on 10 December 2021.

**Table 1.** List of Symptoms Defining the Severe COVID-19 Endpoint

Symptoms Defining The Severe COVID-19 Endpoint^a,b^
Tachypnea (≥30 breaths per minute at rest)
Resting heart rate ≥125 beats/min
Oxygen saturation: ≤93% on room air or partial pressure of oxygen in the alveolus/fraction of inspired oxygen <300 mmHg
High flow oxygen therapy or noninvasive ventilation/noninvasive positive; noninvasive positive pressure ventilation
Mechanical ventilation or extracorporeal membrane oxygenation
One or more major organ system dysfunction or failure to be defined by diagnostic testing/clinical syndrome/interventions, including any of the following:
Acute respiratory failure, including acute respiratory distress syndrome
Acute renal failure
Acute hepatic failure
Acute right or left heart failure
Septic or cardiogenic shock
Acute stroke (ischemic or hemorrhagic)
Acute thrombotic event: acute myocardial infarction, deep vein thrombosis, pulmonary embolism
Requirement for: vasopressors, systemic corticosteroids, or hemodialysis
Admission to an intensive care unit
Death

As in Dunkle et al [5], the severe COVID-19 endpoint was defined as a first episode of RT-PCR positive COVID-19 with one or more of the symptoms below.

^a^Full details are in Supplementary section 8.4 of the statistical analysis plan.

^b^All participants with a single vital sign abnormality classifying them as severe must also have met the previously defined criteria for mild COVID-19 [5].

### Immunogenicity Subcohort and Case-Cohort Set

Assessment of D35 Delta and ancestral bAb and nAb markers as CoRs of severe COVID-19 and of Delta COVID-19 used a stratified case-cohort sampling design (Supplementary Figure 1). The subcohort for D35 antibody marker measurement was a subset of the stage 1 immunogenicity subcohort [8] originally assigned vaccine (“original vaccine arm”), prioritizing those with D35 ancestral antibody marker data. Sampled Delta cases were a subset of original vaccine arm participants experiencing the Delta COVID-19 endpoint (Supplementary Figures 1 and 2A). “Ancestral” is the vaccine strain (hereafter “D614”) for bAb markers and the vaccine strain harboring the D614G mutation (“D614G”) for nAb markers. Noncases were per-protocol baseline SARS-CoV-2–negative original vaccine arm participants randomly sampled from the stage 1 immunogenicity subcohort [8] with available D35 ancestral and D35 Delta antibody data with no evidence of SARS-CoV-2 infection (ie, never tested RT-PCR positive) by 10 December 2021. Supplementary Table 1 enumerates the subcohort across baseline sampling strata of the Delta cases and noncases included in the analyses.

D35 Delta and D35 ancestral nAb and bAb levels were measured from 287 of the 467 original vaccine arm participant breakthrough Delta cases, and from 8 of the 11 original vaccine arm participant breakthrough severe cases (follow-up period ∼3.5 through 10 months post-D35). Only one participant acquiring the severe disease endpoint died, precluding CoR analyses of mortality. D35 Delta and D35 ancestral nAb and bAb levels were also measured from 456 participants sampled from the stage 1 immunogenicity subcohort, comprising 446 noncases and 10 Delta cases (Supplementary Figures 1 and 2A).

### nAb Assay

nAb titers were quantified in a validated assay [10] utilizing lentiviral particles pseudotyped with D614G or Delta B.1.617.2 Spike. Titers are serum sample inhibitory dilutions at which relative luminescence units were reduced by 50% (ID50) versus control wells after subtracting background relative luminescence units. Both nAb-ID50 D614G and nAb-ID50 Delta titers in AU/mL, measured by the same lab, were multiplied by the constant conversion factor 0.0653, which converts the nAb-ID50 D614G titers in AU/mL to International Units/mL (IU50/mL) [8, 11]. nAb-ID50 titers against Delta remain in AU/mL because international units do not exist for this Delta variant readout. Supplementary Table 2 provides assay limits.

### Binding Antibody Assay

IgG bAb concentrations against D614 Spike and Delta AY.4.2 Spike (“Delta Spike”) were measured using the MSD V-PLEX SARS-CoV-2 Panel 23 Kit. Assay readouts were IgG concentration expressed in AU/mL. Both Spike IgG D614 and Spike IgG Delta bAb concentrations in AU/mL, measured by the same lab, were multiplied by the constant conversion factor 0.009, which converts the Spike IgG D614 bAb concentrations in AU/mL to Binding Antibody Units/mL (BAU/mL) [8, 11]. Spike IgG Delta concentrations remain in AU/mL because international units do not exist for this Delta variant readout. Supplementary Table 2 provides assay limits.

### Statistical Analyses

All immune correlates analyses were prespecified. The statistical analysis plan is provided in the Supplementary Material.

### Covariate Adjustment

D35 CoR analyses of severe COVID-19 adjusted for age in years, while analyses for Delta COVID-19 adjusted for age (≥65 vs <65).

### Approximate Peak (D35) Immune CoR Analysis

For each D35 marker, the covariate-adjusted HR of severe COVID-19 or of Delta COVID-19 was estimated using inverse probability sampling weighted Cox regression models with 95% CIs and Wald-based *P*-values. These Cox model fits were also used to estimate marker-conditional covariate-marginalized cumulative incidence of severe COVID-19 or Delta COVID-19 from 108 through 305 days post-D35. 95% CIs were computed using percentile bootstrap. Cox models were fit using the survey [12] R [13] package.

### Hypothesis Testing

For hypothesis tests for D35 marker CoRs, Westfall–Young multiplicity adjustment [14] was applied to obtain false-discovery rate adjusted *P*-values and family wise error rate adjusted *P*-values. Permutation-based multiple-testing adjustment was performed over the quantitative marker and tertile CoR analyses. All *P*-values were two-sided.

### Exposure-proximal CoR Analysis

For estimating exposure-proximal immune correlates, longitudinal case-control antibody marker data (Supplementary Figure 9) and a regression calibration-based approach (Supplementary Methods) were used [15].

## RESULTS

### Participant Demographics

In the immunogenicity subcohort (N = 560), 47.9% were ≥65 years old, 48.6% were considered at risk of severe COVID-19, and 47.1% were female (Supplementary Table 3). Additionally, 40.2% were minority (other than White Non-Hispanic), with 17.5% Black or African American, 21.2% Hispanic or Latino, 7.3% Asian, and 2.5% American Indian or Alaska Native.

### D35 Marker Intercorrelations

D35 nAb-ID50 D614G titers were highly correlated with D35 nAb-ID50 Delta titers (Spearman rank correlation r = 0.90), as were D35 Spike IgG D614 concentrations and D35 Spike IgG Delta concentrations (r = 0.96) (Supplementary Figure 3B).

### Antibody Response Frequencies and Levels by COVID-19 Outcome Status

Table 2 shows D35 Delta antibody marker response frequencies, GM concentrations, and GM titers in severe cases, Delta cases, and noncases. In the main text, we focus on reporting the Delta nAb-ID50 results, given that Delta Spike IgG and ancestral-specific results were similar. The frequency of detectable Delta nAb-ID50 was lowest in severe cases, intermediate in Delta cases, and highest in noncases: 62.5%, 93.9%, and 98.3%, respectively. Similarly, Delta nAb-ID50 titers were lowest in severe cases, intermediate in Delta cases, and highest in noncases: GM titers 9.6, 125.0, and 209.5 AU/mL, respectively. GM titer ratios were 0.05 for severe cases to noncases and 0.60 for Delta cases to noncases.

**Table 2.** D35 Delta and Ancestral Antibody Marker Positive Response Frequencies and Geometric Means by COVID-19 Outcome Status

D35 Marker	Severe COVID-19 Cases			Delta COVID-19 Cases			Noncases			Comparison: Severe- COVID-19 Cases to Noncases		Comparison: Delta COVID-19 Cases to Noncases	
	N	Pos Resp Freq	GM Titer or Concentration (95% CI)	N	Pos Resp Freq	GM Titer or Concentration (95% CI)	N	Pos Resp Freq	GM Titer or Concentration (95% CI)	Response Freq Difference (Cases—Noncases)	Ratio of GM Titer or Concentration (Cases/Noncases)	Response Freq Difference (Cases—Noncases)	Ratio of GM Titer or Concentration (Cases/Noncases)
nAb-ID50 Delta (AU/mL)	8	62.5% (22.3%, 90.6%)	9.57 (2.37, 38.58)	287	93.9% (90.5%, 96.1%)	125.0 (101.1, 154.5)	446	98.3% (97.2%, 99.0%)	209.5 (176.1, 249.1)	−0.358 (−0.761, −0.077)	0.05 (0.01, 0.19)	−0.044 (−0.079, −0.019)	0.60 (0.45, 0.78)
Spike IgG Delta (AU/mL)	8	75.0% (29.5%, 95.5%)	32.71 (9.13, 117.2)	287	97.7% (95.2%, 98.9%)	498.7 (420.7, 591.1)	446	99.7% (99.2%, 99.9%)	824.4 (729.9, 931.1)	−0.247 (−0.702, −0.041)	0.04 (0.01, 0.14)	−0.02 (−0.045, −0.007)	0.60 (0.49, 0.75)
nAb-ID50 D614G (IU50/mL)	8	75.0% (29.5%, 95.5%)	26.36 (5.67, 122.5)	287	97.3% (94.7%, 98.7%)	400.8 (329.3, 487.8)	446	99.7% (99.3%, 99.9%)	626.7 (542.0, 724.7)	−0.247 (−0.702, −0.042)	0.04 (0.01, 0.20)	−0.024 (−0.05, −0.01)	0.64 (0.50, 0.82)
Spike IgG D614 (BAU/mL)	8	75.0% (29.5%, 95.5%)	68.74 (14.90, 317.2)	287	98.0% (95.7%, 99.1%)	1182.7 (999.5, 1400)	446	99.8% (99.4%, 100.0%)	1792 (1599, 2008)	−0.248 (−0.703, −0.043)	0.04 (0.01, 0.18)	−0.018 (−0.042, −0.006)	0.66 (0.54, 0.81)

Analysis based on US per-protocol baseline SARS-CoV-2–negative original vaccine arm participants with no evidence of SARS-CoV-2 infection through 108 d post-D35. Median (interquartile range) days from vaccination to D35 was 37 (3.25) for severe COVID-19 cases, 37 (5) for delta COVID-19 cases, and 38 (6) for noncases.

Severe COVID-19 cases are per-protocol baseline SARS-CoV-2–negative original vaccine arm recipients with the severe COVID-19 endpoint diagnosed starting both after 19 April 2021 and 108 d after the Day 35 study visit through to 10 December 2021. Delta COVID-19 cases are per-protocol baseline SARS-CoV-2–negative original vaccine arm recipients with the symptomatic infection COVID-19 primary endpoint diagnosed starting both after 19 April 2021 and 108 d after the Day 35 study visit through to 10 December 2021. Noncases are per-protocol baseline SARS-CoV-2–negative original vaccine arm participants randomly sampled from the Fong et al immunogenicity subcohort [8] with available D35 ancestral and D35 Delta antibody data with no evidence of SARS-CoV-2 infection (ie, never tested RT-PCR positive) by 10 December 2021. Numbers of severe and of Delta COVID-19 cases are smaller than those in Table 3 because only cases with available D35 antibody data (nAb-ID50 Delta, Spike IgG Delta, nAb-ID50 D614G, and Spike IgG D614) are included in this table.

Positive (quantifiable) nAb-ID50 response at D35 was defined as D35 nAb-ID50 value ≥ to the antigen-specific LOD; otherwise the response is not detectable. Participants with a positive (quantifiable) Spike IgG response at D35 was defined as D35 Spike IgG value ≥ antigen-specific LLOQ; otherwise the response is not detectable. LOD = 2.612 AU/mL for nAb-ID50 Delta and for nAb-ID50 D614G; LLOQ = 5.5278 AU/mL for Spike IgG Delta and 5.0742 AU/mL for Spike IgG D614.

AU, arbitrary units; BAU, binding antibody units; CI, confidence interval; GM, geometric mean; IU, international units; LLOQ, lower limit of quantitation; LOD, limit of detection; nAb-ID50, 50% inhibitory dilution neutralizing antibody; Pos Resp Freq, positive response frequency.

**Figure 1.**
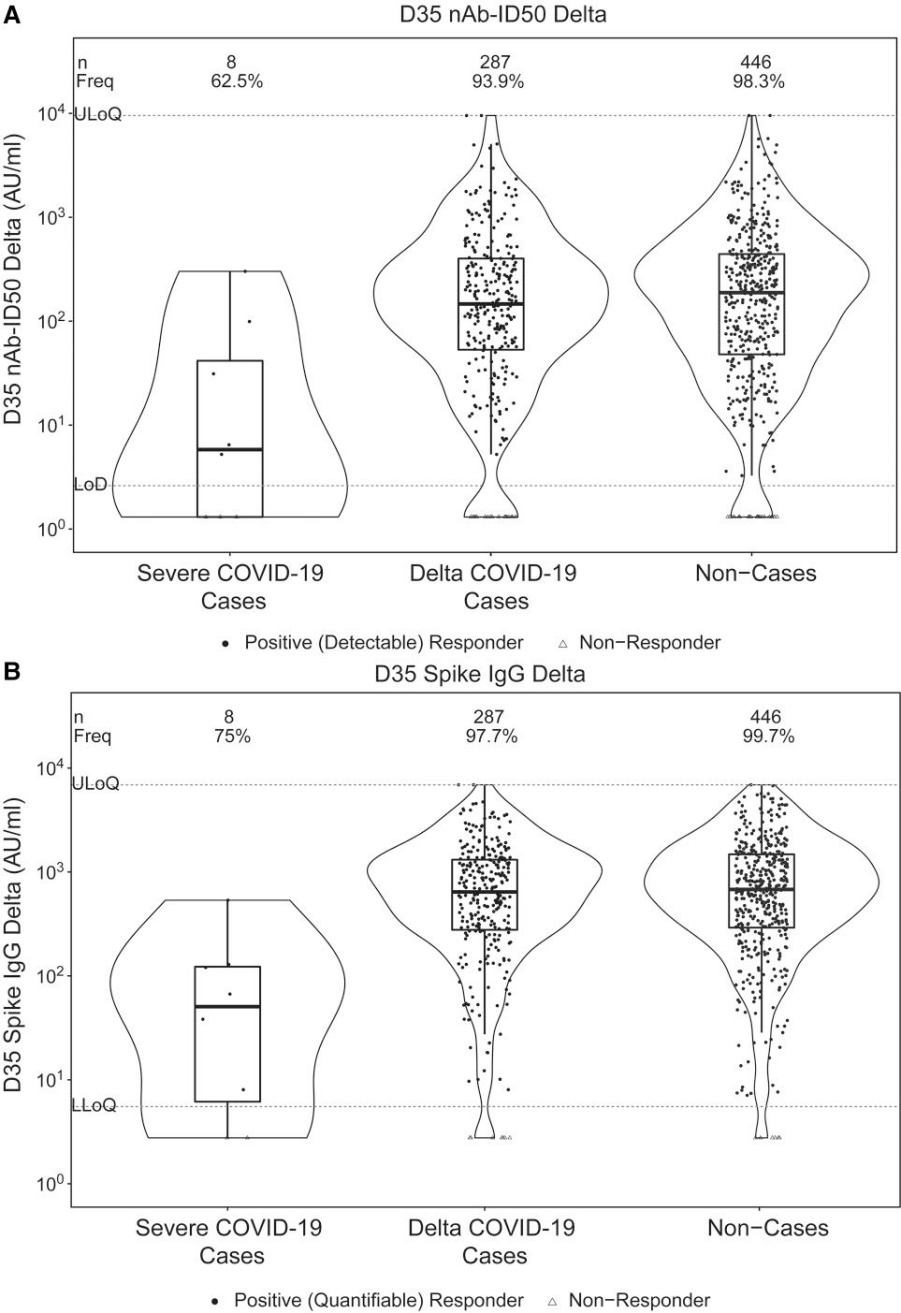
Violin plots showing D35 levels of (A) 50% inhibitory dilution Delta-neutralizing antibody titer (nAb-ID50 Delta) in AU/mL; (B) Anti-Delta Spike IgG concentration (Spike IgG Delta) in AU/mL, shown separately for severe COVID-19 cases, Delta COVID-19 cases, and noncases. Analysis based on US per-protocol baseline SARS-CoV-2–negative original vaccine arm participants with no evidence of SARS-CoV-2 infection through 108 d post-D35. The violin plots contain interior box plots with upper and lower horizontal edges the 25th and 75th percentiles of antibody level and middle line the 50th percentile, and vertical bars the distance from the 25th (or 75th) percentile of antibody level and the minimum (or maximum) antibody level within the 25th (or 75th) percentile of antibody level minus (or plus) 1.5 times the interquartile range. At both sides of the box, a rotated probability density curve estimated by a kernel density estimator with a default Gaussian kernel is plotted. Numbers of participants (*n*) in each group with antibody data are reported at the top of the plots. Filled circles are positive (detectable) nAb-ID50 Delta or positive (quantifiable) Spike IgG Delta responses; open triangles are nonresponders. Frequencies of participants with positive (detectable) nAb-ID50 Delta or positive (quantifiable) Spike IgG Delta responses were computed with inverse probability of sampling weighting and are also reported at the top of the plots as “Freq.” Positive (detectable) D35 nAb-ID50 Delta response was defined as D35 nAb-ID50 Delta ≥ LOD (2.612 AU/mL). ULoQ = 9524.53 AU/mL for nAb-ID50 Delta. Positive (quantifiable) D35 Spike IgG Delta was defined as D35 Spike IgG Delta ≥ LLOQ (5.5278 AU/mL). ULoQ = 6900.02 AU/mL for Spike IgG Delta. Cases were in the original vaccine arm and experienced the severe COVID-19 endpoint and/or Delta COVID-19 endpoint, as applicable, after both 19 April 2021 and 108 d post-D35 through to 10 December 2021. Noncases were defined as baseline SARS-CoV-2–negative per-protocol original vaccine arm participants randomly sampled from the Fong et al immunogenicity subcohort with available D35 ancestral and D35 Delta antibody data with no evidence of SARS-CoV-2 infection (ie, never tested RT-PCR positive) through 10 December 2021. AU, arbitrary units; LLOQ, lower limit of quantitation; LOD, limit of detection; ULOQ, upper limit of quantitation.

Figure 1 shows the distributions of D35 Delta marker levels in severe cases, Delta cases, and noncases. Clear distinction is observed between severe cases and noncases, but not between Delta cases and noncases. Strikingly, there were no severe cases above the 66th percentile of D35 nAb-ID50 Delta (417 AU/mL), nor above the 33rd percentile of D35 Spike IgG Delta (562 AU/mL), where percentiles were calculated for the population for which inferences about correlates are drawn using inverse sampling probability weights (Supplementary Figure 5). In contrast, Delta cases occurred even at the highest observed values of D35 nAb-ID50 Delta and D35 Spike IgG Delta (Figure 1).

Supplementary Figures 4 and 6 show similar results for the ancestral markers.

### D35 Delta Antibody Inverse CoRs of Severe COVID-19 and Delta COVID-19

**Figure 2.**
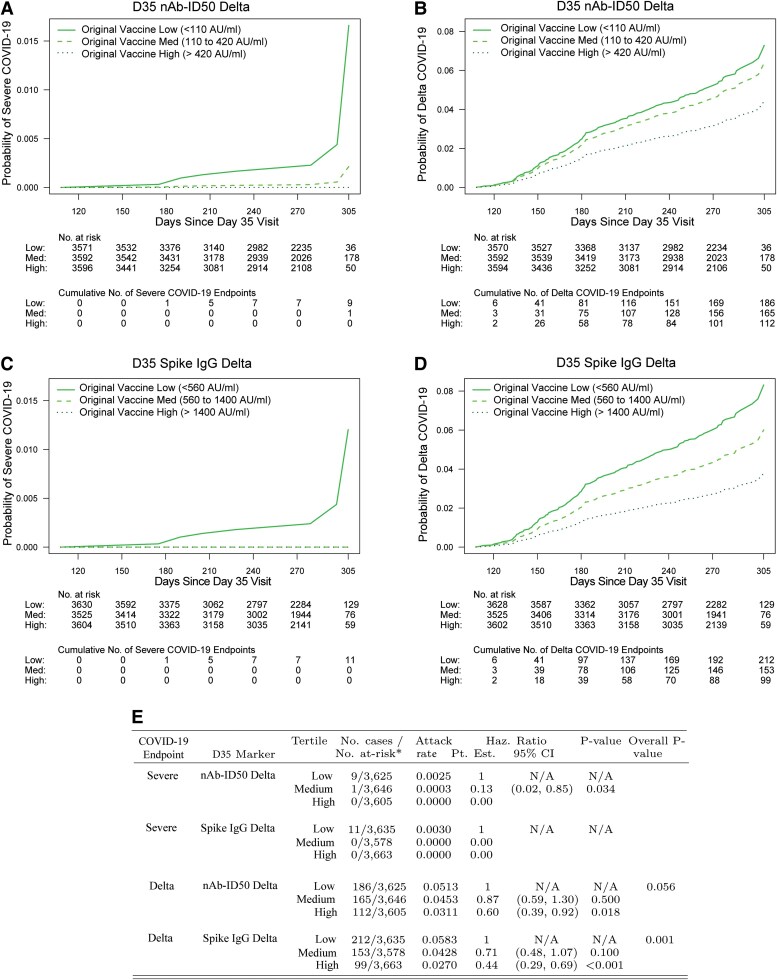
Covariate-marginalized cumulative incidence of (A, C) severe COVID-19 or (B, D) Delta COVID-19 from 108 through 305 d post-D35 by low, medium, and high tertile of D35 Delta antibody marker level: (A, B) nAb-ID50 Delta; (C, D) Spike IgG Delta. Marker levels defining the low, medium, and high tertiles are shown in the upper left of each plot, with cutoff values determined as the 33rd and 67th percentiles of the D35 marker. Percentiles were calculated for the population for which inferences about correlates are drawn using inverse sampling probability weights. (E) Estimated hazard ratios of severe COVID-19 or of Delta COVID-19 for the medium versus low and for the high versus low tertiles of D35 nAb-ID50 Delta or of D35 Spike IgG Delta. For tertiles with zero severe cases (high nAb-ID50 Delta, medium spike IgG Delta, high spike IgG Delta), the Cox modeling cannot provide inferences involving comparison of that tertile to another tertile. The overall *P*-value is from a generalized Wald-test *P*-value of the null hypothesis that the hazard rate is constant across the low, medium, and high tertile groups. Analyses were based on US per-protocol baseline SARS-CoV-2–negative original vaccine arm participants with no evidence of SARS-CoV-2 infection through 108 d post-D35. Analyses for severe COVID-19 adjusted for age in years, while analyses for Delta COVID-19 adjusted for age group (≥65 vs <65). *No. at-risk = estimated number in the population for analysis, ie, baseline negative per-protocol original vaccine arm participants without evidence of SARS-CoV-2 infection through 108 d post-D35. Case counting started 108 d post-D35. The total case counts across tertiles are larger than those in Figure 1 and Table 2 because the cases shown here do not require availability of D35 antibody data. nAb-ID50, 50% inhibitory dilution neutralizing antibody.

The cumulative incidence of severe COVID-19 through ∼10 months postdose 2 decreased with increasing D35 antibody marker tertile for both nAb-ID50 Delta and Spike IgG Delta (Figure 2A and 2C). For nAb-ID50 Delta, there were 9, 1, and 0 breakthrough severe cases in the Low, Medium, and High subgroups, with covariate-adjusted hazard ratio (HR) point estimate (95% CI) of 0.13 (0.02, 0.85) (*P* = .034) for Medium versus Low. Results were similar for Spike IgG Delta, with 11, 0, and 0 breakthrough severe cases in the Low, Medium, and High subgroups. The estimated cumulative incidence of severe COVID-19 through 10 months was 0.017 for the nAb-ID50 Delta Low subgroup, compared to 0.002 for the nAb-ID50 Delta Medium subgroup, highlighting the large magnitude point estimates of association.

The cumulative incidence of Delta COVID-19 likewise decreased with increasing tertile of D35 Delta antibody marker (Figure 2B and 2D). For nAb-ID50 Delta, there were 186, 165, and 112 breakthrough Delta cases in the low, medium, and high subgroups, with covariate-adjusted HR point estimates (95% CI) of 0.87 (0.59, 1.30) (*P* = .50) for medium versus low and 0.60 (0.39, 0.92) (*P* = .018) for high versus low. Results were similar for Spike IgG Delta, with 212, 153, and 99 breakthrough Delta cases in the low, medium, and high subgroups and covariate-adjusted HR point estimates (95% CI) of 0.71 (0.48, 1.07) (*P* = .10) for medium versus low and 0.44 (0.29, 0.69) (*P* < .001) for high versus low. Moreover, for Spike IgG Delta, the HR of Delta COVID-19 differed significantly across the low, medium and high tertiles (overall *P* = .001).

Supplementary Figure 7 shows similar results for the ancestral markers.

The CoR results for the quantitative D35 markers are shown in Table 3. nAb-ID50 Delta and Spike IgG Delta each significantly inversely correlated with severe COVID-19 [estimated HR (95% CI) per 10-fold increase in marker level: 0.13 (0.03, 0.57) (*P* = .007) for nAb-ID50 Delta; 0.11 (0.05, 0.26) (*P* < .001) for Spike IgG Delta]. Each D35 marker also inversely correlated with Delta COVID-19, with an estimated HR of 0.60 (0.47, 0.79) (*P* < .001) for nAb-ID50 Delta and 0.48 (0.35, 0.67) (*P* < .001) for Spike IgG Delta.

**Table 3.** Covariate-adjusted Hazard Ratios of (A) Severe COVID-19 or (B) Delta COVID-19 per 10-fold Increase or per Standard Deviation Increase in Each Delta or Ancestral D35 Antibody Marker

D35 Marker	No. cases^a^/No. at-risk^b^	HR per 10-fold increase		*P*-value	HR per standard deviation increase	
		Pt. Est.	95% CI		Pt. Est.	95% CI
A. Severe COVID-19						
nAb-ID50 Delta (AU/mL)	11/10 879	0.13	.03, .57	0.007	0.19	.06, .64
Spike IgG Delta (AU/mL)	11/10 879	0.11	.05, .26	<0.001	0.24	.14, .42
nAb-ID50 D614G (IU50/mL)	11/10 879	0.13	.04, .39	<0.001	0.22	.10, .51
Spike IgG D614 (BAU/mL)	11/10 879	0.14	.07, .29	<0.001	0.29	.18, .46
B. Delta COVID-19						
nAb-ID50 Delta (AU/mL)	467/10 879	0.60	.47, .79	<0.001	0.67	.54, .82
Spike IgG Delta (AU/mL)	467/10 879	0.48	.35, .67	<0.001	0.63	.51, .77
nAb-ID50 D614G (IU50/mL)	467/10 879	0.58	.43, .77	<0.001	0.67	.54, .83
Spike IgG D614 (BAU/mL)	467/10 879	0.52	.38, .71	<0.001	0.66	.55, .81

Analyses were based on US per-protocol baseline SARS-CoV-2–negative original vaccine arm participants with no evidence of SARS-CoV-2 infection through 108 d post-D35. Analyses for severe COVID-19 adjusted for age in years, while analyses for delta COVID-19 adjusted for age group (≥65 vs <65).

AU/mL, arbitrary units/mL; BAU/mL, binding antibody units/mL; IU50/mL, International Units/mL.

^a^Of the 11 severe COVID-19 cases, 8 had D35 antibody data; of the 467 Delta COVID-19 cases, 287 had D35 antibody data (see Table 2).

^b^No. at-risk = estimated number in the population for analysis, ie per-protocol baseline SARS-CoV-2 negative original vaccine arm participants with no evidence of SARS-CoV-2 infection through 108 d post-D35.

**Figure 3.**
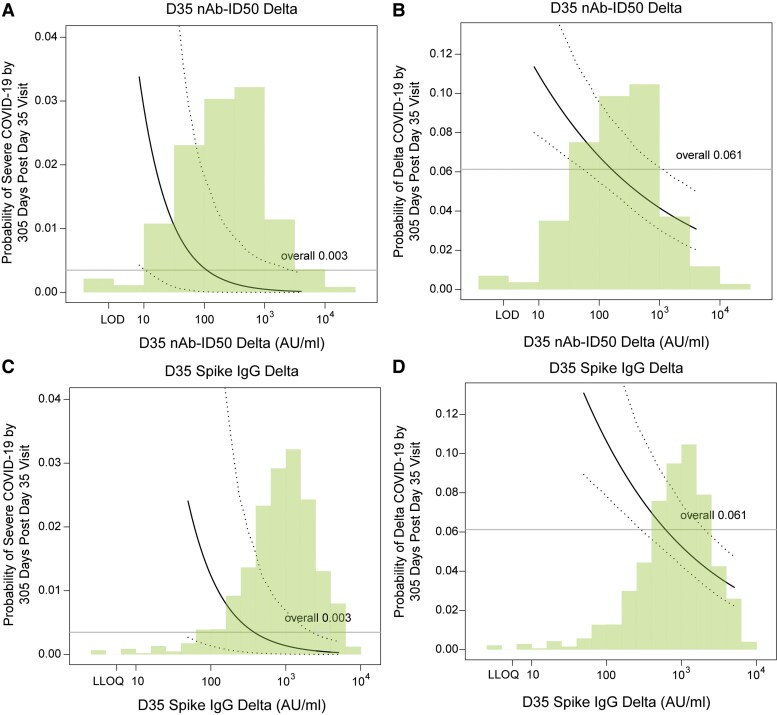
Covariate-marginalized cumulative incidence of severe COVID-19 and of Delta COVID-19 from 108 through 305 d post-D35 by D35 Delta antibody marker level, estimated using a marginalized Cox model. Plots are shown for (A, C) severe COVID-19 or (B, D) Delta COVID-19, by (A, B) nAb-ID50 Delta titer or (C, D) anti-Spike IgG Delta concentration. The dotted black lines indicate bootstrap pointwise 95% CIs. The horizontal gray line is the overall cumulative incidence of severe COVID-19 (or Delta COVID-19, as relevant) from 108 to 305 d post-D35 in the original vaccine arm. Curves are plotted over the antibody marker range from the 2.5th percentile to the 97.5th percentile: 8.36 to 4021 AU/mL for nAb-ID50 Delta, 49.5 to 5062 AU/mL for Spike IgG Delta. Analyses were based on US per-protocol baseline SARS-CoV-2–negative original vaccine arm participants with no evidence of SARS-CoV-2 infection through 108 d post-D35. Analyses for severe COVID-19 adjusted for age in years, while analyses for Delta COVID-19 adjusted for age group (≥65 vs <65).

Figure 3A and 3C show estimates of cumulative incidence of severe COVID-19 through 10 months conditional on quantitative D35 nAb-ID50 Delta and Spike IgG Delta, with adjustment of (marginalization over) the baseline factors. For D35 nAb-ID50 Delta titer at the 10th, 50th, and 90th percentile (28, 210, 1224 AU/mL), the cumulative incidence point estimate (95% CI) was 0.012 (0.001, 0.057), 0.002 (0.000, 0.011), and 0.0004 (0.0000, 0.0046), respectively. For D35 Spike IgG Delta at the 10th, 50th, and 90th percentile (196, 922, 3024 AU/mL), the cumulative incidence was 0.006 (0.001, 0.032), 0.002 (0.000, 0.007), and 0.001 (0.000, 0.003), respectively.

Figures 3B and 3D show estimates of cumulative incidence of Delta COVID-19 through 10 months conditional on quantitative D35 nAb-ID50 Delta and Spike IgG Delta, with adjustment of (marginalization over) the baseline factors. For D35 nAb-ID50 Delta titer at the 10th, 50th, and 90th percentile (28, 210, 1224 AU/mL), the point estimate of cumulative incidence (95% CI) was 0.088 (0.068, 0127), 0.058 (0.047, 0.083), and 0.040 (0.029, 0.061), respectively. For D35 Spike IgG Delta at the 10th, 50th, and 90th percentile (196, 922, 3024 AU/mL), the cumulative incidence was 0.087 (0.067, 0.127), 0.054 (0.044, 0.077), and 0.037 (0.028, 0.055), respectively.

Supplementary Figure 8 shows similar results for the ancestral markers.

### Time-varying Delta Antibody Markers Inversely Correlated With Delta COVID-19

**Figure 4.**
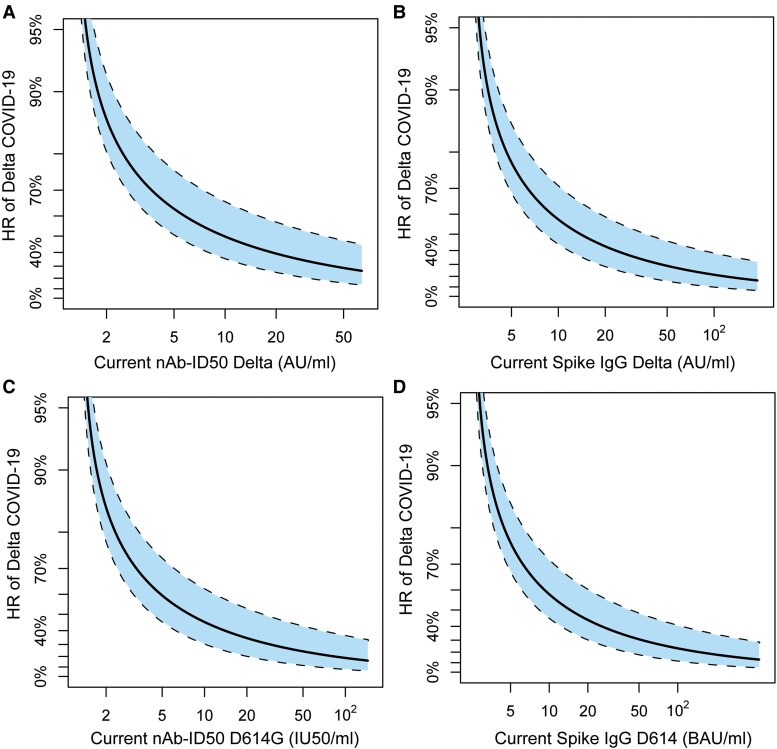
Hazard ratio with reference antibody level LOD/2 (for nAb-ID50) or LLOQ/2 (for Spike IgG) of Delta COVID-19 as a function of current antibody marker level modeled over time: (A) nAb-ID50 Delta titer, (B) anti-Spike IgG Delta concentration, (C) nAb-ID50 D614G titer, or (D) anti-Spike IgG D614 concentration. “Current” refers to the true underlying antibody marker level not subject to technical measurement error, in a hypothetical scenario in which the value was available from serum samples collected every day over the follow-up period (see Supplementary Methods). Analyses were based on US per-protocol baseline SARS-CoV-2–negative original vaccine arm participants with no evidence of SARS-CoV-2 infection through 108 d post-D35. Analyses adjusted for age group (≥65 vs <65). The dashed black lines indicate bootstrap pointwise 95% CIs. Plots range from unquantifiable titer (A, C) or undetectable concentration (B, D) up to the 97.5th percentile in the current value of the marker. AU, arbitrary units; BAU, binding antibody units; IU, international units; LOD, limit of detection; LLOQ, lower limit of quantitation. LOD = 2.612 AU/mL for nAb-ID50 Delta and 2.612 IU50/mL for nAb-ID50 D614G; LLOQ = 5.5278 AU/mL for Spike IgG Delta and 5.0742 BAU/mL for Spike IgG D614.

To account for the decline in antibody levels over time, we performed an exposure-proximal correlates analysis. The HR of Delta COVID-19 decreased as the current nAb-ID50 Delta titer rose across the analyzed range (Figure 4A). Similar results were observed for the current Spike IgG Delta concentration, nAb-ID50 D614G titer, and Spike IgG D614 concentration (Figure 4B–D).

## DISCUSSION

NVX-CoV2373 vaccine-induced binding and neutralizing antibodies were each inverse CoR of severe COVID-19 and of Delta COVID-19 over 3.5–10 months. A major new finding was that severe COVID-19 risk decreased dramatically with D35 nAb titer and with D35 Spike IgG binding antibody concentration measured at 2 weeks postdose 2. We observed a sharp distinction in D35 antibody levels between severe cases versus the study population at large. For example, all severe COVID-19 cases were restricted to the bottom one-third of D35 Spike IgG binding antibody concentrations, and to the bottom two-thirds of D35 nAb titers. Given that SARS-CoV-2-specific CD4+ and CD8+ T cell responses [16–24] and/or non-neutralizing effector functions [25, 26] appear to be important in preventing severe disease, one possibility is that low levels of antibodies associate with low levels of another immune response implicated in protective immunity against severe disease.

Such a strong separation in antibody levels (severe cases vs the study population) was not observed in the other phase 3 vaccine trials that also evaluated immune correlates against severe COVID-19 based on individual-level data. Carpp, Hyrien, and Fong et al [27] assessed immune correlates of severe COVID-19 in the ENSEMBLE trial (pre-Delta wave) of the Ad26.COV2.S vaccine and Janes et al (under revision) assessed immune correlates of severe COVID-19 during the Delta wave in the AZD1222 trial of the ChAdOx1 nCoV-19 vaccine (we are not aware of any relevant data for mRNA COVID-19 vaccines for comparison, due to too few severe COVID-19 cases in the respective trials for correlates of severe disease to be assessed). The geometric mean ∼peak D614G nAb titers [assessed in ENSEMBLE (Ad26.COV2.S) [27], AZD1222 (ChAdOx1 nCoV-1) (Janes et al, under revision), and PREVENT-19 (NVX-CoV2373) (this work) and expressed in the same International Units], were ∼100-fold and 33-fold lower in noncases in ENSEMBLE (postone dose) and AZD1222 (post-two doses), respectively, versus those in PREVENT-19. In contrast, the GM titers of the severe cases did not vary as much (∼6-fold lower in severe cases, for both ENSEMBLE and AZD1222, vs in PREVENT-19). Moreover, the maximum ∼peak D614G nAb titers were <2-fold different across the studies: 185, 227, and 392 IU50/mL for ENSEMBLE, AZD1222, and PREVENT-19, respectively. Therefore, the strong humoral response induced by the NVX-CoV2373 vaccine is a plausible explanation for the observed large separation in antibody levels in severe cases versus the rest of the study population.

A limitation of this analysis is the small number (eight) of severe COVID-19 breakthrough endpoints with D35 antibody data, which limited statistical precision. The observed (very) strong and statistically significant associations should be confirmed in additional studies. Moreover, we could not perform correlates of protection analyses due to the lack of a randomized comparison arm (eg placebo). However, the substantial (approximately 77% by 6 months) estimated counterfactual placebo-controlled VE of NVX-CoV2373 against Delta COVID-19 [9] suggests that the D35 and modeled-over-time antibody markers are not only CoR but also correlates of protection. Another limitation of this analysis, which also applies to most correlate analyses of COVID-19 vaccines, is that it did not include cellular responses. Additionally, IgG binding antibody responses were not analyzed by isotype; such analyses may be of interest given that NVX-CoV2373 recipients were reported to have >10 times higher levels of SARS-CoV-2 specific IgG3 compared to mRNA COVID-19 vaccine recipients [28].

A strength of this study is the large number of participants with evaluable breakthrough Delta COVID-19 endpoints (287), attributable to the fact that some follow-up coincided with the Delta wave, as well that the crossover likely ensured high retention. This large number of Delta endpoints provided high statistical precision for assessing D35 and exposure-proximal Delta COVID-19 correlates, yielding small *P*-values indicating very strong evidence of correlates; the stage 1 correlates analysis showed strong point estimates for D35 inverse correlates of COVID-19 but with wider CIs [8]. Another strength is that correlates of severe COVID-19 could be assessed, yielding strong statistical evidence of real CoR with the *P*-values for all four D35 markers lower than 0.005. The relatively wide CIs support a qualitative conclusion: low D35 antibody levels from either immunoassay are a clear biomarker phenotype of elevated risk of severe COVID-19. These findings may potentially inform future vaccination strategies, booster recommendations, and/or pre-exposure prophylaxis based on antibody levels, particularly in populations at higher risk for severe outcomes, although further research would be needed to determine whether the findings hold after booster vaccinations and against Omicron viruses.

## Supplementary Material

ciaf558_Supplementary_Data

## Supplementary Data

Supplementary materials are available at *Clinical Infectious Diseases* online. Consisting of data provided by the authors to benefit the reader, the posted materials are not copyedited and are the sole responsibility of the authors, so questions or comments should be addressed to the corresponding author.
