# Supplementary material for: Analysis of Antibody Markers as Immune Correlates of Risk of Severe COVID-19 in the PREVENT-19 Efficacy Trial of the NVX-CoV2373 Recombinant Protein Vaccine
Source: Clin Infect Dis. 2025 Oct 25;82(4):e863–74. doi: 10.1093/cid/ciaf558 (PMC12611457; doi:10.1093/cid/ciaf558)
Supplement: ciaf558_Supplementary_Data [file ciaf558_supplementary_data.pdf]

Immune Assays Team

Affiliation	Team Members
Biomedical Advanced Research and Development Authority (BARDA), Washington, DC	Victor Ayala, Oleg Borisov, Flora Castellino, Brett Chromy, Mark Delvecchio, Ruben O. Donis, Tremel Faison, Corey Hoffman, Christopher Houchens, Tom Hu, Pennie Hylton, Lakshmi Jayashankar, Aparna Kolhekar, James Little, Karen Martins, Jeanne Novak, Azhar Ravji, Carol Sabourin, Evan Sturtevant, Kimberly Taylor, Xiaomi Tong, John Treanor, Danielle Turley, Leah Watson, Daniel Wolfe
Boston Consulting Group, Boston, MA	Gian King, Andrew Li, Najaf Shah, Smruthi Suryaprakash, Jue Xiang Wang
Division of AIDS, NIAID, NIH, Bethesda, MD	Patricia D'Souza
Division of MID (Microbiology and Infectious Diseases), NIAID, NIH, Bethesda, MD	Janie Russell
Duke University, Durham, NC	David Beaumont, Kendall Bradley, Jiayu Chen, Xiaoju Daniell, Thomas Denny, Elizabeth Domin, Amanda Eaton, Kelsey Engel, Wenhong Feng, Juanfei Gao, Hongmei Gao, Kelli Greene, Sarah Hiles, Leihua Liu, Kristy Long, Kellen Lund, Charlene McDanal, David C. Montefiori, Marcella Sarzotti-Kelsoe, Francesca Suman, Haili Tang, Jin Tong, Olivia Widman
LabCorp-Monogram Biosciences, South San Francisco, CA, USA	Christos J. Petropoulos, Terri Wrin
The Tauri Group, an LMI company - Contract Support for U.S. Department of Defense (DOD) Joint Program Executive Office for Chemical, Biological, Radiological and Nuclear Defense (JPEO-CBRND) Joint Project Manager for Chemical, Biological, Radiological, and Nuclear Medical (JPM CBRN Medical), Fort Detrick, Maryland, USA	Christopher S. Badorrek, Gregory E. Rutkowski
Vaccine Research Center, NIAID, NIH, Bethesda, MD	Obrimpong Amoa-Awua, Manjula Basappa, Robin Carroll, Britta Flach, Suprabhath Gajjala, Nazaire Jean-Baptiste, Richard A. Koup, Bob C. Lin, Adrian McDermott, Christopher Moore, Mursal Naisan, Muhammed Naqvi, Sandeep Narpala, Sarah O'Connell, Clare Whittaker, Weiwei Wu, Allen Mueller, Martin Apgar, Tommy Bruington, Joe Stashick, Leo Serebryannyy, Mike Castro, Jennifer Wang

2019nCoV-301 Study Group (Pubmed listed, in alphabetical order of institution affiliation)

Affiliation/Funding*	Study Group	Location
**México**		
Centro de Atención e Investigación Médica (CAIMED)	Jorge F. Méndez Galván, MD, Monica B. Carrascal, Adriana Sordo Duran, Laura Ruy Sanchez Guerrero, Martha Cecilia Gómora Madrid	Mexico City, Mexico
FAICIC Clinical Research	Alejandro Quintín Barrat Hernández, MD, Sharzhaad Molina Guizar, Denisse Alejandra González Estrada, Silvano Omar Martínez Pérez, MD, Zindy Yazmín Zárate Hinojosa, MD	Veracruz, Mexico
Instituto Nacional de Ciencias Médicas y Nutrición Salvador Zubirán	Guillermo Miguel Ruiz-Palacios, MD	Mexico City, Mexico
Instituto Nacional de Salud Pública	Aurelio Cruz-Valdez, PhD, Janeth, Pacheco-Flores, MD, Anyela Lara, MD, Secia Díaz-Miralrio	Cuernavaca, Mexico
PanAmerican Clinical Research México	María José Reyes Fentanes, MD, Jocelyn Zuleica Olmos Vega, MD, Daniela Pineda Méndez, MD, Karina Cano Martínez, MD, Winniberg Stephany Alvarez León	Querétaro, Mexico
PanAmerican Clinical Research México	Vida Veronica Ruiz Herrera, MD, Eduardo Gabriel Vázquez Saldaña, Laura Julia Camacho Choza, Karen Sofia Vega Orozco, Sandra Janeth Ortega Domínguez	Guadalajara, Mexico
Unidad de Atención Médica e Investigación en Salud (UNAMIS)	Jorge A. Chacón, MD, Juan J. Rivera, MD, Erika A. Cutz, MD, Maricruz E. Ortegón, MD, María I. Rivera, MD	Mérida, Mexico
**United States and Puerto Rico**		
Accellacare	David Browder, MD, Cortney Burch, Terri Moye, Paul Bondy, MD, Lesley Browder, MD	Rocky Mount, NC
Accellacare	Rickey D. Manning, MD, James Wilson Hurst, MD, Rodney E. Sturgeon, MD, Paul H. Wakefield, MD, John A. Kirby, MD	Knoxville, TN
Accel Research Sites	James Andersen, MD, Szheckera Fearon, MSN, FNP-C, Rosa Negron, MD, Amy Medina, ADN, BS	Lakeland, FL
Accel Research Sites	Bruce Rankin, DO, John M. Hill, MD, Steven Shinn, MD, Vivek Rajasekhar, DO, Marshall Nash, MD	DeLand, FL
Achieve Clinical Research	Hayes Williams, MD, PhD, LaShondra Cade, Rhodna Fouts, Connie Moya	Birmingham, AL
Alliance for Multispecialty Research	Corey G. Anderson, MD, Naomi Devine, NP-C, James Ramsey, NP-C, Ashley Perez, David Tatelbaum	Tempe, AZ
Alliance for Multispecialty Research	Michael Jacobs, MD, Kathleen Menasche, LPN, Vincent Mirkil, MD	Las Vegas, NV
Anaheim Clinical Trials	Peter J. Winkle, MD, Amina Z. Haggag, MD, Michelle Haynes, Marysol Villegas, Sabina Raja	Anaheim, CA
Atlanta Center for Medical Research	Robert Riesenberg, MD, Stanford Plavin, MD, Mark Lerman, MD, Leana Woodside, DNP, NP-C, Maria Johnson, MD	Atlanta, GA
Baylor College of Medicine / NIAID (UM1AI148575)	C. Mary Healy, MD, Jennifer A. Whitaker, MD, Hana El Sahly, MD, Christine Akamine, MD, Wendy A. Keitel, MD, Robert L. Atmar, MD	Houston, TX
Biomedical Advanced Research and Development Authority (BARDA)	Richard Gorman, MD, Gary Horwith, MD, Robin Mason, MS, MBA	Washington, DC
Benchmark Research	Laurence Chu, MD, Michelle Chouteau, MD, Lisa Johnson, FNP, Tambra Dora	Austin, TX
Benchmark Research	Greg Hachigian, MD, Deborah Murray, FNP, Michael Cancilla, PA, Logan Ledbetter, PA, Masaru Oshita, MD	Sacramento, CA
Benchmark Research	William Seger, MD, Beverly Ewing, APRN, DNP, FNP-BC	Fort Worth, TX
Beth Israel Deaconess Medical Center / NIAID (UM1AI068614)	Kathryn E. Stephenson, MD, MPH, Chen Sabrina Tan, MD, Rebecca Zash, MD, Jessica L. Ansel, MSN, Kate Jaegle, MSN, Caitlin J. Guiney, MSN	Boston, MA
Black Hills Center for American Indian Health / Missouri Breaks Industries Research Inc / NIAID (UM1AI068614)	Jeffrey A. Henderson, MD, MPH, Marcia O'Leary, RN, Kendra Enright, RN, Jill Kessler, MS, Pete Ducheneaux, LPN, Asha Inniss, MS, APRN	Eagle Butte, SD
California Research Foundation	Donald M. Brandon, MD, William B. Davis, MD, Daniel T. Lawler, MD	San Diego, CA
Carolina Institute for Clinical Research	Yaa D. Oppong, MD, Ryan P. Starr, DO, Scott N. Syndergaard, DO, Rozeli Shelly, MD, Mashrur Islam Majumder	Fayetteville, NC
Cedar Crosse Research Center	Danny Sugimoto, MD, Jeffrey Dugas Sr., MD, Dolores Rijos, Sandra Shelton, Stephan Hong, MD	Chicago, IL
Cenexel RCA	Howard Schwartz, MD, Nelia Sanchez-Crespo, MD, Jennifer Schwartz, APRN, Terry Piedra, BS, Barbara Corral, APRN	Hollywood, FL
Centex Studies	Joel Solis, MD, Carmen Medina, PA, Westley Keating, PA	McAllen, TX
Clinical Neuroscience Solutions	Michael E. Dever, MD, Mitul Shah, MD, Michael Delgado, MD, Tameika Scott, DrPH	Orlando, FL
Clinical Neuroscience Solutions	Lisa S. Usdan, MD, Lora J. McGill, MD, Valerie K. Arnold, MD, Carolyn Scatamacchia, MSN, NP-C, Codi M. Anthony, DNP, APRN, PMHNP-BC	Memphis, TN
CommonSpirit Health Research Institute	Rajan Merchant, MD, Anelgine Crans Yoon, MD, Janet Hill, PA-C, Lucy Ng-Price, MA, Teri Thompson-Seim	Woodland, CA
Comprehensive Clinical Research	Ronald Ackerman, MD, Jamie Ackerman, Florida Aristy, APRN	West Palm Beach, FL
Covid-19 Prevention Network (CoVPN)	Lawrence Corey, MD, Kathleen M Neuzil, MD, MPH, Huub G Gelderblom, MD, PhD, Nzeera Ketter, Carrie Sopher	Seattle, WA
CRA Headlands	Jon Finley, MD, Nathan Segall, MD, Mildred Stull, APRN, FNP-C	Stockbridge, GA
DM Clinical Research	Vicki E. Miller, MD, MPH, Monica Murray, Blanca Gomez, Zainab Rizvi, Sonia Guerrero	Tomball, TX
Empire Clinical Research	Yogesh K. Paliwal, MD, Amit Paliwal, MD, Sarah Gordon, MS, Bryan Gordon, Cynthia Montano-Pereira	Pomona, CA
Headlands Research	Christopher Galloway, MD, Candice Montros, Lily Aleman, Samira Shairi, RN, Wesley Van Ever	Orlando, FL
Health Research of Hampton Roads	George H. Freeman, MD, Esther Laverne Harmon, ANP, Marshall A. Cross, MD, Kacie Sales, BSN, RN, Catherine Q. Gular, PharmD	Newport News, VA
HHS-DoD Countermeasures Acceleration Group	Matthew Hepburn, MD	Washington, DC

HOPE Research Institute	Matthew Doust, MD, Nathan Alderson, PhD, Shana Harshell	Phoenix, AZ
Howard University Hospital / Howard University College of Medicine / NIAID (UM1AI068614)	Siham Mahgoub, MD, Celia Maxwell, MD, Thomas Mellman, MD, Karl M Thompson, PhD, Glenn Wortman, MD	Washington, DC
IACT Health	Jeff Kingsley, DO, April Pixler, LaKondria Curry, Sarah Afework, Austin Swanson	Columbus, GA
Jacksonville Center for Clinical Research	Jeffry Jacqmein, MD, Maggie Bowers, PA-C, Dawn Robison, APRN-C, Victoria Mosteller, MD, Janet Garvey, DNP	Jacksonville, FL
Johnson County Clin-Trials	Carlos Fierro, MD, Mary Easley, BSN, RN	Lenexa, KS
Joint Program Executive Office for Chemical, Biological, Radiological and Nuclear Defense's, US Department of Defense	Rebecca J. Kurnat	Washington, DC
Lynn Health Science Institute	Carl P. Griffin, MD, Raymond Cornelison, MD, Shanda Gower, APRN, CNP, William Schnitz, MD, Destiny S. Heinzig-Cartwright, BA	Oklahoma City, OK
Lynn Institute of the Ozarks	Derek Lewis, MD, Fred E. Newton, MD, Aeiress Duhart, Breana Watkins, Brandy Ball	Little Rock, AR
Lynn Institute of the Rockies	Ripley Hollister, MD, Jeremy Brown, DO, Melody Ronk, PA-C, Jill York, Shelby Pickle	Colorado Springs, CO
M3-Emerging Medical Research	David B. Musante, MD, William P. Silver, MD, Linda R. Belhorn, MD, Nicholas A. Viens, MD, David Dellaero, MD	Durham, NC
M3-Wake Research	Matthew Hong, MD, Wayne Harper, MD, Lisa Cohen, DO, Priti Patel, NP, Kendra Lisec, PA	Raleigh, NC
MD Clinical	Beth Safirstein, MD, Luz Zapata, MD, Lazaro Gonzalez, APRN, Evelyn Quevedo, APRN, Farah Irani, PhD	Hallandale Beach, FL
Medical Research International	Joseph Grillo, MD, Amy Potts, PA-C, MPH, Julie White, MBA	Oklahoma City, OK
Medical University of South Carolina	Patrick Flume, MD, Gary Headden, MD, Brandie Taylor, NP, Ashley Warden, Amy Chamberlain	Charleston, SC
MedPharmics	Robert Jeanfreau MD, Susan Jeanfreau MD	Metairie, LA
MedPharmics	Paul G. Matherne, MD, Amy Caldwell, RN, Jessica Stahl, Mandy Vowell, Lauren Newhouse	Gulfport, MS
Meharry Medical College / NIAID (UM1AI068614)	Vladimir Berthaud MD, MPH, Zudi-Mwak Takizala MD, MPH, MBA, Genevieve Beninati, FNP, Kimberly Snell, PharmD, Sherrie Baker, BS, James Walker, RN	Nashville, TN
Meridian Clinical Research	David Ensz, MD, Tavane Harrison, CNP, Meagan Miller, Janet Otto	Sioux City, IA
Meridian Clinical Research	Brandon Essink, MD, Roni Gray, APRN, Christine Wilson, Tiffany Nemecek, Hannah Harrington, MPH	Omaha, NE
Meridian Clinical Research	Charles Harper, MD, Keith Vrbicky, MD, Chelsie Nutsch, NP, Sally Eppenbach, NP, Wendell Lewis, NP	Norfolk, NE
Meridian Clinical Research	Jordan Whatley, MD, Christopher Dedon, APRN, FNP-C, Tana Bourgeois, RN, Lyndsea Folsom, Crystal Rowell, APRN, FNP-C	Baton Rouge, LA
Miami Veterans Affairs Medical Center / NIAID (UM1AI068614)	Gregory Holt, MD, Mehdi Mirsaeidi, MD, Rafael Calderon, MD, Paola Lichtenberger, MD, Jalima Quintero, RN, Becky Martinez, RN	Miami, FL
Morehouse School of Medicine / NIAID (UM1AI068614)	Lilly Immergluck, MD, Erica Johnson, PhD, Austin Chan, MD, Norberto Fas, MD, LaTeshia Thomas-Seaton, MS, APRN, Saadia Khizer, MD, MPH	Atlanta, GA
MultiCare Institute for Research and Innovation	Jonathan Staben, MD	Cheney, WA
National Institute of Allergy and Infectious Diseases (NIAID) / National Institutes of Health (NIH)	Tatiana Beresnev, MD, Maryam Jahromi, MD, Mary A. Marovich, MD, Julia Hutter, MD, Martha Nason, PhD, Julie Ledgerwood, DO, John Mascola, MD	Bethesda, MD
National Research Institute	Mark Leibowitz, MD, Fernanda Morales, Mike Delgado, Rosario Sanchez, Norma Vega	Los Angeles, CA
Novavax, Inc.	Lisa M. Dunkle, MD, Germán Áñez, MD, Gary Albert, Erin Coston, Chinar Desai, Haoua Dunbar, Mark Eickhoff, Jenina Garcia, Margaret Kautz, Angela Lee, Maggie Lewis, Alice McGarry, Irene McKnight, Joy Nelson, Patrick Newingham, Patty Price-Abbott, Patty Reed, Diana Vegas, Bethanie Wilkinson, PhD, Katherine Smith, MD, Wayne Woo, MS, Iksung Cho, MS, Gregory M. Glenn, MD, Filip Dubovsky, MD, MPH	Gaithersburg, MD
Omega Medical Research	David L. Fried, MD, Lynne A. Haughey, MSN, FNP, Ariana C. Stanton, PA-C, Lisa Stevens Rameaka, MD	Warwick, RI
Pharmacology Research Institute	David Rosenberg, MD, Lee Tomatsu, Viviana Gonzalez, Millie Manalo	Los Alamitos, CA
PMG Research of Bristol	Bernard Grunstra, MD, Donald Quinn, MD, Phillip Claybrook, MD, Shelby Olds, MD, Amy Dye	Bristol, TN
PMG Research of Wilmington	Kevin D. Cannon, MD, Mesha M. Chadwick, MD, Bailey Jordan, Morgan Hussey, Hannah Nevarez	Wilmington, NC
Ponce de Leon Center / NIAID (UM1AI068614)	Colleen F. Kelley, MD, MPH, Valeria D. Cantos MD, Michael Chung MD, Caitlin Moran, MD, MSc, Paulina Rebolledo, MD, Christina Bacher, PAC	Atlanta, GA
Ponce School of Medicine / NIAID (UM1AI148685)	Elizabeth Barranco-Santana, MD, Jessica Rodriguez, MD, Rafael Mendoza, MD, Karen Ruperto, MD, Odette Olivieri, MD, Enrique Ocaña, MD	Ponce, Puerto Rico
Preferred Research Partners	Paul E. Wylie, MD, Renea Henderson, DO, Natasa Jenson, MD, Fan Yang, MD, Amy Kelley, BSN, RN	Little Rock, AR
Providea Health Partners Elligo Health Research	Kenneth Finkelstein, DO, David Beckmann, MD, Tanya Hutchins, FNP, Sebastian Garcia Escallon, BA, Kristen Johnson	Evergreen Park, IL
Providence Clinical Research	Teresa S. Sligh, MD, Parul Desai, NP, Vincent Huynh, BSc, Carlos Lopez, MD, Erika Mendoza, BA	North Hollywood, CA
Research Your Health	Jeffrey Adelglass, MD, Jerome (Jerry) G. Naifeh, MD, Kristine Jane Kucera, PA-C, MPAS, DHS, Waseem Chughtai, BS, MBBS, Shireen Hasham Jaffer	Plano, TX
Rochester Clinical Research	Matthew G. Davis, MD, Jennifer Foley, Michelle Lyn Burgett, RN, Tammi Louise Shlotzhauer, MD, Sarah Michelle Ingalsbe-Geno, RPA-C	Rochester, NY
SIMEDHealth / SIMEDResearch	Daniel Duncanson, MD, Kelly Kush, Lori Nesbitt, Cora Sonnier, Jennifer McCarter	Gainesville, FL

Sterling Research Group	Michael B. Butcher, MD, James Fry, PA-C, Donna Percy, RN, BSN, Karen Freudemann	Cincinnati, OH
Sterling Research Group	Bruce C. Gebhardt, MD, Padma N. Mangu, MD, Debra Beck Schroeck, MS, PA-C, Rajesh Kumar Davit, MD, Gayle D. Hennekes, PA-C, MPAS	Cincinnati, OH
Stony Brook University - Stony Brook Medicine / NIAID (UM1AI068614)	Benjamin J. Luft, MD, Melissa Carr, BA, Sharon Nachman, MD, Alison Pellecchia, BA, Candace Smith, PharmD, Bruno Valenti, NP	Commack, NY
Suncoast Research Associates	Maria I. Bermudez, MD, Noris Peraita, ARNP, Ernesto Delgado, ARNP, Alicia Arrazcaeta, Natalie Ramirez	Miami, FL
Suncoast Research Group	Mark E. Kutner, MD, Jorge Caso, MD, Janet Mendez, ARNP, Marianela Carvajal, ARNP, Carmen Amador, ARNP	Miami, FL
Sundance Clinical Research	Larkin Tyler Wadsworth III, MD, Horacio Marafioti, MD, Lyly Dang, DNP-BC, Lauren Clement, NP-C, Jennifer Berry, FNP-BC	St. Louis, MO
Synexus Clinical Research	Mohammed Allaw, MD, Georgettea Geuss, Chelsea Miles, NP, Zachary Bittner, Melody Werne	Evansville, IN
Synexus Clinical Research	Cornell Calinescu, MD, Shannon Rodman, Joshua Rindt	Henderson, NV
Synexus Clinical Research	Erin Cooksey, MD, Kristina Harrison, Deanna Cooper, Manisha Horton Amanda Philyaw	Anderson, SC
Synexus Clinical Research	William Jennings, MD, Hilario Alvarado, MD, Michele Baka, MD, Malina Regalado, NP	San Antonio, TX
Synexus Clinical Research	Linda Murray, DO	Pinellas Park, FL
Synexus Clinical Research	Sherif Naguib, MD, Justin Singletary, Sha-Wanda Richmond, Sarah Omodele, Emily Oppenheim	Atlanta, GA
Synexus Clinical Research	Joseph Newberg, MD, Laura Pearlman, MD, Reuben Martinez, Victoria Andriulis	Chicago, IL
Synexus Clinical Research	Paul J. Nugent, DO, Leonard Singer, MD, Jeanne Blevins, Meagan Thomas, Christine Hull	Cincinnati, OH
Synexus Clinical Research	Isabel Pereira, MD, Gina Rivero, Tracy Okonya, Frances Downing, Paulina Miller	Vista, CA
Synexus Clinical Research	Margaret Rhee, MD, Katherine Stapleton, Jeffrey Klein, Rosamond Hong, MD	Akron, OH
Synexus Clinical Research	Suzanne Swan, MD, Tami Wahlin, MD, Elizabeth Bennett, PA, Amy Salzl Sharine Phan	Richfield, MN
Synexus Clinical Research	Jewel Johnny White, MD, Amanda Occhino, Ruth Paiano APRN, Morgan McLaughlin APRN, Elisa Swieboda APRN	The Villages, FL
Texas Center for Drug Development	Veronica Garcia-Fragoso, MD, Maria Gabriela Becerra, MD, Cecilia Mckeown, Lisa Holloway, Toni White	Houston, TX
The Charlotte-Mecklenburg Hospital Authority d/b/a Atrium Health / NIAID (UM1AI068614)	Christine B. Turley, MD, Andrew McWilliams, MD, Tiffany Esinhart, PA-C, Natasha Montoya, APRN, Shamika Huskey, FNP, Leena Paul, FNP	Charlotte, NC
The Miriam Hospital / NIAID (UM1AI068636)	Karen Tashima, MD, Jennie Johnson, MD, Marguerite Neill, MD, Martha Sanchez, MD, Natasha Rybak, MD, Maria Mileno, MD	Providence, RI
UC Davis Health / NIAID (UM1AI068614)	Stuart H. Cohen, MD, Monica Ruiz, Dean M. Boswell, BS, Elizabeth E. Robison, BS, Trina L. Reynolds, BS, Sonja Neumeister, MPH	Sacramento, CA
Universidad de Puerto Rico - Recinto de Ciencias Médicas - Maternal Infant Studies Center (CEMI) / NIAID (UM1AI068636)	Carmen D. Zorrilla, MD, Juana Rivera, MD, MPH, Jessica Ibarra, MD, Iris García, BSN, RN, Dianca Sierra, BA, Wanda Ramon, BSPh	San Juan, Puerto Rico
University of Colorado Hospital CRS / NIAID (UM1AI068636) / NCATS (UL1TR002535, UM1AI069432)	Thomas B. Campbell, MD, Suzanne Fiorillo, MSPH, Rebecca Pitotti, RNP, Victoria Riedel Anderson, MS, Jose Castillo Mancilla, MD, Nga Le, PharmD	Aurora, CO
University of Iowa Medical Center / NIAID (UM1AI068614) / NCATS (UL1TR002537)	Patricia L. Winokur, MD, Dilek Ince, MD, Theresa Hegmann, PA, Jeffrey Meier, MD, Jack Stapleton, MD, Laura Stulken, PA	Iowa City, IA
University of Maryland School of Medicine / NIAID (UM1AI148689)	Monica McArthur, MD, PhD, Karen L. Kotloff, MD, Kathleen Neuzil, MD, Andrea Berry, MD, Milagritos Tapia, MD, Elizabeth Hammershaimb, MD, MS, Toni Robinson, RN, Rosa MacBryde, RN	Baltimore, MD
University of Minnesota / NIAID (UM1AI068614)	Susan Kline, MD, MPH, Joanne L. Billings, MD, MPH, Winston Cavert, MD, Les B. Forgosh. MD, Timothy W. Schacker, MD, Tyler D. Bold, MD, PhD	Minneapolis, MN
University of Missouri Health Care / NIAID (UM1AI148685)	Dima Dandachi, MD, MPH, Taylor Nelson, DO, Andres Bran, MD, Grant Geiger, S. Hasan Naqvi, MD	Columbia, MO
University of Nebraska Medical Center / NIAID (UM1AI068614)	Diana F Florescu, MD, Richard Starlin, MD, David Kline, MD, Andrea Zimmer, MD, Anum Abbas, MD, Natasha Wilson, APRN	Omaha, NE
University of North Carolina / NIAID (UM1AI068619) / University of North Carolina at Chapel Hill Center for AIDS Research (P30AI050410) / NC TraCS Institute (UL1TR002489)	Cynthia L. Gay, MD, MPH, Joseph J Eron, MD, Michael Sciaudone, MD, MPH, A. Lina Rosengren, MD, MPH, MS, John S Kizer, MD, Sarah E Rutstein, MD, PhD	Chapel Hill, NC
University of South Florida, Morsani College of Medine / NIAID (UM1AI068614)	Carina A. Rodriguez, MD, Elizabeth Bruce, MD, Claudia Espinosa, MD, Lisa J Sanders, MD, Kami Kim, MD, Denise Casey, RN	Tampa, FL
University of Texas Health Science Center San Antonio / NIAID (UM1AI068614)	Barbara S. Taylor, MD, MS, Thomas Patterson, MD, Ruth Serrano Pinilla, MD, Delia Bullock, MD, Philip Ponce, MD, Jan Patterson, MD	San Antonio, TX
University of Washington / Lummi Tribal Health Center / NIAID (UM1AI148573)	R. Scott McClelland, MD, MPH, Dakotah C. Lane, MD, Anna Wald, MD, MPH, Frank James, MD, Elizabeth Duke, MD, Kirsten Hauge, MPH, Jessica Heimonen, MPH	Seattle, WA
University of Washington	Robert W. Coombs, MD, PhD, Alex Greninger, MD, PhD, MS, MPhil, Pavitra Roychoudhury, PhD, Erin A. Goecker, MS, Yunda Huang, PhD, Youyi Fong, PhD	Seattle, WA
VA Ann Arbor Healthcare System / NIAID (UM1AI068614)	Carol Kauffman, MD, Kathleen Linder, MD, Kimberly Nofz, BSN, Andrew McConnell, BS	Ann Arbor, MI

Velocity Clinical Research	Robert J. Buynak, MD, Angella Webb, APRN, Taryn Petty, FNP, Stephanie Andree, FNP	Valparaiso, IN
Velocity Clinical Research	Judith Kirstein, MD, Marcia Bernard, Erica Sanchez, Nolan Mackey, Clarisse Baudelaire	Banning, CA
Velocity Clinical Research	Gregg Lucksinger, MD, Jaleh Ostovar, NP	Medford, OR
Velocity Clinical Research	Mary Beth Manning, MD, Joan Rothenberg, MD, Toby Briskin, MD, Denise Roadman, PAC, Sarah Dzigiel	Cleveland, OH
Velocity Clinical Research	J. Scott Overcash, MD, Adrienna Marquez, Hanh Chu, Kia Lee, Kim Quillin	La Mesa, CA
Velocity Clinical Research	Barbara Rizzardi, MD, Michelle King, NP, Vanessa Abad, NP, Jennifer Knowles, BS	West Jordan, UT
Velocity Clinical Research	Michael Waters, MD, Karla Zepeda, NP, Scott Overcash, MD, Jordan Coslet, NP, Dalia Tovar, MA	Chula Vista, CA
Velocity Clinical Research	Marian E. Shaw, MD, Mark A. Turner, MD, Cory J. Huffine, FNP-C, Esther S. Huffine, FNP-C	Meridian, ID
Walter Reed Army Institute of Research	Julie A. Ake, MD, MSc	Silver Spring, MD
Wayne State University / NIAID (UM1AI068614)	Elizabeth Secord, MD, Eric McGrath, MD, Phillip Levy, MD, Brittany Stewart, RD, PharmD, Charnell Cromer, RN, MSN, Ayanna Walters, RN, BSN	Detroit, MI
Weill Cornell Chelsea CRS / NIAID (UM1AI068619)	Kristen Marks, MS, MD, Grant Ellsworth, MD, MS, Caroline Greene, ANP-BC, Sarah Galloway, BA, Shashi Kapadia, MD, MS, Elliot DeHaan, MD	New York, NY
Willis-Knighton Health System / WKB Family Medicine Associates	Clint Wilson, MD, Jason Milligan, MD, Danielle Raley, MD, Joseph Bocchini, MD	Bossier City, LA
Womack Army Medical Center	Bruce McClenathan, MD, Mary Hussain, BS, Evelyn Lomasney, MD, Evelyn Hall, MMS, PA-C, Sherry Lamberth, PharmD	Fort Bragg, NC
WR Clinsearch	Mark McKenzie, MD, Teresa Deese, Christy Schmeck, Vickie Leathers, Christy Sweet	Chattanooga, TN

* Funding of institutions by the National Institute of Allergy and Infectious Diseases (NIAID) and/or research support by the National Center for Advancing Translational Science (NCATS), as indicated. All other institutions were funded by Office of the Assistant Secretary for Preparedness and Response, Biomedical Advanced Research and Development Authority. The content of this publication is solely the responsibility of the authors and does not necessarily represent the official views of the funding sources.

2019nCoV-301 Principal Investigators and Study Team (in alphabetical order)

Principal Investigator	Study Team	Institution	Location
Ronald Ackerman, MD	Jamie Ackerman, Florida Aristy, Tomeko Heard, Diana Mann, Maureen Stewart, Cheryl Demczyk, Rohan Barron, Ashley Torres, Jennifer Gomez, Tiffany Potter	Comprehensive Clinical Research	West Palm Beach, FL
Jeffrey Adelglass, MD	Jerome (Jerry) G. Naifeh, Kristine Jane Kucera, Waseem Chughtai, Shireen Hasham Jaffer, Anuja Sathe, Cameron Galownia, Cheryl Hill, Ramiro Lopez, Erica Parker-Martinez, Helene Harrison, Chiedza Mutindori, Sabrina Flowers, Tamara Betters, Carolyn Ackley, Pamela Fox, Noelia Tejada James, Dorothy Saylor, Hallen Dao, Jon Etta Randolph, Jason Tentativa, Malaika Chughtai, Shanzae Chughtai, Maheen Shah, Hayyan Chughtai, Tyler Love, Ti'arah Love	Research Your Health	Plano, TX
Mohammed Allaw, MD	Georgettea Geuss, Chelsea Miles, Zachary Bittner, Melody Werne, Lyndsey Morrison, Stephanie Albin, Linda Frazier, Jacque Nalley, Christie Borin, Jacque Nalley	Synexus Clinical Research	Evansville, IN
James Andersen, MD	Szheckera Fearon, Rosa Negron, Amy Medina, Diana Holmes, Colleen Figueroa, Cristal Ruiz, Nancy Masseus Tare Floyd, Kenta Oliver, Candice Gerber, Mae Ann Francisco, Gilbert de la Cruz, Ginny McClanahan, Veronica Walker, David Irwin, Gloria Adejobi	Accel Research Sites	Lakeland, FL
Corey G. Anderson, MD	Naomi Devine, James Ramsey, Tyanna Montijo, Ashley Perez, David Tatelbaum, Lisa M. Dean, Angela D. Ledezma, Anthony Padilla, Cecilia M. Tanori, Georgina Lopez-Wood, Tasha C. Marriott, Ronald Hawkins, Hannah Spinks	Alliance for Multispecialty Research	Tempe, AZ
Elizabeth Barranco-Santana, MD	Michele Irizarry, Alice Grace Rodriguez, Irmaris Arroyo, Sara Cancel, Alejandra Román, Juan D. Lugo, Armando X. Torres, Marianne Hernandez, Brenda Garcia, Nancy Jiménez, Orlando Torres	Ponce School of Medicine	Ponce, Puerto Rico
Alejandro Quintín Barrat Hernández, MD	Sharzhaad Molina Guizar, Denisse Alejandra González Estrada, Silvano Omar Martínez Pérez, Zindy Yazmín Zárate Hinojosa, Norberto Daniel Vázquez Tinajero, Yessica Olivo Domínguez, Daniel Hernández León, Gloria Norma Ambrosio Lara, José Carlos Mateos Castro, Irving Nerí Leyva Ferrer, María Fernanda Hernández García, Heidy Jazmín Maldonado Pavón, Evelyn Monserrat Bravo Serralta, Edgar Iván Muñoz López, Karina Esmeralda García Mateo, Lorena Cruz Cruz, José Javier Zárate Hinojosa, Javier Torres Cole, Yareth Jiménez Barcenas, Andrea Anaid Rangel Huerta, Erika Guillén González, María de la Luz Rufina Martínez Lugo, Angélica Liliana Muñoz Solano, David Sena Gómez, Berenice Valera Montalvo, Moisés Miguel Ruíz Nogueira, Yoshira Montero Díaz, Francisco Javier Martínez Osorio, Alejandra Morales Arias, Sandra Itzel Solis Rivera, Alejandro Esteban Cortina, Aldo Miguel López Domínguez, María Fernanda Cortés Ruíz, Marilyn Yulissa Ramírez Domínguez, Lucero Moctezuma Juan, Francisco Barrales Arcos	FAICIC Clinical Research	Veracruz, Mexico
Maria I. Bermudez, MD	Noris Peraita, Ernesto Delgado, Alicia Arrazcaeta, Natalie Ramirez, Giovanna Salcedo, Aliana Amador, Elizabeth Martinez, Arleen Aspuru, Gabriella Gonzalez, Gabriella Alabaci, Livan Sanchez, Raul Tejeda, Adriana Bello, Barbara Vega-Aguera, Kassandra Martinez, Grettel Obregon, Oscar Alejandro Gutierrez Luna, Magela C. Dominguez, Lauren Pena	Suncoast Research Associates	Miami, FL
Vladimir Berthaud, MD, MPH	Toni Hall, Livette Johnson, Sylvia Eluhu, Ana Tomescu, Katharina Whitbeck, Rajbir Singh	Meharry Medical College	Nashville, TN
Donald M. Brandon, MD	William B. Davis, Daniel T. Lawler, Maria Aceves, Kathleen B. Anderson, Hana Berry, Janice E. Brandon, Jeffrey C. Brandon, Patricia A. Brandon, Lorraine Boggs, Charlene Cruz, Mairead Hawkins, Clarice Hranicky, Andrew J. McCrea, Karen G. McCrea, Kimberly Najera, Tierney J. O'Connor, Michelle L. Rios, Cindy F. Stevens, Hannah J. Zapata	California Research Foundation	San Diego, CA
David Browder, MD	Cortney Burch, Terri Moye, Michael Wright, Paul Bondy, Lesley Browder	Accellacare	Rocky Mount, NC
Michael B. Butcher, MD	James Fry, Julia Froschauer, Allison Deuel, Jeanne Piccola, Donna Percy, Karen Freudemann, Lois Rawe, Megan Bryant, Kurt Percy, Jon Marvin, Luann Corcoran	Sterling Research Group	Cincinnati, OH
Robert J. Buynak, MD	Mark Yarosz, Rachel McNeal, Megan Smith, Patricia Volom, Nicholas Hanna, Erica Lewis, Miranda Lee, Goldie Luna, Marilyn Idowu, Destiny Williams, Jessica Johnson, Consuelita Perez, Priscilla Dodson	Velocity Clinical Research	Valparaiso, IN
Cornell Calinescu, MD	Shannon Rodman, Joshua Rindt, Krystal Tyner, Lovelyn Vincente, Alejandro Osuna-Meda, Charmaine Brown, Matthew Derrick, Melodee Morrison, Marissa Washington	Synexus	Henderson, NV
Thomas B. Campbell, MD	Donna McGregor, Laurel Ware, Myron Levin, Steven Johnson, Sophia Quesada, Martin Krsak, Kristine Erlandson, Nicholas Sarchet, Vanessa Sutton, Lawrence Moran, Tracey Stevenson, Alaina Dougherty, Julianne Randlemon	University of Colorado Hospital CRS	Aurora, CO
Kevin D. Cannon, MD	Mesha M. Chadwick, Bailey Jordan, Taylor Fedorcha, Kathryn Zweier, Brettany Holt, Emily Johnson, Karen Ruggiero, Olivia Houghton, Courtney Christie, Allison Dunn, Courtney Boyce, Sasha Saint-Lot, Ashley Andrades, Ashley Miller, LaShaya Dunston, Russell Larkins, Brittany Savoca, Hannah Nevarez, Hannah Nevarez, Larkin Collins, Morgan Cyrus, Morgan Hussey, Christina MacNaughton, Heidi Kaufman, Sheila Gard, Alyssa Gaylor, Bethany Donelan-Wilson, Taylor Bayless, Anna McManus, Tracie Marlowe Bryant, Ben Manuel, Laura McMillan, Nicole Stigers, Prerana Zanke	PMG Research of Wilmington	Wilmington, NC
Jorge A. Chacon, MD	Juan J. Rivera, Erika A. Cutz, Maricruz E. Ortegon, María I. Rivera, Ricardo Cervera, Felipe Rivera, Daniela Pat, Daniela Cruz, Alberto Chacon, Kattia Borges, Aldo Borraz, Rebeca Ortegon, Karla Ic, Carmen Ojeda, Irvin Ortega, Mayra Jimenez, Cindy Novelo, Pharmacist, Mónica Pérez, Adriana Hernandez, Laura Martinez	Unidad de Atención Medica e Investigación en Salud (UNAMIS)	Merida, Mexico
Laurence Chu, MD	Michelle Chouteau, Lisa Johnson, Tambra Dora, Lamar Box, Michelle Listz, Katherine Davis, Jennifer Montes, Jessica Ruff, Jennifer Leyva, Pamela Fidler, Ruth Fitch, Sean Turnbow, Francesca Vigil, Maria Barrientes, Isaiah Knight, Cindy Duran, Lauren Christal, Breana Wade Liaison, Brooke Harris, Dean Skiles, Marisol Ramos, Brandon Newsom, Candace Gaitan, David Pereira	Benchmark Research	Austin, TX

Stuart H. Cohen, MD	Curtis Blankenship, Katelyn Trigg, Courtney Lymuel, Gursimran Mann, Zayan Musa, Hana Minsky, Eliseo Vasquez, Nicole Garza, Kaitlyn Low, Mehrab Hussain, William Li, Rahul Araza, Monique Conover, George Thompson, Hien Nguyen, Scott Crabtree, Bennett Penn, Minh-Vu Nguyen, Archana Reddy, Derek Bays, Kaitlyn Hardin, Matthew Boutros, Alan Koff, Natascha Tuznik, Angel Desai, Naomi Hauser, Sarah Waldman, Gauri Barlingay, Dean Blumberg	UC Davis Health	Sacramento, CA
Erin Cooksey, MD	Kristina Harrison, Deanna Cooper, Manisha Horton, Amanda Philyaw	Synexus Clinical Research	Anderson, SC
Aurelio Cruz-Valdez, PhD	Janeth, Pacheco-Flores, Anyela Lara, Secia Diaz-Miralrio	Instituto Nacional de Salud Pública	Cuernavaca, México
Dima Dandachi, MD, MPH	Tami Day, Britlyn Brown, Taylor Mathews	University of Missouri Health Care	Columbia, MO
Matthew G. Davis, MD	Therese Dayton, Joseph I. Mann, Patricia S. Larrabee, Jean C. Kelly, Tia. L Albro, Zerina Zornic, Susan J. Willer, Donna M. Willome, Kathleen K. Ebeling, Jaclyn P. Zona, Julie A. Mooney, Katherine A. Pagenkemper, Victoria F. Fink, Christine N. Hall, Chelsea Bork, Abigail Miller, Mackay Kanaley, Chelsey LoMonaco, Marie Musolino, Jessica Fisher, Katilyn Bergen, Rachel Bordonaro, Cassidy Glod, Liam Sullivan, Brandi Douglass, Ann Casey, Philip LaSpino, Maurice Holmes	Rochester Clinical Research	Rochester, NY
Michael E. Dever, MD	Michael Delgado, Tameika Scott, Laverne Denise Davila, Nelisa Frias, Anissa Hilton, Patricia Brown, Shana Caldwell, Martha Hendrix, Edmund Delgado, Mitul Shah, Gracemarie Rosario, Kaneitra Williamson, Taylor Lucier, Jaime Hawat, Matthew Stephens, Monica Cooper, Dante Canidate, Denise Pagan, Sierra Robinson, Pascal Nelson-Quiles, Anthony Perez, Chanel Adams, Keisha Foster, Scott Salmon, Andrew Lockwood, Priya Moorhouse, Paul Yi	Clinical Neuroscience Solutions	Orlando, FL
Matthew Doust, MD	Stephanie Catanzaro, Shana Harshell, Madison Mikulak, Bettie, D'Nise Corcoran, Susan DeCraene, Jasmin Redden, Brian DeCraene, Karen Wakefield, Adrian Aljeo, Denise Sample, Clarissa Lara, Stephanie Junker, Nathan Alderson, Kimberly Joshlin, Mia Munoz, Michele Aguirre, Dina Reyes Cordova, Neil Pearson	HOPE Research Institute	Phoenix, AZ
Daniel Duncanson, MD	Kelly Kush, Lori Nesbitt, Cora Sonnier, Jennifer McCarter, Thomas Buschbacher, Evie Zavala, Brittany Cooper, Abbey Mannings, Melissa Berrio, Erin Juhl, William Douglas, Timothy Elder, Linda Grover, Colleen Crabbe, Rachel Francis, Jesse Lipnick, Seldon Longley, Michael Rozboril, Madison Duncanson, Jakob Vaes, Michael Costa, Dhruv Panchal, Michelle Hendricks, Sergio Montalvo, Angel Dubois	SIMEDHealth / SIMEDResearch	Gainesville, FL
David Ensz, MD	Bruce Rankin, Tavane Harrison, Meagan Miller, Kayla Sturgeon, Jessica Knight, Janet Otto, Monica Salazar, Megan Howard, Carly Deges, Joseph Harris, Rylea Gulick, Melissa Wiseman, Sue Doty	Meridian Clinical Research	Sioux City, IA
Brandon Essink, MD	Roni Gray, Christine Wilson, Fritz Raiser, Akossiwa "Essi" Yovogan, Jessica Satorie Tiffany Nemecek, Hannah Harrington, Amy Lett-Brown,Chelsea Steinmetz, Tabitha Campbell, Carrie Essink, Jamie Meyer, Riley Brockman, Melissa Monarrez, Troy Humphries, Wynter Huffman, Brooke Dworak, Raquel Davis, Samantha Nocita, Heidi Smith, Carissa Schejbal, Kayla Flege, Joe Genoways, Jessa Swanson, Avery Dunn, Kevin Grimes, Phillip Astorino, Ashtynn Jarosz, Hailey Harper, Amy Nichols, Azra Bauman, Jessica Fellows, Courtney Heisey, Ginny McNew	Meridian Clinical Research	Omaha, NE
Carlos Fierro, MD	Natalia Leistner, Amy Thompson, Celia Gonzalez, Nathan Arthur, Mazen Zari, Mary Easley, Heather Barker, Manyvohn Rinehart, Monica Atwood, Natalya Amrine, Kelly Moen, Kaley Miller, Angela Eichler, Ann Geier, Christa Estrada, Amber Wolf, Denise Essix, Latoria Rios, Kasie Hickert, Kenny Nguyen, Karol Moore, Stefanie Uwah, Kaelyn Howell, Miranda Dean	Johnson County Clin-Trials	Lenexa, KS
Kenneth Finkelstein, DO	David Beckmann, Tanya Hutchins, Sebastian Garcia Escallon, Kristen Johnson, Athena Rivera, David Otuada, Jessica Bartlett, Lauren Wade, Tyler Will, Gina Nielsen-Grewe, Anita Suri	Providea Health Partners Elligo Health Research	Evergreen Park, IL
Jon Finley, MD	Nathan Segall, Mildred Stull, Michelle Sowell, Michelle Binns, Kiara Tyner, Karen Yangapatty, Elizabeth West, Cynthia Steele, Kwannda Whatley, Hannah Smith, Pamela Talbott, Kimberly Cobb, Donna Toepfer, Jennifer LeBrun, Susan Jones, Patrizia Greene, Cynthia Pinckney, Kim Banaski, Karen Hickson	CRA Headlands	Stockbridge, GA
Diana F Florescu, MD	Mark Rupp, Daniel Brailita, Adia Sikyta, Erica Stohs, Sara Hurtado Bares, Nada Fadul, Matthew Lunning, Elizabeth Schnaubelt, Molly Ferris, Andrew Buettner, Matthew Palmer, Bailee Lichter, Alison Lewis, Chase Kimberling, Jonathan Beck, Erin Iselin, Kimmai McClain, Andrew Schnaubelt	University of Nebraska Medical Center	Omaha, NE
Patrick Flume, MD	Gary Headden, Brandie Taylor, Ashley Warden, Amy Chamberlain, Kim Spencer, April Rasberry, Angela Millare, Angel Darrow, Abbey Grady, Max Lento, Allison Patterson, Caitlan LeMatty, Jhonatan Diaz, Andrew Stephens, Emalee Wood, Destri Eichman, Annie Cribb, Annelise Kauffman<br>Charnele Handy, Elizabeth Poindexter, Moira Chance, Anna Miller, Elizabeth Dickinson, Andrea Boan, Erin Klintworth	Medical University of South Carolina	Charleston, SC
Veronica Garcia-Fragoso, MD	Maria Gabriela Becerra, Cecilia Mckeown, Lisa Holloway, Toni White, Bonnie Colville, Frederic Santiago, Teresa Becker, Shakira Barr, Chen Ho Yang, Tracy Kowalski, Danitra Glasper, Diana Chehab Nazanin Zarinkamar, Joanna Quezon, Maryam Rabbani, Sadaf Batla, Ayla Perez, Berenice Ferrero, Dean Jang, Biman Goswami, Dustin McFadden, Elton Oliveira, Enya Rentas-Sherman, Julian Edmonson, Laura Plaza-Grisanty, Olga Konshina, Rachely Araujo-Gutierrez, Scott Ward, Teodoro Seminario, Patricia Matute, Sauleha Husain, Akram Assaf, Elisa Moralez, Frances Saubon, Jenny Torres, William Fernandez, Ashraf Jafri, Amy Anderson, Saji Mathew Perinjelil, Waheeda Sureshbabu, Kara Sikes, Joel Cano, Kendra Rogers, Quiana Wilson, Karina Sainz, Abdeali Dalal, Leena Mir, Misbah Baloch, Shammarrian Hampton, Crystal Reese, Lucia Almaguer,<br>Felicia Ardoin, Deep Patel, Bernardo Martinez Leal, Faryal Mahmood, Ana Rueda, Norma Gonzalez, Stacey Montero, Chandra Tobin, Abyssinia Moges, Ari Amirkhosravi, Herman	Texas Center for Drug Development	Houston, TX

	Ortiz, Matthew Joseph, Parul Mehta, Zain Rizvi, Diego Carrington, Blessing Feliz-Okoroji, Moez Talpur, Robert Krbashyan, Simeen Khan, Mary Rogers		
George H. Freeman, MD	Esther Laverne Harmon, Marshall A. Cross, Kacie Sales, Catherine Q. Gular, Amanda Fronzaglio, Timothy O'Malley, Zaahin Huq, Jenna Johnson, Jessica Fuggett, Danielle Merian, Rita Quinn	Health Research of Hampton Roads	Newport News, VA
David L. Fried, MD	Lynne A. Haughey, Ariana C. Stanton, Lisa Stevens Rameaka	Omega Medical Research	Warwick, RI
Christopher Galloway, MD	Candice Montros, Lily Aleman, Samira Shairi, Robert Duran, Wesley Van Ever, Wasilah Suid, Sandra Torres, Taylor Rice, Wanda Estrada, Julie Castillo,Stephanie Cassidy, Ashleigh Ford, Thai Marie, Colon Maldonado, Amedaris Cordero, Zahra Somji, Rachel Morris	Headlands Research	Orlando, FL
Cynthia L. Gay, MD, MPH	David Wohl, Michelle Floris-Moore, Michael Herce, Danielle Clement, Arianna Morrison, Jan Busby-Whitehead, Michelle Hernandez, Zachary Willis, Allison, Burbank, Peyton Thompson, Chris Evans, Susan Pedersen, Becky Straub, Samantha Earnhardt, Erin Hoffman, Jonathan Oakes, Tevnan Keller, Victoria Rucinski, Camille O'Reilly, Kelsey Vollmer, Jennifer Rees, April Welch, Patti Vasquez, Joy Wannamaker, Tanailly Giralt Smith, India Pitts, Amanda Beaten, Ebony Harrington, Alex Bradley, Chidinma Okafor, Miriam Chicurel-Bayard, Kristina Shoffner, Polly Tsai, Chelsea Taylor, Susanne Hendersen, Emily Padgett, Debbie Pence, Jane Salm, Matt Campbell, Kirsten Haigler, Ekatherina Diadiuk, Mariam Ramzan, Pamela Miller, Julie Nelson, Nicole Maponga, Carmen Garcia, Charlie McGehee, Gloria Oyediran, Paul Alabanza, William Wolf, Hannah Munro, Rachael Turner, Dana Lapple, Grace Tillotson, Andrew Powell, Mandy Tipton, Catherine Kronk, Oesa Vinesette, Arti Malik, Kirby Caraballo, Maria Stetson, Charles West, Erin Cardot, Andy Thorne, Maria Bullis, William Zhao, Jennifer Thompson, Kristen Gray, Sarah Law, Holly Milner, Frederick Asamoah, Daniel Galeana, Marcia Gibson, Caressa Goss, Pamela Jones, Joshua Lee, Cheryl Hendrickson, Rachel Cook, Erin Daniel, Centhla Washington, Carolina Pastrana-Medina, Dayo Nylander-Thompson, William Johnson, Eliza Debose, Chloe Twomey, Rachel White, Grace Bailey, Hayley Meier, Jennifer Te Vazquez, Ascary Arias, Allison Castillo, Dynesha Perry, Gwen McKnight, Lucie Mangala, Jessica Gingles, Maggie Harman, Marie Oriol, Sean McMurray, Christy Litel, Noshima Darden-Tabb, Yerson Padilla, Danna Frederick	University of North Carolina	Chapel Hill, NC
Bruce C. Gebhardt, MD	Padma N. Mangu, Debra Beck Schroeck, Rajesh Kumar Davit, Gayle D. Hennekes, Donna Percy	Sterling Research Group	Cincinnati, OH
Carl P. Griffin, MD	Raymond Cornelison, Shanda Gower,William Schnitz, Angela Genovese, Ryan Morgan, Destiny S. Heinzig-Cartwright, April Green, Kim Hamilton, Chalimar Rojo, Lacey Dietz, Sharee Wright, Aja George, Karen Hames, Sharla Lister, Brandy Ball, Andrea Romero, Krystal Hightower, Dalia Tovar, Kim Calloway, Samelia Farni, Chris Hyatt, Linda Lopez, Kathi Shaw, Natacha Tull, Katelyn Hughes, Selwyn Oruh, Lauren Schwab, Samantha Ting	Lynn Health Science Institute	Oklahoma City, OK
Joseph Grillo, MD	Amy Potts, Julie White, Carla Bender, Debra Daugomah, Caitlin Harris, Brian White, Alannah Hill, Chelsea Lairson, Karen Blevins	Medical Research International	Oklahoma City, OK
Bernard Grunstra, MD	Donald Quinn, Shelby Olds, Phillip Claybrook, Amy Dye, Shai Perry, Joshua Bullen, Jennie Eller, Sandy Daggs, Nicole Everhart, Dennis Lee, Farrah Fuston	PMG Research of Bristol	Bristol, TN
Greg Hachigian, MD	Deborah Murray, Michael Cancilla, Logan Ledbetter, Masaru Oshita	Benchmark Research	Sacramento, CA
Charles Harper MD	Keith Vrbicky, Chelsie Nutsch, Sally Eppenbach, Wendell Lewis, Alisha Kiepke, Misty Appeldorn, Cyla Rohde, Catherine King, Kayla Andal, Ashley Frisch, Courtney Green, Kelsey Kelley, Katlyn Mace, Jordan Suckstorf, Torie Johnson, Linden DeBoer, Christy Lee, Eric Graber, Jeni Hoppe, Jill Smith, Heather Ebel, Taysha Hingst, Samantha Wieseler, Diahn Pekny, Elijah Schantz	Meridian Clinical Research	Norfolk, NE
C. Mary Healy, MD	Chianti Wade Bowers, Chanei Henry, Sheri Ordonez, Janet Brown, Cathy Faw, Shetel Anassi, Trent Davis, Kim Taylor	Baylor College of Medicine	Houston, TX
Jeffrey A. Henderson, MD, MPH	Jeffrey A. Henderson, MD, MPH, Marcia O'Leary, RN, Kendra Enright, RN, Jill Kessler, MS, Pete Ducheneaux, LPN, Asha Inniss, MS, APRN	Black Hills Center for American Indian Health / Missouri Breaks Industries Research Inc	Rapid City, SD
Ripley Hollister, MD	Jeremy Brown, Melody Ronk, Jill York, Shelby Pickle, Jami Wagner, Lisa Jackson, Felipa Ramdeholl, Angelica Romero	Lynn Institute of the Rockies	Colorado Springs, CO
Matthew Hong, MD	Wayne Harper, Lisa Cohen, Priti Patel, Kendra Lisec, Makayla Dutton, Lynn Eckert, Aubrey Farray, Jenee Jiggetts, Emily Reilly, Jill Holmes, Aaron Deaver, Christine Grissom, Judith Shand, Brianca Farmer, Eric Henderson, Kristen Shireman, Brad Muskelley, Franziska Gassaway, Darian Lawrance, Sabine Ucik, Toni Bland, Katedra Dixon, Reginald Santiago, Caroline Zhu, Kathleen Sander, Brian Joseph, Marsha Peery, Lori Bridges, Sadia Khan, Adnan Nasir, Sofia Sequiera, Raquell Messick, Kyra Brown, James Hull	M3-Wake Research	Raleigh, NC
Gregory Holt, MD	Jennifer Denizard, Juanita Johnson, Sehrish Sikandar, Gisel Urdaneta, Silvana Cobain, Melyssa Sueiro, Precious Leaks, Evelyn Guadalupe, Rochelle Thompson, Dexter Peart, Leidi Paez, Krystal Hosang, Runxia Tian, Ali Vaeli Zadeh	Miami Veterans Affairs Medical Center	Miami, FL
Lilly Immergluck, MD	LaKesha Tables, Harold Gene Stringer, Jacquelyn Ali, Cristina Wilson, Noor Mohamed, Kay Woodson, Tiffany White	Morehouse School of Medicine	Atlanta, GA
Michael Jacobs, MD	Kathleen Menasche, Vincent Mirkil,Yazil Ramirez, Michael Yee, Laura Elio, Candice Garcia, Azucena Valdovino, Cristina Garcia, Sharla Peahi-Ching, Kristina Arcos	Alliance for Multispecialty Research	Las Vegas, NV
Jeffry Jacqmein, MD	Maggie Bowers, Dawn Robison,Victoria Mosteller, Janet Garvey, Alpa Patel, Darlene Bartilucci, Kenneth Aung-Din, Margaret Gannaway, Carolyn Tran, Michael Koren, Mitchell Rothstein, Sonia Gerardo, Cassie Lawler, Yvonne Douglas, Chris Ganzhorn, Emery Noles,	Jacksonville Center for Clinical Research	Jacksonville, FL

	Angela Morris, Lisa Carl, Andrea West, Laura Little, Ramil Castillo, Abbey Ras, Nalini Jones, Annan Nurrenbern, Deirdre Arrington, Jacob Wolfer, Brenda Anderson, Amanda Elwood, Amber DeVries, Cara Seifart, Jimmy Knowles, Vy Dang, Mary Strickland, Pam Garmon, Caron Whitelaw, Sharon Smith, Ivy Guillermo, Nate Grant, Khatija Hussein, Caron Whitelaw RN, Bernadette Moineau, Robert Nix		
Robert Jeanfreau, MD	Susan Jeanfreau, Katelyn Jackson, Kynisha "Nicki" Johnson, RaeShanta McKendall, Shonna James, Calisha Sadiq, Susan Tortorich, Lori Goins, Steven Darden, Melissa Spedale, Kristen Robinson, Joseph Favret, Yordanka Koleva, April Spears, David Conroy	MedPharmics	Metairie, LA
William Jennings, MD	Hilario Alvarado, Michele Baka, Malina Regalado	Synexus Clinical Research	San Antonio, TX
Carol Kauffman, MD	Andrea Starnes, Andrea Woods, Karen Brudzinski	VA Ann Arbor Healthcare System	Ann Arbor, MI
Colleen F. Kelley, MD, MPH	Carlos del Rio, Sheetal Kandiah, Catherine Abrams, Erin Andrew, Felicia Atkinson, Erica Baker, Juliet Brown, Tucker Colvin, Natasha Renee Cook, Meena Dhir, Christopher Foster, Ronald Gaston, Gabriela Gerogial, John Gharbin, Betsy Hall, Valarie Hunter, Aastha KC, Kelly Likos, Bezuayehu Mandefro, Myles Mason, Humberto Orozco, Isaac Perez, Philip Powers, Christin Root, Brittany Spiegel, Pamela Weizel, Sarah Wiatrek, Felicia Wright	Ponce de Leon Center	Atlanta, GA
Jeff Kingsley, DO	April Pixler, LaKondria Curry, Sarah Afework, Austin Swanson, Alyssa Middlebrook, Christine Senn, Keyrhea Ritter, Katlin Salewski, Sierra Holmes, Jean Niles, Taylor Hernandez, Lacey Shaw, Kaila Maddox, Klarissa Bohnstedt, Emily Gilder, Cassandra Motley, Alyssa Middlebrook, Hephzibah Udo, Mattison Sherer, Wayman Petty, Joseph Surber	IACT Health	Columbus, GA
Judith Kirstein, MD	Marcia Bernard, Erica Sanchez, Nolan Mackey, Clarisse Baudelaire, Hanna He, Brenda Delgado, Brandon Steppe, Bonnie Goodale, Nicole Abels, Carol Remigio, Dipal Patel, Emily Zacarias, Nuvia Espinoza, Esmeralda Machado, Katia Talamante, Lizeth Romero	Velocity Clinical Research	Banning, CA
Susan Kline, MD, MPH	Sara Eischen, Rebecca Cote, Diondra Howard, Editha Jordan, Joyce Bolea, Annie McFarland, Asfaw Mesfin, Andrew Snyder, Darlette Luke, Derek LaBar, Theresa Christiansen, Beth Jorgenson, Christina Glasgow, Melissa Schedler	University of Minnesota	Minneapolis, MN
Mark E. Kutner, MD	Mark E. Kutner, Jorge Caso, Janet Mendez, Maria Hernandez, Carmen Amador, Amanda G. Colina, Alain Chang, Alondra Diaz, Arael Ayala, Carmen Ballester, Claudia Rodriguez, Dalila Del Valle, Eduardo Rodriguez, Gloria Moreno, Jennifer Ortega, Jhobana Vargas, Jonathan Fernandez, Juan Carlos Delgado, Laura Gonzalez, Leidy Montoya, Marianela Carvajal, Mariete Rendon, Maury Santos, Michelle Browne Mirnaya, Mujica, Neiner Enriquez, Noelio Hernandez, Paola Garcia<br>Raydel Valdes, Saray Carvajal, Susel M. Figueredo, Vanessa Hechevarria, Yanelis Dominguez, Yusleidy Diaz	Suncoast Research Group	Miami, FL
Mark Leibowitz, MD	Fernanda Morales, Rosario Sanchez, Mike Delgado, Norma Vega, Nelly Ayala, Iliana Gallaga, Cassandra Celis, Jennifer Muniz, Mariela Quiroz, Juan Frias, Rea Abaniel, John Nelson, Maricor Grio, Alejandro Moreno, Coralia Soto, Jose Espino, Daniel Vargas, Stephanie Lopez	National Research Institute	Los Angeles, CA
Derek Lewis, MD	Fred Newton, Aeiress Duhart, Breana Watkins, April Green, Chala Simpson, Briana Dean, Brandy Ball, Shakita Stevenson, Lashonda Stephenson	Lynn Institute of the Ozarks	Little Rock, AR
Gregg Lucksinger, MD	Jaleh Ostovar, Audrey Kuehl, Viviana Juncal, Avery Kerwin	Velocity Clinical Research	Medford, OR
Benjamin J. Luft, MD	Jorge Alves, Melissa Carr, Ryan Chacon, Barsha Chakraborhy, Aymon Faizi, Laurel Gumpert, Andrew Handel, Kayla Henkel, Erin Infanzon, Andrew Kanner, Lily Limsuvanrot, Michelle Miroddi, Jeanine Morelli, Sharon Nachman, Rena Nanan, Alexander Newman, Alison Pellecchia, Trisha Rush, Jennifer Russell, Stephanie Santiago-Michels, Jonathan Sicoli, Candace Smith, Michael Truhlar, Bruno Valenti, Jennifer Valentine, Kathy Vivas, Yasmine Brown-Williams	Stony Brook University - Stony Brook Medicine	Commack, NY
Siham Mahgoub, MD	Alice Ukaegbu, Immaculate Okonkwo, Shannon Gopaul, Tara Gibbons, Yuanxiu Chen, Debra Ordor, Linda Fletcher, Megan Ware, Florencia Gonzalez, Michael Perini, Carla Williams, Mulu Mengistab, Robert Postell, Yejide Obisesan, Adetokunbo Adedokun, Reyneir Magee, Jeremy Smith, Edward Bauer, Lora Collins, Urelda Allman, Deborah Clements, Sarah Shami, Nathaniel Blaboe, Pedro Lima, Michael Crawford	Howard University Hospital / Howard University College of Medicine	Washington, DC
Mary Beth Manning, MD	Toby Briskin, Denise Roadman, Sarah Dzigiel, Jennifer Gaston, Brooke Glivar, Brianna Arman, Briana Jackson, Brian Sharpe, Naqib Ahmad, Nicole Baitt	Velocity Clinical Cleveland	Cleveland, OH
Rickey D. Manning, MD	James Wilson Hurst, Rodney E. Sturgeon, Paul H. Wakefield, John A. Kirby	Accellacare	Knoxville, TN
Kristen Marks, MS, MD	Marshall Glesby, Roy Gulick, Timothy Wilkin, Ole Vielemeyer, Mary Vogler, Carrie Johnston, Rebecca Fry, Daniel Finn, Caitlin Rhoades, Noah Goss, Shaun Barcavage, Valery Hughes, Jonathan Berardi, Ashley Machado, Caique Mello, Mia Crowley, Monique Williams, Minkyung Lee, Mary Ann Zwiebel, Patrice Weller, Antonio Rivera-Lopez, Harrison Chan, Ruby Lee, Victoria Lesina, Vasilika Koci, Paul Kim, Steven Wang, Malissa Robinson, Edward Kenny, Danny Garcia, Venus Fernandez, Parul Shah, Celine Arar, Byron Bullough, Jonattan Rodriguez, Jessenia Fuentes, Jiamin Li, Arthur Goldbach, Genessi Rodriguez, Catherine Jerry, Nadi Islam, Madeline Gomez, Rajshri Hirpara, Ioanna Pahountis, Wayne Burns, Tahera Begum, Gianna Resso, Sophia Alvarez, Elizabeth Connolly, Roxanne Rosario, Sierra Derti, Britta Witting, Anna Gwak	Weill Cornell Chelsea CRS	New York, NY
Paul G. Matherne, MD	Cassie Beeks, Sarah Bowen, Deven Fejka, Nicole Guttierrez, Lakeyla Bates, Pam Taylor, Gigi Benoit, Micki Le	Medpharmics	Gulfport, MS
Monica McArthur, MD, PhD	Cheryl Young, Helen Powell, Levis Contreras, Panagiota Komninou, Christine Wade, Jumoke Oladapo, Kaitlin Mason, Robin Barnes, Leslie Howe, Cheilon Bolanos, Shannon Bittner, Elva Valle-Maldonado, Wanda Somrajit, Biraj Shrestha, Justin Ortiz, Nancy Greenberg, Kathleen Strauss, Lisa Chrisley, Melissa Billington, Sudhaunshu Joshi, Lavida Porter, Megan McGilvray, Daryl Grays, Shirley George, Jennifer Marron, Kelly Brooks, Natelaine Fripp, Mardi Reymann, Brenda Dorsey, Patricia Farley, Melissa Myers, Natasha	University of Maryland School of Medicine	Baltimore, MD

	Harris, Alyson Kwon, Marcela Pasetti, Daniel Cohen, Myounghee Lee, Laura Liberman, Sherry McCammon, James Campbell, Ana Herbert, Julia Silva, DeAnna Friedman-Klabanoff, Alythia Vo, Jennifer Winkler, Lisa Turek, Colleen Boyce, Anne Thurston, Daniele Nitkowski, Ginny Cummings, Sandra Molina, Susan Holian, Matthew Laurens, Rekha Rapaka, Megan Deming, Mark Travassos, Kirsten Lyke, Henry Seifert, George Escobar, Norma Martinez, Abigail Arias, Ana Maria Davila, Dolores Fontalvo, Elsa Aracely Vargas, Elva Jaldin, Gladis Lopez, Irma Justiniano, Judith Tenezaca, Julio Fernandez, Luz de Maria Osorio, Maria de los Angeles Pichardo, Morena Lemus, Maria Elena Rocha, Rosa Angelica Vigil, Sandra Herrera		
R. Scott McClelland, MD, MPH	Devinder Garcha, Christopher P. Hawk, Bonnie Duran, Donna Lodge-Moore, Leigh Tao, Cristina J. Toledo-Cornell, Mona Jalili, Susan Lottimer, Sheila Samra, Seslee Alsup, Karlee Cooper, Theresa George-Greene, Spencer Hanson, Joni Hensley, Emily Barnett Highleyman, Jewell Jefferson, Jessica Lane, Jessica Long, Alex Martinez, Kerri Sloan, Kelly Smith, Kristee Lewis, Tara Babu, Dwyn Dithmer, Matthew Dustrude, McKenna Eastment, Emily Ford, Abir Hussein, Christine Johnston, Pamela Kohler, Debra Metter, Thepthara N. Pholsena, Meredith Potochnic, Tara Reid, Miko Robertson, Michelle Sabo, Helen Stankiewicz Karita, Jina Taub, Dana Varon, , Brian Wood, Alyssa Braun, David Crawford, Mark Drummond, Jess Heimonen, Lawrence Hemingway, Madelaine Humphreys, Bianca Kalia, Mary Kirk, Taylor Krause, Ray Larsen, Gisella Logioia, Cristina Luevano Santos, Anya Mathur, Lindsey McClellan, Jessica Moreno, Nicole Roed, Matthew Seymour, Katie Wicklander	University of Washington & Lummi Tribal Health Center	Seattle, WA
Bruce McClenathan, MD	Mary Hussain, Aaron Poch, Amy Santangelo, Anne Poch, David De Blasio, Evelyn Lomasney, Jacob Turnquist, Kathryn Lago, Laurie Housel, Sherry Lamberth, Sheryl Bedno, Lauren Blevins, Laura Brown, Alice Clay, Gervon Collins, Kaitlyn Covington, Amy Davis, Patricia Davis, Nicole Friedberg, Lacey Gazlay, Helen Gooden, Evelyn Hall, Kim Locklear, Kayla Majors, Shamona McRae, Bryce Meerhaeghe, Kendalyn Stephens, Jade Tran, Lisette Watkins, Katie Williams, Kema Matthews, Karrie Greive, Brandi Carroll, Amanda Williams, Brittany Garner, DeLisa Crosby, Jennifer Ritschl, Jamie Frahm, Karen Stewart, Priti Patel, Dilay Uras, Allison Northrop, Anika Uson, Olyvia Ray, CynDavia McKoy, April Beals, Deidre Turner, Christina Spooner	Womack Army Medical Center	Fort Bragg, NC
Mark McKenzie, MD	Teresa Deese, Christy Schmeck, Vickie Leathers, Christy Sweet, Misti Earwood, Erica Osmundse, Gisela Heintz, Lilian Nunkuna, Michelle Forgey, Shelly Brooks, Justian Jarrett, Elizabeth Michael, Lisa Guider, Zack Harmon, Diane Sproles, Randy Cooper, Jessica Benvenuto, Stefanie Mullins, Quinetrice Bennett, Corey Flack	WR Clinsearch	Chattanooga, TN
Jorge F. Méndez Galván, MD	Adriana Sordo Durán, Martha Yarelli Valencia Mejía, Froylan David Martínez Sánchez, Ana María Piña Rodríguez, Diana Alim Mena Martínez, Melany Susel Fernández Valdez, Laura Ruy Sánchez Guerrero, Ana Fabiola Ruiz Villagrana, Mónica B. Carrascal, Martha Cecilia Gomora Madrid, Anahí García Álvarez, Ismael Delgado Ginebra, Omar Alfonso Heredia Nieto, Yanni Maldonado Ventura, Jonathan E. Ramírez Salazar, Mariela Salgado Zagal, María Fernanda Espinosa García, Yolanda Albor Hernández, Ricardo Antonio González, Germán Alonso Lara, Marbella Rojas Ortega, Bernardo Kleinfinger Chayet, Victor Emmanuel Alva López, Diego Carlos Angel Perez, Alejandro Cortes Meda,Ivonne Hernandez Giron	Centro de Atención e Investigación Médica (CAIMED)	Mexico City, Mexico
Rajan Merchant, MD	Anelgine Crans Yoon, Janet Hill, Lucy Ng-Price, Teri Thompson-Seim, Alejandra Cazares Hernandez, Danielle Hornbuckle, Adriane Rubit, Ann Campbell, Dawn Diorio, Adeline Stabler, Jasdeep Shergill, Claudia Gross, Anne Nguyen	CommonSpirit Health Research Institute	Woodland, CA
Vicki E. Miller, MD, MPH	Amy Starr, Shiela Varghese, Sonia Guerrero, Monica Murray, Vanessa Gonzales, Blanca Gomez, Zainab Rizvi, Victoria Aguilar, Anna Pena, Madiha Baig, Dustin Watson, Pauline Ngban, Afifah Ayub, Laura Drampou, Shelby Danforth, Diana Avalos, Jacquelyn Gonzales, Ragen Powell, Sajjad Naqyi, Ambily Dileep, Alefiyah Motiwala, Heather Leary, Humera Siddiqui, Miatta George, Kastyn Kelly, Nicole Segura, Maryam Jamil, Husain Motiwala, Sandra Smith, Sally Hussein, Yousra Yousif, Carlyn Robinson, Cannon Lenfield, Luis Leal, Muhammad Irfan, Nayab Croher, Pattie Tate, Sandra Natalia Perez, Fredric Santiago, Syeda Riaz, Arsani Iskandar, Alefya Hussain	DM Clinical Research	Tomball, TX
Linda Murray, DO	Christy Delcamp, Monica Hoewt, Kristin Shade, Tara McTigue	Synexus Clinical Research	Pinellas Park, FL
David Musante, MD	William P. Silver, Linda R. Belhorn, Nicholas A. Viens, David Dellaero, Shandelle Parker, Andrew Zimmerman, Roger Ordronneau, Bryan Stanislaus, Kevrin Johnson, Megan Dice, Megan Heron, Sarah Wilkerson	M3-Emerging Medical Research	Durham, NC
Sherif Naguib, MD	Justin Singletary, Sha-Wanda Richmond, Sarah Omodele, Emily Oppenheim, Jalisha Hemphill, Marqueta Jones, Millat Gedefa, Janean Smith, Bonnie Raufman, Lesley Whitehead, Elia O'Dell, Sarah Omodele, David Taylor, ShaWanda Richmond, Alexis Melson, Justin Singletary	Synexus Clinical Research	Atlanta, GA
Joseph Newberg, MD	Laura Pearlman, Reuben Martinez, Victoria Andriulis, Jacquilyn McCormick, Anna Maddox, Rosalinda Vazquez, Nicole Leahy, Marian Padilla, Mary Reyes	Synexus Clinical Research	Chicago, IL
Paul J. Nugent, DO	Leonard Singer, Jeanne Blevins, Meagan Thomas, Christine Hull	Synexus Clinical Research	Cincinnati, OH
Yaa D. Oppong, MD	Ryan P. Starr, Scott N. Syndergaard, Nafisa Saleem, Cheryl Norris, Nicole Austin, Rozeli Shelly, Md Mashrur Islam Majumder, Annette Bunnells, Michelle Wallace, Avia McClain-Stocker, Rachel Ryan, Katie Wood, Arien Stebbins, Crystal Schmitt, Jeffrey Pemberton, Mitchel Arlidsen, Daniel Tomita, Geraldine McRae, Amy Sheets, Jeanette Mangual-Coughlin,<br>Margo Miller-Smith, Melinda Thomas	Carolina Institute for Clinical Research	Fayetteville, NC
J. Scott Overcash, MD	Adrienna Marquez, Hanh Chu, Kia Lee, Kim Quillin, Jordan Coslet, Yashveer Dubbula, Adam Prince, John Rodriguez, Lee Tomatsu, Erin Vawter, Michael Voskanian, Michael Waters, Gina Weaver, Karla Zepeda, Angela Anorve, Gordon Bovee, Jennifer Baker, Laura Castillo, Allie Davis, Jacob Esparza, Andrea Garcia, Jessica Gonzales, Lizette Gonzalez,	Velocity Clinical Research	La Mesa, CA

	Ashleigh Lindsay, Erica Marinelli, Cathy Meza, Shandel Odom, Makenna Orel, Grecia Perez, Helen Pu, Cesar Ramirez, Melania Riordan, Deidre Romines, Raquel Taitingfong, Katrina Tyler, Bernadette Wilson		
Yogesh K. Paliwal, MD	Amit Paliwal, Renu Bhupathy, Krystle Edwards, Sarah Gordon, Cynthia Montano-Pereira, Blanca Gomez, Yazmin Nunez, Cassandra Martinez, Connie Navarrete, Mayra Casas, Ysabel Lopez, Anthony Macias, Alexandria Vasquez, Maria Gomez	Empire Clinical Research	Pomona, CA
Isabel Pereira, MD	Gina Rivero, Tracy Okonya, Frances Downing, Paulina Miller, Yasmin Camberos	Synexus Clinical Research	Vista, CA
Bruce Rankin, DO	John M. Hill, Steven Shinn, Vivek Rajasekhar, Marshall Nash, Michelle Tutt, Kimberlee Del Campo, Douglas F. Winter, Leandro Fernandez, Melissa Hodges, Michelle Jones, Sean Lemoine, Veronica Walker, Roy D. Richardson, Angeline Petracca. Katina Marchione, Michelle Morgan, Ashley McCaffrey, Amber Vasquez, Amy Houck-Dominy, Angela Hammerle, Antonio Rivera, Claxton Copeland, Crystal Paccione, Diana Toney, Fadhel Alyunis, Jennifer Dittman, Kriston Applewhite, Lora Parahovnik, Over Seijas, Ryan Hobbick, Samantha Watts, Shatonia Fields, Stacie Evans, Teresa Logsdon, Thais Truffa, Tifany Huertas, Vienna Bauer,William Serrano, Daisy Sawyer, Giovanni Urquilla, Tonya Toby, Albert Garcia, Alicia J. Cevera,Jeffery Hood, Hannah Hodges, Melissa Willard	Accel Research Sites	DeLand, FL
María José Reyes Fentanes, MD	Pablo Fermín González Limón, Luis Ricardo Acosta Beuló, Paulina Cleer García Valdovinos, Olivia de la Puente Flores, Eduardo Rugama Martel, Ana Gabriela Mier Flores, Ulises Abel Rodríguez Vargas, Diego Guillermo Muñoz Bolaños, Martha Alejandra Alonso Trejo, Elvia Ramírez Gutiérrez, Alberto Aaron del Rosal Medina, Jaime Chavez Baron, Ana Gabriela Guizar Zamora, Felipe Arredondo Saldaña, Juan De Dios Martín Luján Palacios, Juan José Pardo Moreno, Jorge Torres Ferrera, Itzetl Guzman Mendieta	PanAmerican Clinical Research México	Querétaro, Mexico
Margaret Rhee, MD	Jeffrey Klein, Katherine Stapleton, Stacy Collins, Dawn Greer, Kelli Meissner, Brenda Moore, Tylene Falkner, Celeste Blazy, Nicole Johnson, Christina Carter, Annette Pangle, Rosamond Hong	Synexus Clinical Research	Akron, OH
Robert Riesenberg, MD	Robert Riesenberg, Stanford Plavin, Mark Lerman, Leana Woodside, Maria Johnson	Atlanta Center for Medical Research	Atlanta, GA
Barbara Rizzardi, MD	Michelle King, Vanessa Abad, Jennifer Knowles, Benjamin Richeson, Denise Pessetto, Heather Holtman, Lori Luth, Wyatt Walsh, Andrea Johnson, Dreama Fackrell, Patrick O'Keefe, Sara Isolampi, Michelle Walkingshaw, Josh Carrillo, Renu Landage, Stephanie Wallace	Velocity Clinical Research	West Jordan, UT
Carina A. Rodriguez, MD	Patricia Emmanuel, Lucy Guerra, Asa Oxner, Alicia Marion, Reed Ryan, Tiffany Vasey, Susannah Hall, Amanda Morton, Emma Gonzalez, Elisabeth Ballans, Rachel Karlnoski, Luz Santamaria, Rosalinda Cruz, Joshua Finley, Michael Hayes, Oliver Emberger, Mark Pennington, Meghana Vankatesh, Kimberly Johnson, Marina Wassif, Janelle Perkins, Veroniya Winkfield, Amavyvis Garcia, John Jones, Lori Brock, Kyle Cesareo, Dilcina Dragon, Dominic Moore, Catherine Marten, Thi Nguyen, April Roberts, Kristi Bojaxhi, Chrestenie Mouse	University of South Florida, Morsani College of Medicine	Tampa, FL
David Rosenberg, MD	Lee Tomatsu, Viviana Gonzalez, Millie Manalo, Nicole Rudin	Pharmacology Research Institute	Los Alamitos, CA
Vida Veronica Ruiz Herrera, MD	Vida Veronica Ruiz Herrera, Eduardo Gabriel Vazquez Saldaña, Laura Julia Camacho Choza, Karen Sofia Vega Orozco, Sandra Janeth Ortega DominguezMaria, Carolina Molina Roman, Julian Camacho Choza, Rodolfo Fabian Lomeli Guerrero, Giuliana Magaña Garcia, Carlos Andres Perez Navarro, Cesar Alberto Lopez Martin, Luis Arturo Rico Godinez, Felipe de Jesus Lopez Cordova, Daniel Arroniz Bernal, Luisana Aldaco Cota, Edgar Cordova Pulido, David Aguila Rivera	PanAmerican Clinical Research México	Guadalajara, Mexico
Beth Safirstein, MD	Luz Zapata, Lazaro Gonzalez, Evelyn Quevedo, Farah Irani, Julio Vigil, Steven Rapp, Mark Firestone, Humberto Mucientes, Ali Yasells Garcia, Florence Baum, Robert Hacman, Martha Ravelo, Carlos Alzate, Keyanna Francois, Alberto Napoles, Jamie Lorenzo, Deandra Clarke, Disneydi Gutierrez, Yean Alfonso, Nestor Lopez, Ana Bustos, Ilya Faybishenko, Lynnette Perez, Evelyn Qulies, Maria del Valle, Natalie Joseph, Judith Powell, Jessica Hernandez, Rafael Sanchez, William Torres, Damaris Alonso, Dragos Juravle, Roberto Valledor, Veronica Valledor, Maria Pazos, Teresa Rios, Maria Lascano	MD Clinical	Hallandale Beach, FL
Howard Schwartz, MD	Nelia Sanchez-Crespo, Terry Piedra, Barbara Corral, Jennifer Schwartz	Cenexel RCA	Hollywood, FL
Elizabeth Secord, MD	Roy Collins, Marita Poff, Jamal Chehab, Sajith Matthews, Thomas Mazzocco, Chantel Karmo, Sarah Meram, Janie Faris, Valerie Mika, Shobi Mathew, Brian O'Neil, James Paxton, Amy Stolinski, Stacie Smith, Benjamin Wasinski, Lisa Palmer, Katherine Cross, Samuel Ceckowski, Theodore Falcon, Jeffrey Harrison, Abe Lovelace, Selmir Mahmutovic	Wayne State University	Detroit, MI
Marian E. Shaw, MD	Mark A. Turner, Cory J. Huffine, Esther S. Huffine, Jacqueline Hanson, Nicholas Tuttle, Shannon Veach, Antonio Navarrete, Jammie Smith	Velocity Clinical Research	Meridian, ID
Teresa S. Sligh, MD	Scott Sligh, Parul Desai, Vincent Huynh, Carlos Lopez, Erika Mendoza, Dennis Perez, Samuel Ceballos, Jennifer Gomez, Janneth Becerra, Tiffany Martinez, Erika Navarro Fausto	Providence Clinical Research	North Hollywood, CA
Joel Solis, MD	Carmen Medina, Westley Keating	Centex Studies	McAllen, TX
Jonathan Staben, MD	Jessica Horton, Hannah Neill-Gubitz, Hilary Koenigs, Autumn Dlugas, Stacie Rebar, Anne Reedy, Roslyn Pierce, Kali Karst, Jaimee Gribben, Sarah Troutt, Mimi Meipel, Ann Carson, Paige Ramos, Natosha Hardy, Zack Brownell, Dot Heid, Annie Estes, Andrea Fry, Veronica Navarro	MultiCare Institute for Research and Innovation	Cheney, WA
Kathryn E. Stephenson, MD, MPH	Karen A. Lorenc, Audrey B. Nathanson, Michelle Beck, Shaelah M. Huntington, Wendy Hori, Uyen Rasphoumy, Ashley Beckles, Jody Dushay, Vijai Bhola, Wilanda Gabriel, Annika Gompers, Halle Hall, Nicholas Manickas-Hill, Toluwanimi Ajayi, Nicole Magner, Conor Cronin, James Arrico, Heena Patel, Janet Mullington, Michael Seaman, Katherine Yanosick, Ariana Leonelli, Eric Dai	Beth Israel Deaconess Medical Center	Boston, MA
Danny Sugimoto, MD	Jeffrey Dugas Sr., Dolores Rijos, Sandra Shelton, Stephan Hong	Cedar Crosse Research Center	Chicago, IL
Suzanne Swan, MD	Sharine Phan, Tami Wahlin, Elizabeth Bennett, Amy Salzl, Jeannette Blaisdell, Stacie Mahowald, Dominick Thibodeau, Sophia Houser, Tammy Hanson	Synexus Clinical Research	Richfield, MN

Karen Tashima, MD	Helen Patterson, Stacey Chapman, Giselle Pinto, Jennifer Brashears, Evelyn Hipolito, Laura Elmasian, Timothy Flanigan, Joseph Garland, Britt Harrington, Anthony Harrison, Jenny Thai, Mazen Taman, Krista Kiser, Kay Rutherford, Shivani Patel, Jimin Shin, Kim Rapoza, Sujata Sahu, Kristine Hauser, Kendra Vieira, Elliott Bosco, Christopher Federico, Kanika Malani, Christian Schroeder, Janet O'Connell, Meghan McCarthy, Anna Hippchen	The Miriam Hospital	Providence, RI
Barbara S. Taylor, MD, MS	Bhoja Katipally, Jessica Blower, Kimberly Kone Ellis, Heta Javeri, Danielle Dixon, Anna Taranova, Diana Cavazos, Robin Tragus, Irma Scholler, Lisa Longoria, Laura Najvar, Meredith Hosek, Bridgette Soileau, Morgan Brown	University of Texas Health Science Center San Antonio	San Antonio, TX
Christine B. Turley, MD	Lewis McCurdy, Tonisha Brown, Martha Pawlicki, Jennifer Reeves, Jona Bauer, Cedrick Griner, Cameron Russell, Veena Sampathkumar, Zeynep Alimchandani, Robin Muller, Tracey Coakley, Mary Sours, Saifelnasr Mohamed, Sone Alanoh, Amy Yeh, Sahra Khan, Eleojo Abutu, Genena Buck, Sarah Hicks, Andreana Alexander, Tammy Patterson, Maria Martilnsalaco, Amy Clontz, Marina Leonidas, Zainab Shahid, Jay I. Patel, Ryan Bender	The Charlotte-Mecklenburg Hospital Authority d/b/a Atrium Health	Charlotte, NC
Lisa S. Usdan, MD	Lora J. McGill, Valerie K. Arnold, Carolyn Scatamacchia, Codi M. Anthony, Carol R. Marsh, Cathy T. Houpt, Charles L. Grandberry, Debra A. O'Brien, Kelsey N. Evans, Leslie M. Lazar, Mary J. Williams, Megann F. Fickle, Robyn M. Presley, Shelby R. McWhorter, Julia Sinatra, Irene W. Powell, Tavia S. Flagg, Melissa N. Flowers, Penny J. McCracken, Reagan A. Boone, Dominique L. Ross, Amber J. Jones, LaKeshia N. Pipkin, Victoria J. Neal, Monica Toor, Brandi Gruber, Erin L. Wells, Kelly Iskiwitz, Carolyn J. Scatamacchia, Codi M. Anthony, Lisa S. Usdan, Lora J. McGill, Valerie K. Arnold	Clinical Neuroscience Solutions	Memphis, TN
Larkin Tyler Wadsworth III, MD	Horacio Marafioti, Lyly Dang, Lauren Clement, Kristen Johnson, Anya Penly, Elizabeth Garner, Angie Kean, Sophia Bolakas, Andrea Deffenbaugh, Cerece Miles, Lindsay Nooter, Christy Shultz, George Cherniawski, Stephanie Tesson, Ash Dale, Laura Hartupee, Breanna Galibert, Karen Knapp	Sundance Clinical Research	St. Louis, MO
Michael Waters, MD	Dalia Tover, Scott Overcash, Jordan Coslet, Michael Voskanian, Giuliano Zolin, Matthew Petro, Gina Weaver, Kia Lee, Hanh Chu, Karla Zepeda, Crystle Rajania, John Rodriguez, Tracey Fabrega, Kaitlyn Sandler, Alex Tapia, Cecilia Barbabosa, Renee Pasion, Jacob Pineda, Rosalynn Landazuri, Angelica Franco, Estee Garcia, Marilynn Rodriguez, Joanna Ocampo	Velocity Clinical Research	Chula Vista, CA
Jordan Whatley, MD	Jordan Whatley, Christopher Dedon, Emily Best, Amie Breaux Shannon, Mary Margaret Dobson, Nicole Harrell, Lindsey Kobetz Hall, Kristen LeBleu Losavio, Patricia Whatley, Tana Bourgeois, Alexandra Caillouet, Samantha Brooke McMillon, Amy Thomassie, Donna Michelle Hurst, Michelle Symms, Lyndsea Folsom, Crystal Rowell, Loney Girod, Lauren Sternfels, Makaylea Truitt, Lori Martin, April Mims	Meridian Clinical Research	Baton Rouge, LA
Jewel Johnny White, MD	Amanda Occhino, Ruth Paiano, Morgan McLaughlin, Elisa Swieboda	Synexus Clinical Research	The Villages, FL
Hayes Williams, MD, PhD	LaShondra Cade, Mitzi Roberts, Aileen Cunningham, Rhodna Fouts, Connie Moya, Gary Boyd, Justina Owens, Abby Wellinghurst	Achieve Clinical Research	Birmingham, AL
Clint Wilson, MD	Jason Milligan, Danielle Raley, Joseph Bocchini, Carrie Kay, Shannon Saksa, Courtney Harmon, Ashley Primos, CJ McKenna, Star Roberts	Willis-Knighton Health System / WKB Family Medicine Associates	Bossier City, LA
Peter J. Winkle, MD	Amina Z. Haggag, Elizabeth Lee, Michelle Haynes, Marysol Villegas, Sabina Raja, Mary Grace Lejarde, Caroline Villanueva, Natalie Ureno, Jessica Cramer, Steven Garcia, Yesenia Barraza, Ashley Barajas, Lauren Ferreira, Lucy Rems, Zaki Abawi, Damon Pineda, Patricio Ordonez, Gaby Huizar, Lesbia Alarcon, Anna Luz Belarmino, Nenita Llarena, Alberto Heshike, Isabel Rangel, Karen Cruz, Rynel Villanueva, Matthew Rohrig, Han Tran, Axl Dyer, Maria Webb, Akihisa Kodama, Cynthia Juarez, Sandra Gaona, Moriah Wilson, Mark Gonzalez	Anaheim Clinical Trials	Anaheim, CA
Patricia L. Winokur, MD	N/A	University of Iowa Medical Center	Iowa City, IA
Paul E. Wylie, MD	Renea Henderson, Natasa Jenson, Fan Yang, Amy Kelley, Kelly Knight, Jessica Watson, Stacy Tierney, Emily Knight, Jessica Woosley, Faith Fields, Glen Scott Thrower	Preferred Research Partners	Little Rock, AR
Carmen D. Zorrilla, MD	Carmen Irizarry, Gloria Martino, Natalia Muler, Lázaro Valdés	Universidad de Puerto Rico - Recinto de Ciencias Médicas - Maternal Infant Studies Center (CEMI)	San Juan, Puerto Rico

United States Government (USG)/Coronavirus Prevention Network (CoVPN) Biostatistics Team

Affiliation	Team Members
Biomedical Advanced Research and Development Authority (BARDA), Washington, DC	Di Lu, James Zhou
Department of Biostatistics and Bioinformatics, Rollins School of Public Health, Emory University	David Benkeser, Sohail Nizam
Vaccine and Infectious Disease Division, Fred Hutchinson Cancer Center, Seattle, WA	Jessica Andriesen, Bhavesh Borate, Lindsay N. Carpp, Andrew Fiore-Gartland, Youyi Fong*, Peter B. Gilbert*, Ying Huang*, Yunda Huang*, Ollivier Hyrien, Holly E. Janes*, Michal Juraska, Yiwen Lu, April K. Randhawa, Brian D. Williamson*, Lars W.P. van der Laan, Chenchen Yu
Biostatistics Research Branch, NIAID, NIH, Bethesda, MD	Michael P. Fay, Jonathan Fintzi, Dean Follmann, Martha Nason
Clinical Monitoring Research Program Directorate, Frederick National Laboratory for Cancer Research, Frederick, MD	Eric Chu
Department of Biostatistics, University of Washington, Seattle, WA	Marco Carone, James Peng, Charlotte Talham
Department of Statistics, University of Washington, Seattle, WA	Alex Luedtke
Department of Biostatistics, Harvard T.H. Chan School of Public Health, Boston, MA	Nima S. Hejazi
Department of Population Health Sciences, NYU Grossman School of Medicine, New York, New York	Iván Díaz
Department of Biostatistics and Bioinformatics, Duke University; Global Health Institute, Duke University	Avi Kenny

*YF, PBG, YiH, and HEJ are also affiliated with the Department of Biostatistics, University of Washington, Seattle, WA. PBG and YuH are also affiliated with the Public Health Sciences Division, Fred Hutchinson Cancer Research Center, Seattle, WA. YuH is also affiliated with the Department of Global Health, University of Washington, Seattle, WA. Brian D. Williamson is also affiliated with Kaiser Permanente Washington Health Research Institute, Seattle, Washington, USA.

Supplementary Methods

Exposure-proximal immune correlates of risk analysis

A hazards model was considered for time to event (Delta COVID-19):

$$\lambda(s) = \lambda_0(s) \exp (\beta_1 x (s - \tau) + \beta_2 W) I(\tau < s), \qquad (1)$$

where s is calendar time (number of days since start of the trial), τ is the number of days between the start of the trial and the peak time point, $x(t)$ is the true underlying antibody marker at time t post-peak (in a hypothetical set-up where the immunoassay was conducted on serum samples drawn on every day of follow-up), and W is the baseline covariate age group (≥65 vs <65). For each marker (nAb-ID50 Delta, nAb-ID50 D614G, Spike IgG Delta, and Spike IgG D614), a linear mixed effects model was used to model the log10 antibody marker trajectory over log(days post-peak +35), with fixed effect for log(days post-peak +35), age, sex, and random intercept for individuals, adjusting for the case–cohort sampling weights. Supplementary Figure 9 shows the longitudinal case-control antibody marker data that were used to fit the linear mixed effects model.

Based on the linear mixed effects model fit, the expected value of the immune marker at each day post-peak was estimated conditional on age, sex, and observed history of antibody marker measurements. Cox model parameters were estimated by maximizing the partial likelihood based on the induced hazard [1]. Delta COVID-19 hazard ratio (over LOD/2 or LLOQ/2) curves given the current value of the antibody marker x (i.e., HR with 10^{x-c} fold increase in antibody marker level over c=log10(LOD/2) or log10(LLOQ/2)),

$$\text{HR}(x) = \exp(\beta_1 (x-c)),$$

were then estimated based on the β estimates. The nonparametric bootstrap with 500 samples was used to construct 95% pointwise CIs for HR(x).

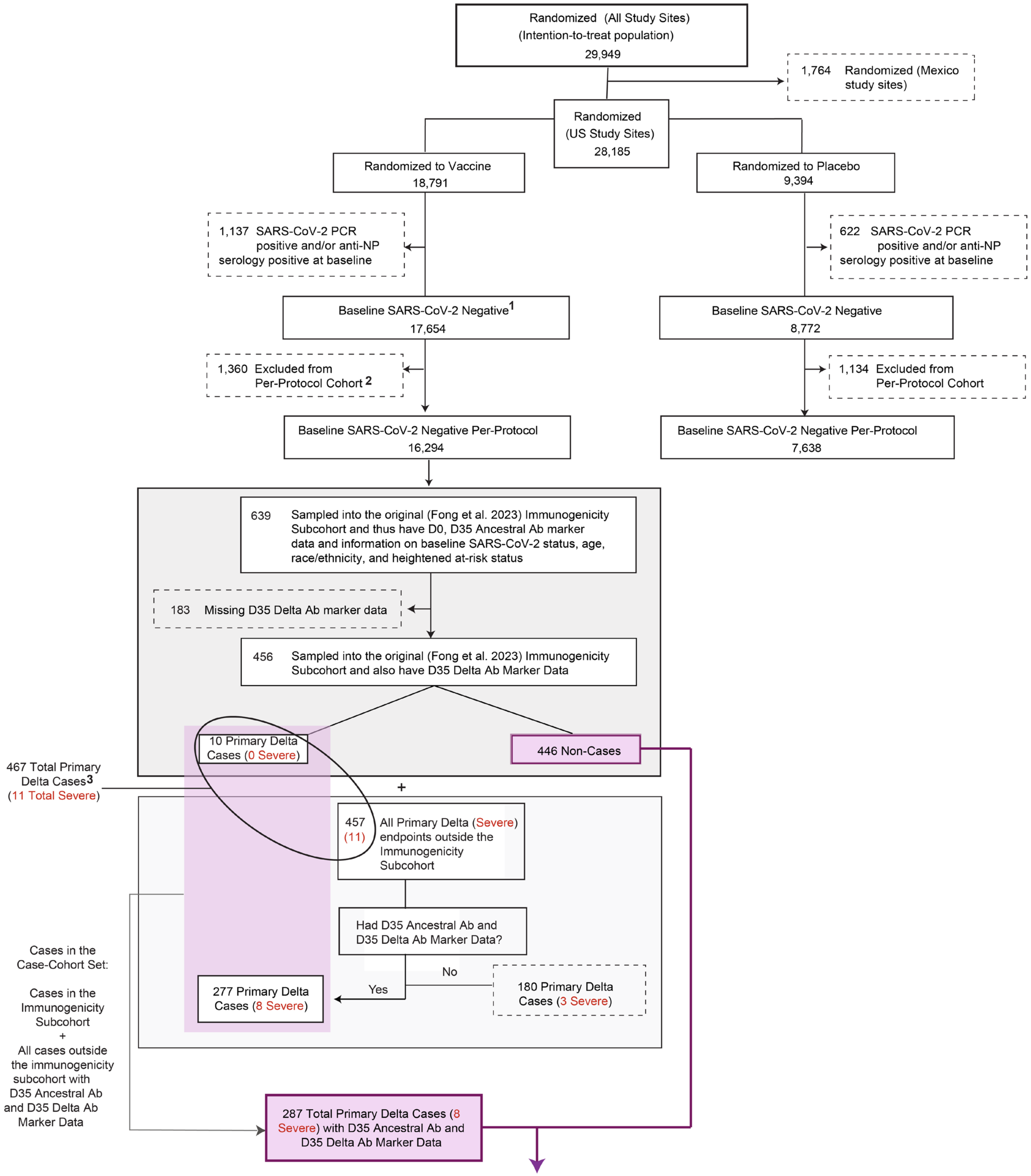

United States Study Sites Case-Cohort Set:
Used for immunogenicity analyses, D35 correlates analyses, and exposure-proximal correlates analyses[4]

Baseline demographic covariate strata

	**1**	**2**	**3**	**4**	**5**	**6**	**7**	**8**	**Total**
Minority[5]	N	N	N	N	Y	Y	Y	Y	
Age	18-64	18-64	≥65	≥65	18-64	18-64	≥65	≥65	
Co-existing conditions[6]	N	Y	N	Y	N	Y	N	Y	
Primary Delta (Severe) Cases	89 (0)	101 (4)	10 (0)	14 (2)	29 (0)	40 (2)	3 (0)	1 (0)	**287 (8)**
Non-Cases	76	56	74	65	47	49	34	45	**446**

1 Participants with missing baseline SARS-CoV-2 RT-PCR or anti-NP (nucleocapsid protein) serology data were considered baseline negative for the corresponding assay. After this, "baseline SARS-CoV-2 negative" was defined as baseline SARS-CoV-2 RT-PCR negative **and** baseline anti-NP serology negative.

2 Reasons for exclusion from per-protocol included: Did not receive two doses, doses out of allowed window, major protocol deviation.

3 Of the 467 total primary Delta cases, 332 were known Delta and 135 were imputed Delta.

4 Estimation of the linear mixed effects model for exposure-proximal immune correlates analyses used the subset of this set with antibody data at at least one longitudinal time point past D35.

5 Minority includes Blacks or African Americans, Hispanics or Latinos, American Indians or Alaska Natives, Native Hawaiians, and other Pacific Islanders. Non-Minority includes all other races with observed race (Asian, Multiracial, White, Other) and observed ethnicity Not Hispanic or Latino. Therefore Unknown and Not reported have missing values for this.

6 Co-existing conditions are the same as those listed in Table 1 of Dunkle et al. NEJM 2022: obesity (defined as a body-mass index [the weight in kilograms divided by the square of the height in meters] of ≥30.0), chronic lung disease, diabetes mellitus type 2, cardiovascular disease, or chronic kidney disease.

Supplementary Figure 1. Flowchart of study participants from randomization through membership into the per-protocol baseline SARS-CoV-2 negative case-cohort set (U.S. study sites) for this stage 2 correlates analysis. Membership in the case-cohort set required availability of D0 and D35 antibody data and no evidence of SARS-CoV-2 infection through 108 days post D35. Antibody data from the original placebo arm are not used in correlates analyses, given no variability in values; they were only used to verify low false positive rates of the immunoassays. "Original Fong et al. 2023" is reference [2].

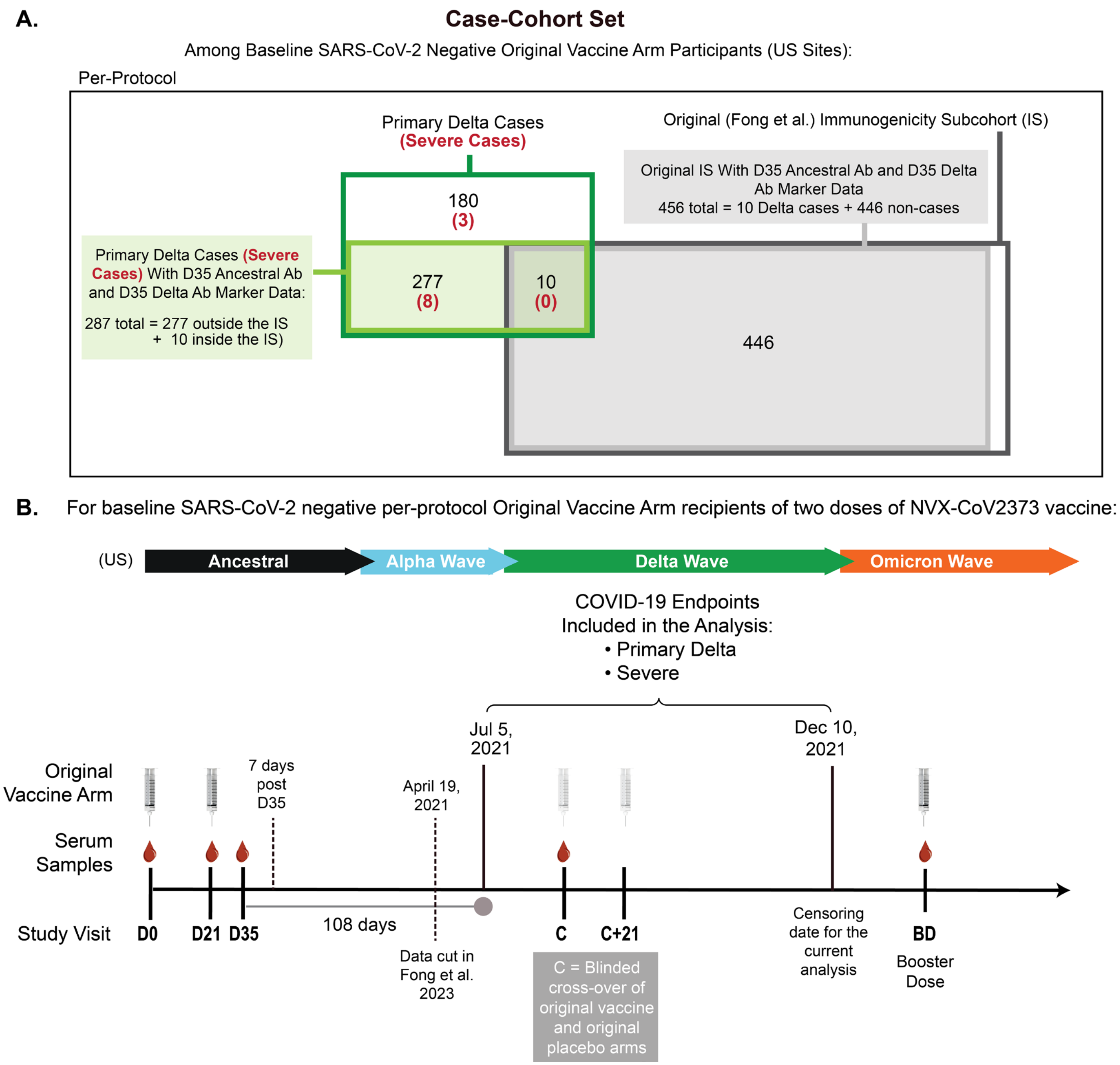

Supplementary Figure 2. A) Case-cohort set (restricting to US sites). Membership in the case-cohort set required availability of D0 and D35 antibody data and no evidence of SARS-CoV-2 infection through 108 days post D35. The numbers in parentheses are subsets of the numbers above them given that all severe COVID-19 cases are also Delta COVID-19 cases. Of the 467 total primary Delta cases shown, 332 were known Delta and 135 were imputed Delta. As all severe COVID-19 cases were also known or imputed Delta COVID-19 cases, the same antibody marker measurements were available for the severe COVID-19 cases. B) Timing of NVX-CoV2373 doses, serum sampling, and for counting primary Delta COVID-19 endpoints and severe COVID-19 endpoints included in the Day 35 marker correlates analysis. The darker-shaded syringes represent NVX-CoV2373 doses (given to the Original Vaccine Arm on D0 and D21, as well as on BD) and the lighter-shaded syringes represent placebo doses (given to the Original Vaccine Arm on C and C+21). In (A), cases are baseline SARS-CoV-2 negative per-protocol original

vaccine arm participants with the primary Delta COVID-19 endpoint (RT-PCR–confirmed symptomatic COVID-19 determined to be caused by the Delta variant through sequencing of SARS-CoV-2 genomes from nasal swabs [3] or via imputation) occurring after the data cut of Fong et al. [2] (April 19, 2021) and after 108 days post D35 through to December 10, 2021. “Baseline SARS-CoV-2 negative” and “per-protocol” are defined as in [3]: seronegative for anti-SARS-CoV-2 nucleoprotein and SARS-CoV-2 RNA RT-PCR-negative nasal swab at baseline; and received both planned vaccinations, had no specified protocol deviations, and were SARS-CoV-2 negative on the D21 visit, respectively. Severe COVID-19 endpoints were as described in [3].

A.

Geometric Mean Ratios of D35 Delta and Ancestral Markers

D35 Markers	Geometric Mean Ratio (95% CI)	p-value
nAb-ID50 Delta / nAb-ID50 D614G	0.329 (0.314, 0.344)	<0.001
Spike IgG Delta / Spike IgG D614	0.452 (0.442, 0.463)	<0.001

B.

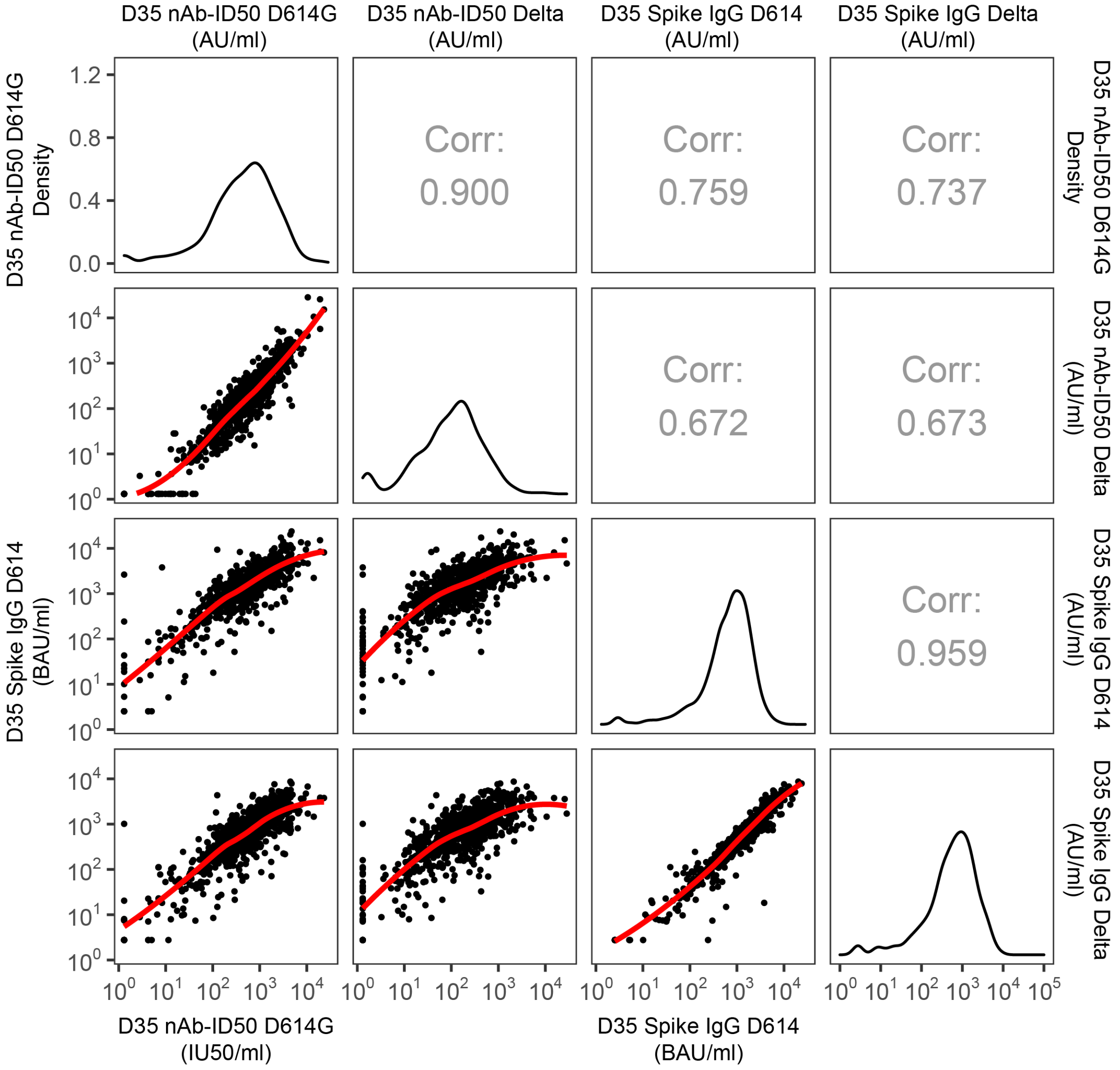

Supplementary Figure 3. A) Geometric mean ratios for D35 Delta vs. ancestral markers. P-values are from the paired *t*-test. B) Scatterplots of pairs of D35 Delta and ancestral antibody marker levels. Both panels analyzed data from US per-protocol baseline SARS-CoV-2 negative original vaccine arm recipients with no evidence of SARS-CoV-2 infection through 108 days post-D35. AU = arbitrary units/ml; Corr = Weighted Spearman correlation coefficient.

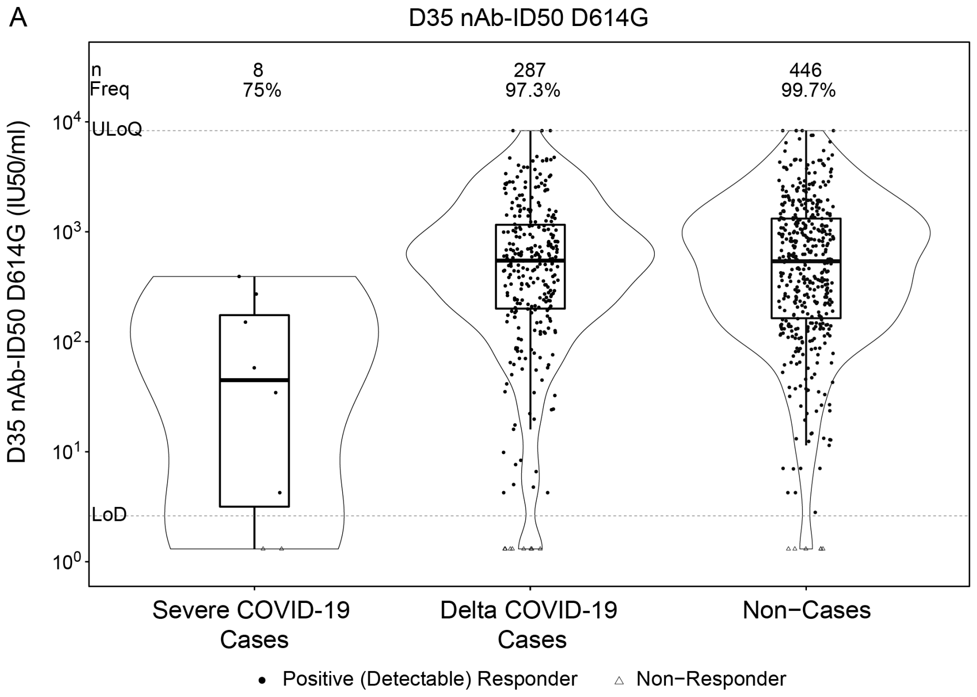

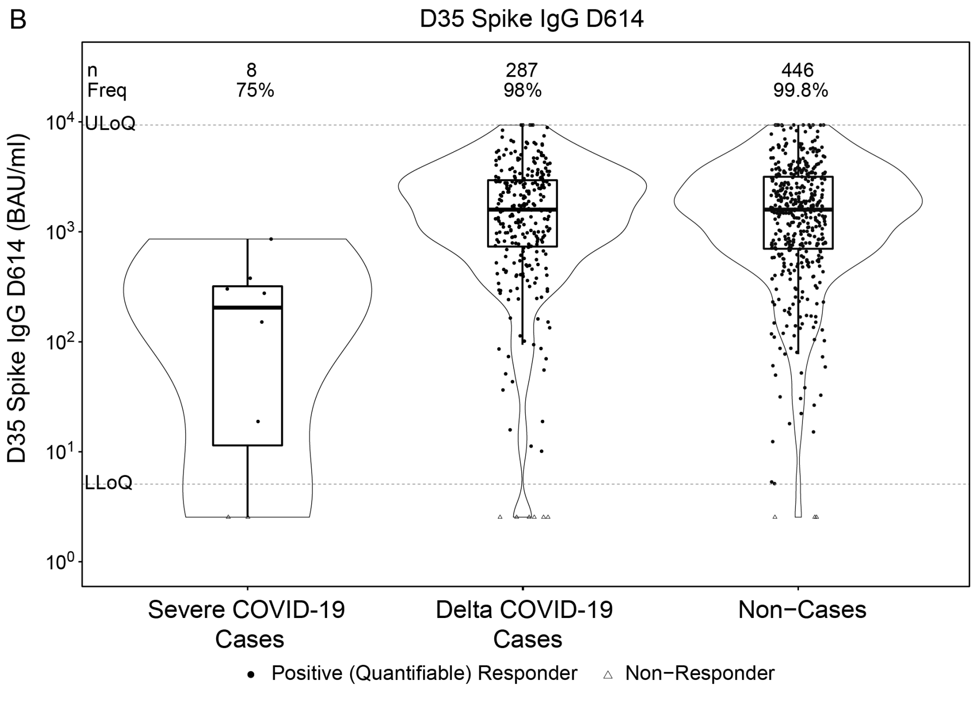

Supplementary Figure 4. Violin plots showing D35 levels of (A) 50% inhibitory dilution D614G-neutralizing antibody titer (nAb-ID50 D614G) in IU50/mL; (B) Anti-D614 Spike IgG concentration (Spike IgG D614) in BAU)/mL, shown separately for severe COVID-19 cases, Delta COVID-19 cases, and non-cases. Analysis based on US per-protocol baseline SARS-CoV-2–negative original vaccine arm participants with no evidence of SARS-CoV-2 infection through 108 days post-D35. The violin plots contain interior box plots with upper and lower horizontal edges the 25th and 75th percentiles of antibody level and middle line the 50th percentile, and vertical bars the distance from the 25th (or 75th) percentile

of antibody level and the minimum (or maximum) antibody level within the 25th (or 75th) percentile of antibody level minus (or plus) 1.5 times the interquartile range. At both sides of the box, a rotated probability density curve estimated by a kernel density estimator with a default Gaussian kernel is plotted. Numbers of participants (n) in each group with antibody data are reported at the top of the plots. Filled circles are positive (detectable) nAb-ID50 D614G or positive (quantifiable) Spike IgG D614 responses; open triangles are non-responders. Frequencies of participants with detectable nAb-ID50 D614G or quantifiable Spike IgG D614 responses were computed with inverse probability of sampling weighting and are also reported at the top of the plots as “Freq”. Positive (detectable) D35 nAb-ID50 D614G response was defined as D35 nAb-ID50 D614G ≥ LOD (2.612 IU50/mL). ULoQ = 8319.938 IU50/mL for nAb-ID50 D614G. Positive (quantifiable) D35 Spike IgG D614 was defined as D35 Spike IgG D614 ≥ LLOQ (5.0742 BAU/mL). ULoQ = 9370.615 BAU/mL for Spike IgG D614. Cases were in the original vaccine arm and experienced the severe COVID-19 endpoint and/or Delta COVID-19 endpoint, as applicable, after both April 19, 2021 and 108 days post-D35 through to December 10, 2021. Non-cases were defined as per-protocol baseline SARS-CoV-2 negative original vaccine arm participants randomly sampled from the Fong et al. immunogenicity subcohort with available D35 ancestral and D35 Delta antibody data with no evidence of SARS-CoV-2 infection (i.e., never tested RT-PCR positive) through December 10, 2021. BAU, binding antibody units; IU, international units; LLOQ, lower limit of quantitation; LOD, limit of detection; ULOQ, upper limit of quantitation.

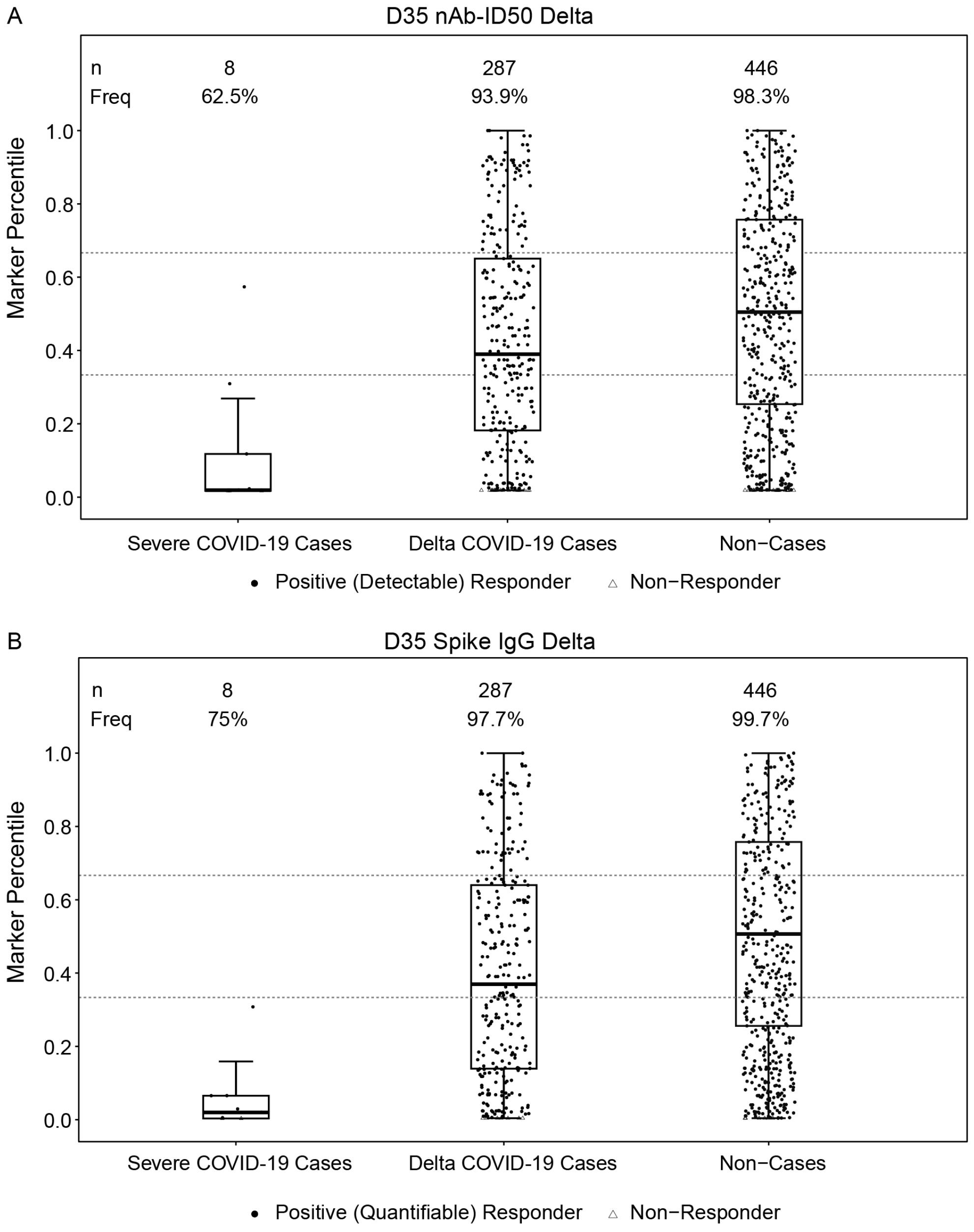

Supplementary Figure 5. Box plots showing marker percentiles of D35 A) 50% inhibitory dilution Delta-neutralizing antibody titer (nAb-ID50 Delta) in AU/ml and B) Anti-Delta Spike IgG concentration (Spike IgG Delta) in AU/ml, shown separately for severe COVID-19 cases, Delta COVID-19 cases, and non-cases. Analysis based on US per-protocol baseline SARS-CoV-2–negative original vaccine arm participants with no evidence of SARS-CoV-2 infection through 108 days post-D35. The two gray horizontal lines are at the 33rd (lower) and 67th (upper) percentiles of the D35 marker, where percentiles were calculated for the population for which inferences about correlates are drawn using inverse sampling probability weights. The box plots have upper and lower horizontal edges the 25th and 75th percentiles of antibody level and middle line the 50th percentile, and vertical bars the distance from the 25th (or 75th) percentile of antibody level and the minimum (or maximum) antibody level within the 25th (or 75th) percentile of antibody level minus (or plus) 1.5 times the interquartile range, and were calculated using inverse probability of sampling weighting. Numbers of participants (n) in each group with antibody data

are reported at the top of the plots. Filled circles are positive (detectable) nAb-ID50 Delta or positive (quantifiable) Spike IgG Delta responses; open triangles are non-responders. Frequencies of participants with positive (detectable) nAb-ID50 Delta or positive (quantifiable) Spike IgG Delta responses were computed with inverse probability of sampling weighting and are also reported at the top of the plots as "Freq". Positive (detectable) D35 nAb-ID50 Delta response was defined as D35 nAb-ID50 Delta ≥ LOD (2.612 AU/ml). ULoQ = 9524.53 AU/ml for nAb-ID50 Delta. Positive (quantifiable) D35 Spike IgG Delta was defined as D35 Spike IgG Delta ≥ LLOQ (5.5278 AU/ml). ULoQ = 6900.02 AU/ml for Spike IgG Delta. Cases were in the original vaccine arm and experienced the severe COVID-19 endpoint and/or Delta COVID-19 endpoint, as applicable, after both April 19, 2021 and 108 days post D35 through to December 10, 2021. Non-cases were defined as per-protocol baseline SARS-CoV-2 negative original vaccine arm participants randomly sampled from the Fong et al. immunogenicity subcohort with no evidence of SARS-CoV-2 infection (i.e., never tested RT-PCR positive) through December 10, 2021. AU, arbitrary units; LLOQ, lower limit of quantitation; LOD, limit of detection; ULOQ, upper limit of quantitation.

A

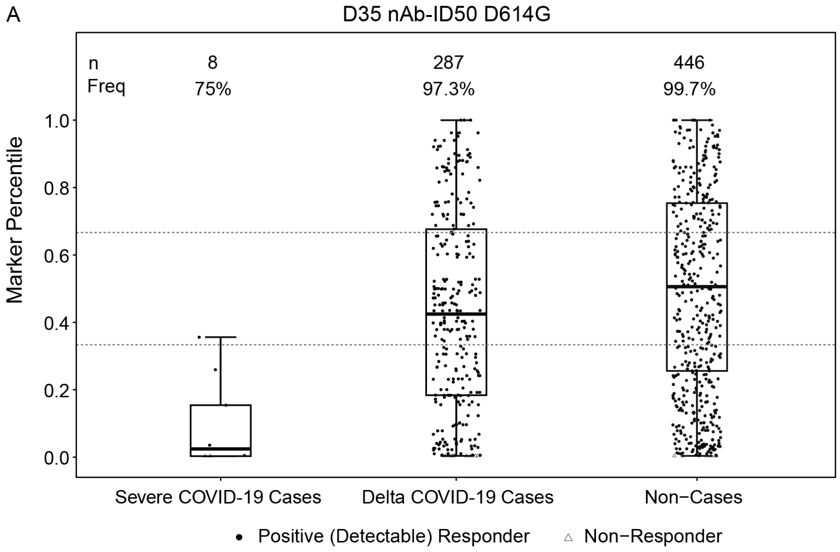

B

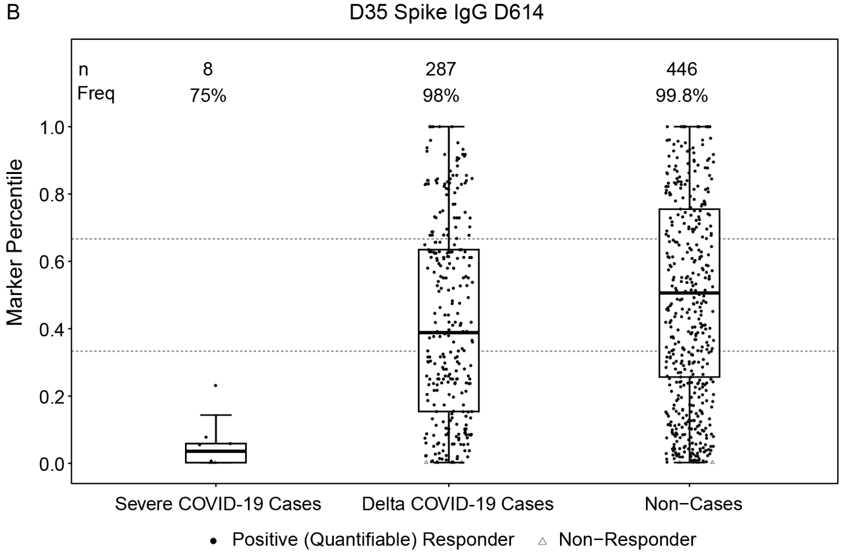

Supplementary Figure 6. Box plots showing marker percentiles of D35 A) 50% inhibitory dilution D614G-neutralizing antibody titer (nAb-ID50 D614G) in IU50/ml and B) Anti-D614 Spike IgG concentration (Spike IgG D614) in BAU)/ml, shown separately for severe COVID-19 cases, Delta COVID-19 cases, and non-cases. Analysis based on US per-protocol baseline SARS-CoV-2–negative original vaccine arm participants with no evidence of SARS-CoV-2 infection through 108 days post-D35. The two gray horizontal lines are at the 33rd (lower) and 67th (upper) percentiles of the D35 marker, where percentiles were calculated for the population for which inferences about correlates are drawn using inverse sampling probability weights. The interior box plots have upper and lower horizontal edges the 25th and 75th percentiles of antibody level and middle line the 50th percentile, and vertical bars the distance from the 25th (or 75th) percentile of antibody level and the minimum (or maximum) antibody level within the 25th (or 75th) percentile of antibody level minus (or plus) 1.5 times the interquartile range, and were calculated using inverse probability of sampling weighting. Numbers of participants (n) in each group with antibody data are reported at the top of the plots. Filled circles are positive (detectable) nAb-ID50 D614G or positive (quantifiable) Spike IgG D614 responses; open triangles are non-responders. Frequencies of participants with positive (detectable) nAb-ID50 D614G or positive (quantifiable) Spike IgG D614 responses were computed with inverse probability of sampling weighting and are also reported

at the top of the plots as “Freq”. Positive (detectable) D35 nAb-ID50 D614G response was defined as D35 nAb-ID50 D614G ≥ LOD (2.612 IU50/ml). ULoQ = 8319.938 IU50/ml for nAb-ID50 D614G. Positive (quantifiable) D35 Spike IgG D614 was defined as D35 Spike IgG D614 ≥ LLOQ (5.0742 BAU/ml). ULoQ = 9370.615 BAU/ml for Spike IgG D614. Cases were in the original vaccine arm and experienced the severe COVID-19 endpoint and/or Delta COVID-19 endpoint, as applicable, after both April 19, 2021 and 108 days post D35 through to December 10, 2021. Non-cases were defined as per-protocol baseline SARS-CoV-2 negative original vaccine arm participants randomly sampled from the Fong et al. immunogenicity subcohort with available D35 ancestral and D35 Delta antibody data with no evidence of SARS-CoV-2 infection (i.e., never tested RT-PCR positive) through December 10, 2021. BAU, binding antibody units; IU, international units; LLOQ, lower limit of quantitation; LOD, limit of detection; ULOQ, upper limit of quantitation.

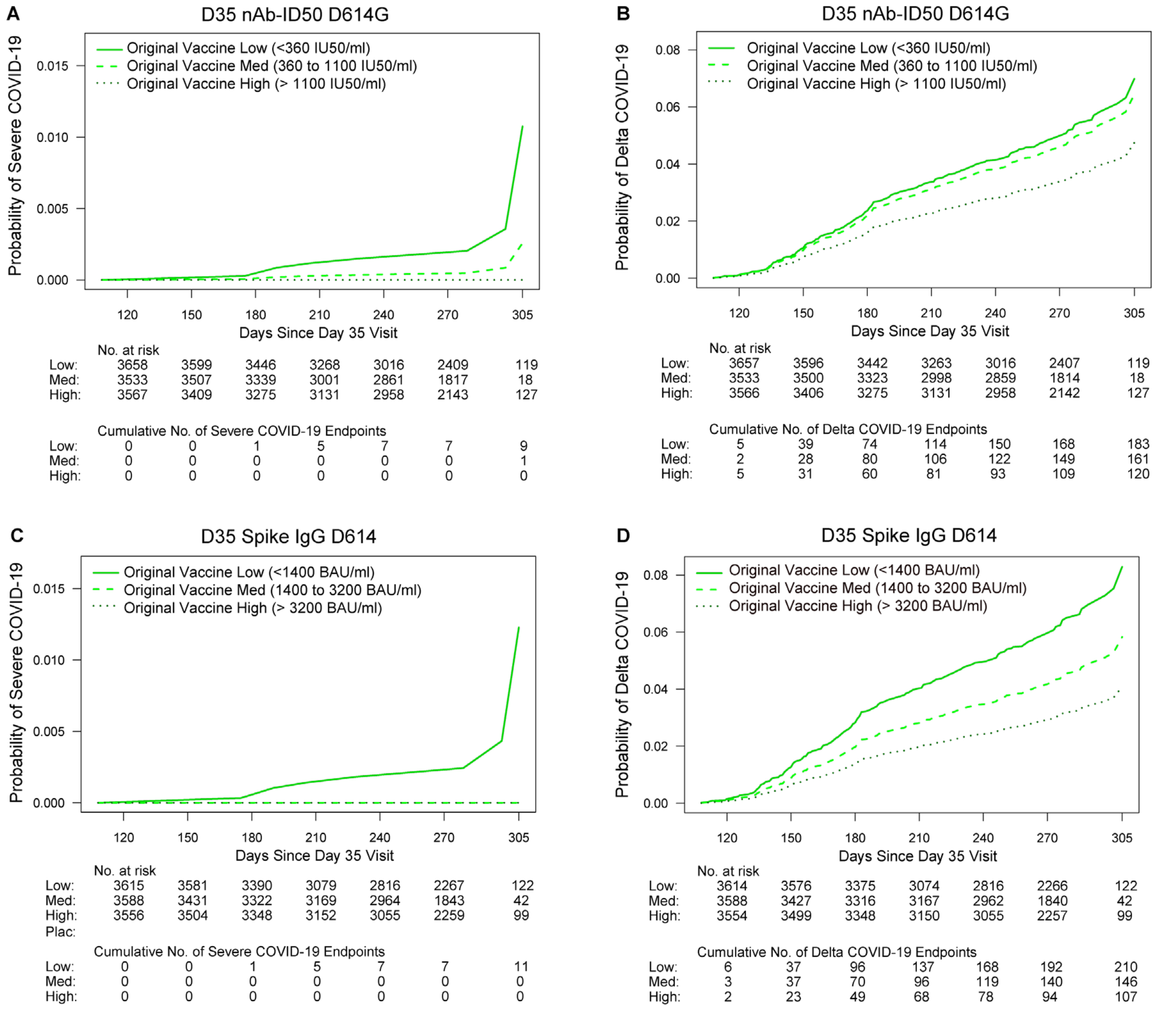

E

COVID-19 Endpoint	D35 Marker	Tertile	No. cases / No. at-risk*	Attack rate	Haz. Ratio Pt. Est.	Haz. Ratio 95% CI	P-value	Overall P-value
Severe	nAb-ID50 D614G	Low	9/3,662	0.0025	1	N/A	N/A	
		Medium	1/3,585	0.0003	0.24	(0.02, 2.87)	0.259	
		High	0/3,629	0.0000	0.00			
Severe	Spike IgG D614	Low	11/3,621	0.0030	1	N/A	N/A	
		Medium	0/3,641	0.0000	0.00			
		High	0/3,614	0.0000	0.00			
Delta	nAb-ID50 D614G	Low	183/3,662	0.0500	1	N/A	N/A	0.157
		Medium	161/3,585	0.0449	0.92	(0.61, 1.38)	0.685	
		High	120/3,629	0.0331	0.67	(0.44, 1.02)	0.063	
Delta	Spike IgG D614	Low	210/3,621	0.0580	1	N/A	N/A	0.004
		Medium	146/3,641	0.0401	0.69	(0.47, 1.03)	0.073	
		High	107/3,614	0.0296	0.48	(0.31, 0.74)	<0.001	

Supplementary Figure 7. Covariate-marginalized cumulative incidence of A, C) severe COVID-19 or B, D) Delta COVID-19 from 108 through 305 days post D35 by Low, Medium, High tertile of D35 ancestral antibody marker level: A, B) nAb-ID50 D614G; C, D) Spike IgG D614. Marker levels defining the Low, Medium, and High tertiles are shown in the upper left of each plot, with cut-off values determined as the 33rd and 67th percentiles of the D35 marker. Percentiles were calculated for the population for which inferences about correlates are drawn using inverse sampling probability weights. E) Estimated hazard ratios of severe COVID-19 or of Delta COVID-19 for the Medium vs. Low and for the High vs. Low

tertiles of D35 nAb-ID50 Delta or of D35 Spike IgG Delta. For tertiles with zero severe cases (High nAb-ID50 D614G, Medium Spike IgG D614, High Spike IgG D614), the Cox modeling cannot provide inferences involving comparison of that tertile to another tertile. The overall P-value is from a generalized Wald-test p-value of the null hypothesis that the hazard rate is constant across the Low, Medium, and High tertile groups. Analyses were based on US per-protocol baseline SARS-CoV-2 negative original vaccine arm participants with no evidence of SARS-CoV-2 infection through 108 days post D35. Analyses for severe COVID-19 adjusted for age, while analyses for Delta COVID-19 adjusted for age group ($\geq$ 65 vs. $<$ 65). *No. at-risk = estimated number in the population for analysis, i.e., US per-protocol baseline negative participants in the original vaccine arm with no evidence of SARS-CoV-2 infection through 108 days post D35. Case counting started 108 days post Day 35. The total case counts across tertiles are larger than those in Supplementary Figure 4 and Table 1 because the cases shown here do not require availability of D35 antibody data. nAb-ID50, 50% inhibitory dilution neutralizing antibody.

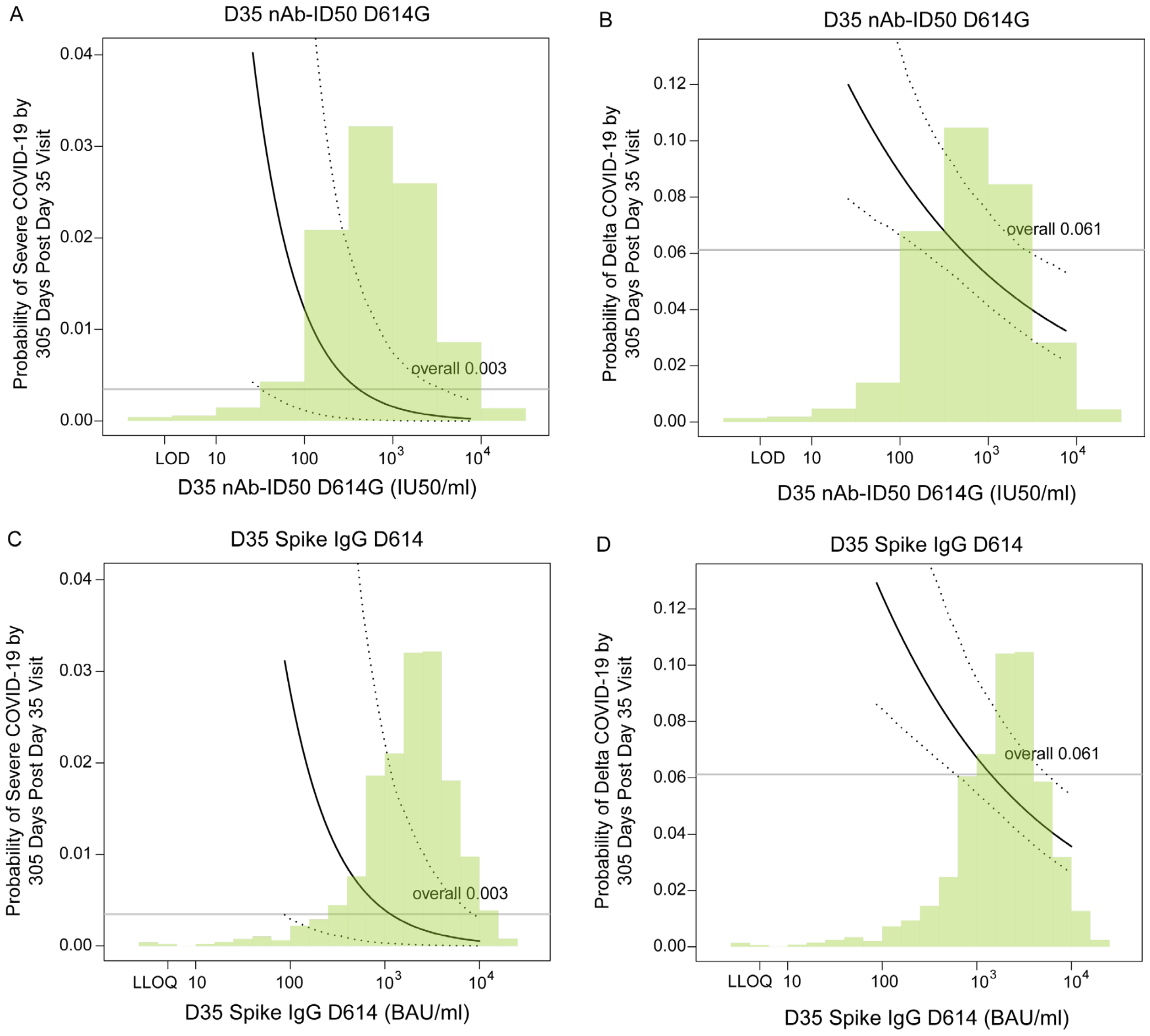

Supplementary Figure 8. Covariate-marginalized cumulative incidence of severe COVID-19 and of Delta COVID-19 from 108 through 305 days post D35 by D35 ancestral antibody level, estimated using a marginalized Cox model. Plots are shown for A, C) severe COVID-19 or B, D) Delta COVID-19, by A, B) nAb-ID50 D614G titer or C, D) anti-Spike IgG D614 concentration. The dotted black lines indicate bootstrap pointwise 95% CIs. The horizontal gray line is the overall cumulative incidence of severe COVID-19 (or Delta COVID-19, as relevant) from 108 to 305 days post D35 in the original vaccine arm. Curves are plotted over the antibody marker range from the 2.5th percentile to the 97.5th percentile: 26 to 7538 AU/ml for nAb-ID50 D614G, 87.3 to 10011 AU/ml for Spike IgG D614. Analyses were based on US per-protocol baseline SARS-CoV-2–negative original vaccine arm participants with no evidence of SARS-CoV-2 infection through 108 days post-D35. Analyses for severe COVID-19 adjusted for age in years, while analyses for Delta COVID-19 adjusted for age group (≥ 65 vs. < 65).

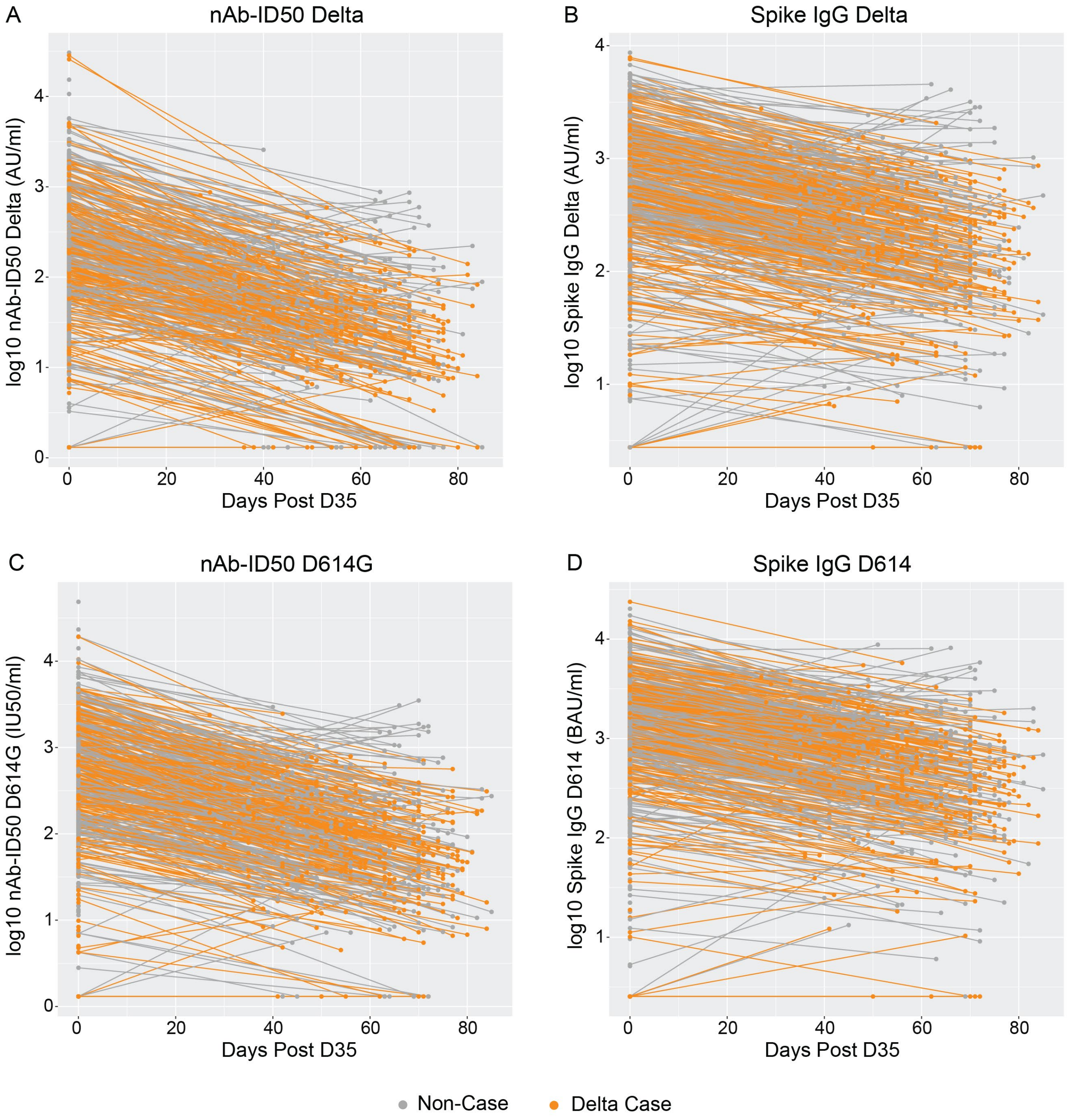

Supplementary Figure 9. Individual-level trajectories over time of A) nAb-ID50 Delta titers, B) anti-Spike Delta IgG concentrations, C) nAb-ID50 D614G titers, and D) anti-Spike Index concentrations in non-cases (gray) and Delta COVID-19 cases (orange). These trajectories (from N=495 non-cases and N=310 Delta cases) were used to fit a linear mixed effects model for each immune marker that was used to estimate its current value at each day post-D35 through to December 10, 2021, used in the exposure-proximal immune correlates analysis. Data are from the subset of the case-cohort set (see Supplementary Figure 1) with at least one longitudinal antibody measurement after D35, i.e. a measurement at the crossover visit ("C" in Supplementary Figure 2B). The days on the x-axis are calendar days since baseline; the D35 measurements do not align vertically due to variability in visit windows. C1 measurements do not align vertically due to variability in crossover implementation.

Supplementary Table 1. Sample sizes of per-protocol baseline SARS-CoV-2 negative original vaccine arm recipients with no evidence of SARS-CoV-2 infection through 108 days post-D35 included in the case-cohort set included in immune correlates analyses (US study sites), by A) baseline sampling strata and non-case/Delta case strata, and B) Hispanic or Latino vs. Not Hispanic or Latino x additional covariate strata and non-case/Delta case strata. Part B of the table is shown due to the finding that Hispanic or Latino participants were the only demographic group in which vaccine efficacy in the primary analysis was lower than in other subgroups [Figure 3 in Dunkle et al. [3]].

Case-cohort set = Per-protocol baseline SARS-CoV-2 negative original vaccine arm participants included in D35 marker correlates analysis [in the immunogenicity subcohort (IS) and/or a breakthrough Delta COVID-19 case)]. Membership in the case-cohort set required availability of D0 and D35 antibody data and no evidence of SARS-CoV-2 infection through 108 days post D35.

A. Baseline Sampling Strata of Baseline SARS-CoV-2 Negative Per-Protocol Original Vaccine Arm Participants With D35 Antibody Marker Data and Included in Correlates Analyses

	US<br>White Non-Hisp.<br>Age 18-64<br>Not at risk	US<br>White Non-Hisp.<br>Age 18-64<br>At risk	US<br>White Non-Hisp.<br>Age ≥ 65<br>Not at risk	US<br>White Non-Hisp.<br>Age ≥ 65<br>At risk	US<br>Minority<br>Age 18-64<br>Not at risk	US<br>Minority<br>Age 18-64<br>At risk	US<br>Minority<br>Age ≥ 65<br>Not at risk	US<br>Minority<br>Age ≥ 65<br>At risk	Total
Non-cases in the IS	76	56	74	65	47	49	34	45	446
Breakthrough Delta COVID-19 cases*	89	101	10	14	29	40	3	1	287

B. Additional Sampling Strata Presentation of Baseline SARS-CoV-2 Negative Per-Protocol Original Vaccine Arm Participants With D35 Antibody Marker Data and Included in Correlates Analyses

	US<br>Not Hisp. or Latino<br>Age 18-64<br>Not at risk	US<br>Not Hisp. or Latino<br>Age 18-64<br>At risk	US<br>Not Hisp. or Latino<br>Age ≥ 65<br>Not at risk	US<br>Not Hisp. or Latino<br>Age ≥ 65<br>At risk	US<br>Hisp. or Latino<br>Age 18-64<br>Not at risk	US<br>Hisp. or Latino<br>Age 18-64<br>At risk	US<br>Hisp. or Latino<br>Age ≥ 65<br>Not at risk	US<br>Hisp. or Latino<br>Age ≥ 65<br>At risk	Total
Non-cases in the IS	90	79	92	90	33	26	16	20	446
Breakthrough Delta COVID-19 cases*	102	114	11	15	16	27	2	0	287

Ab, antibody; IS, immunogenicity subcohort; White Non-Hisp., White Non-Hispanic.
*Cases include both those within and outside the IS.
Delta COVID-19 cases are per-protocol baseline SARS-CoV-2 negative original vaccine arm participants with the primary Delta COVID-19 endpoint (RT-PCR–confirmed symptomatic COVID-19 determined to be caused by the Delta variant through sequencing of SARS-CoV-2 genomes from nasal swabs or via imputation) occurring after the data cut of Fong et al. [2] (April 19, 2021) and after 108 days post D35 through to December 10, 2021. "Baseline SARS-CoV-2 negative" and "per-protocol" are defined as in [3]: seronegative for anti–SARS-CoV-2 nucleoprotein at baseline (had either a negative result or missing data) and had a SARS-CoV-2 RNA RT-PCR-negative nasal swab at baseline (had either a negative result or missing data); and received both planned vaccinations, had no specified protocol deviations, and were SARS-CoV-2 negative on the D21 visit, respectively.

For the covariate strata:
White Non-Hispanic is defined as Race=White and Ethnicity=Not Hispanic or Latino. All other Race subgroups are defined as Black, Asian, American Indian or Alaska Native, Native Hawaiian or Other Pacific Islander, Multiracial, Other, Not reported, or Unknown.
Minority is defined as the complement of being known to be White Non-Hispanic.
"At-risk" refers to conditions refers to participants having coexisting conditions associated with high risk of severe COVID-19 illness, with co-existing conditions defined in [3] (obesity, chronic lung disease, diabetes mellitus type 2, cardiovascular disease, and/or chronic kidney disease).

Supplementary Table 2. Assay limits of the neutralizing antibody and binding antibody markers evaluated as immune correlates. AU = arbitrary units; IU = International Units; nAb-ID50, 50% inhibitory dilution neutralizing antibody titer; LLOQ, lower limit of quantitation; ULOQ, upper limit of quantitation.

**Marker**	**LLOQ**	**ULOQ**	**LOD**	**Positivity Cutoff**
nAb-ID50 D614G (AU/ml)*	3.3303	8319.94	2.612	2.612
nAb-ID50 Delta (AU/ml)	3.265	9524.53	2.612	2.612
Anti Spike IgG – D614 (AU/ml)*	5.0742	9370.62		5.0742
Anti Spike IgG - Delta (AU/ml)	5.5278	6900.02		5.5278

*For nAb-ID50 D614G and Anti Spike IgG D614, AU/ml = IU50/ml and AU/ml = BAU/ml, respectively. IU = International Units; BAU = binding antibody units.

Positive nAb-ID50 response was defined as nAb-ID50 value at the time point greater than or equal to the antigen-specific LLOQ; otherwise the response was not quantifiable.

Positive binding antibody response was defined as anti Spike IgG concentration greater than the antigen-specific positivity cutoff; otherwise the response was negative.

Supplementary Table 3. Demographic and clinical characteristics at 7 days post D35 in the baseline SARS-CoV-2 negative per-protocol immunogenicity subcohort (IS) for the present manuscript (US study sites), consisting of a subset of the stage 1 IS in Fong et al. [2]. A subset of this IS is used in the present correlates analysis because the correlates analysis population is defined at 180 days post D35. N = number of participants with D35 antibody data.

Characteristics	Original Vaccine Arm (N = 560)
**Age**	
Age < 65	292 (52.1%)
Age ≥ 65	268 (47.9%)
Mean (Range)	55.2 (18.0, 86.0)
**BMI**	
Underweight BMI < 18.5	10 (1.8%)
Normal 18.5 ≤ BMI < 25	143 (25.5%)
Overweight 25 ≤ BMI < 30	201 (35.9%)
Obese BMI ≥ 30	202 (36.1%)
**Risk for Severe COVID-19**	
At-risk	272 (48.6%)
Not at-risk	288 (51.4%)
**Age, Risk for Severe COVID-19**	
Age < 65 At-risk	138 (24.6%)
Age < 65 Not at-risk	154 (27.5%)
Age ≥ 65	268 (47.9%)
**Sex**	
Female	264 (47.1%)
Male	296 (52.9%)
**Hispanic or Latino Ethnicity**	
Hispanic or Latino	119 (21.2%)
Not Hispanic or Latino	436 (77.9%)
Not reported and unknown	5 (0.9%)
**Race**	
White	384 (68.6%)
Black or African American	98 (17.5%)
Asian	41 (7.3%)
American Indian or Alaska Native	14 (2.5%)
Native Hawaiian or Other Pacific Islander	1 (0.2%)
Multiracial	12 (2.1%)
Not reported and unknown	10 (1.8%)
**Underrepresented Minority Status in the U.S.**	
White Non-Hispanic	335 (59.8%)
Communities of Color	225 (40.2%)
**Country**	
United States	560 (100.0%)
**Living With HIV**	
No	556 (99.3%)
Yes	4 (0.7%)

"At-risk" refers to conditions refers to participants having coexisting conditions associated with high risk of severe COVID-19 illness, with co-existing conditions defined in Dunkle et al. [3] (obesity, chronic lung disease, diabetes mellitus type 2, cardiovascular disease, or chronic kidney disease).
White Non-Hispanic is defined as Race=White and Ethnicity=Not Hispanic or Latino. All other Race subgroups are defined as Black, Asian, American Indian or Alaska Native, Native Hawaiian or Other Pacific Islander, Multiracial, Other, Not reported, or Unknown. Minority is defined as the complement of being known to be White Non-Hispanic.

Supplementary References

1. Prentice RL. Covariate measurement errors and parameter estimation in a failure time regression model. Biometrika **1982**; 69:331-42.
2. Fong Y, Huang Y, Benkeser D, et al. Immune Correlates Analysis of the PREVENT-19 COVID-19 Vaccine Efficacy Clinical Trial Nature Communications **2023**; 14:331.
3. Dunkle LM, Kotloff KL, Gay CL, et al. Efficacy and Safety of NVX-CoV2373 in Adults in the United States and Mexico. N Engl J Med **2022**; 386:531-43.

Statistical Analysis Plan for Study of Post Dose 2 and Exposure-Proximal Delta Antibody as Immune Correlates for Delta and Severe COVID-19 in the PREVENT19 Study

USG COVID-19 Response Team / Coronavirus Prevention Network (CoVPN) Biostatistics Team

January 6, 2025

Contents

List of Tables 3

List of Figures 4

1 Outline 5

2 Stage 1 correlates sampling design 5

3 Objectives of Delta correlates study 5

4 Stage 2 sampling design for addressing the objectives 5

5 Unsupervised Exploratory Analyses 7
5.1 Tabular description of antibody marker data 8
5.2 Graphical description of antibody marker data 10

6 Correlates of Risk Analysis Plan 11
6.1 Descriptive statistics 11
6.2 Assessing Objective 1, 2 (≈peak Ab Correlates of Risk) 13
6.2.1 Covariates adjusted for in CoR and CoP analyses 13
6.3 Assessing Objectives 3 (Exposure-Proximal Correlates of Risk) 13

7 Specifications for general issues faced for most analyses 14
7.1 Imputation of demographics variables for stratification and merging of sparse strata for weights computation 14

8 Additional data analysis issues 14
8.1 Exclude participants reporting being HIV positive from the correlates analysis . . . 14
8.2 Missing lineages 14
8.3 End of followup and non-case definition 14
8.4 Definition of severe COVID-19 15

List of Tables

1 Baseline Subgroups that are Analyzed[1]. 10

List of Figures

1 Flow-chart of the stage 2 correlates study that evaluates Delta antibody as a correlates of risk and of protection of Delta COVID-19. 6

1 Outline

First, this document recapitulates the sampling design that was used for assessment of Stage 1 correlates (Fong et al., 2023). Second, it states the study objectives to assess post dose 2 Delta antibody titer, and exposure-proximal antibody titer, as immune correlates for Delta COVID-19. Third, it describes the sampling plan for enabling the immune correlates statistical analyses. Fourth, it specifies the statistical analysis plan that details how to assess each objective.

2 Stage 1 correlates sampling design

A two-phase stratified case-cohort sampling design was applied for measuring D0, 35 antibody levels after the two-dose primary series in per-protocol participants sampled into the immunogenicity subcohort and for all baseline negative per-protocol vaccine recipient COVID-19 endpoint cases occurring at least 7 days post D35 visit. The implemented sampling design is described in the Supplementary Material of Fong et al. (2023).

3 Objectives of Delta correlates study

The following objectives are assessed in per protocol, SAR-CoV-2 naïve, original vaccine arm individuals. The study endpoint for all objectives is adjudicated "Delta COVID-19" or severe COVID counted starting 7 days after the D35 visit and starting April 19, 2021 or later. "instantaneous Delta COVID-19" refers to the instantaneous hazard rate of Delta COVID-19, i.e., the rate of Delta COVID-19 over the next day of follow-up. The objectives are assessed primarily for Delta antibodies and ancestral strain antibodies.

Objectives

1. To assess D35 Delta and ancestral Ab as a correlate of risk (CoR) against severe COVID-19
2. To assess D35 Delta and ancestral Ab as a correlate of risk (CoR) against Delta COVID-19
3. To assess Delta Ab as an exposure-proximal CoR of instantaneous Delta COVID-19

Note that although sampling plan requires DD1 samples, the ph1 definition will not require that for the peak correlates.

Note that for Delta indicators, non-Delta COVID will be censored and competing risk analyses will be run.

Note that of all ptids with more than one COVID events, the first one is always Delta.

4 Stage 2 sampling design for addressing the objectives

Figure 1 shows the blood sampling schedule that enables the correlates studies. This correlates study is a stratified case-control study of post-dose 2 Delta Ab. The study population for inference in the Stage 2 correlates study is the same as for the Stage 1 correlates study: per-protocol baseline SARS-CoV-2 negative (PPBN) individuals, except that the Stage 2 correlates analysis was

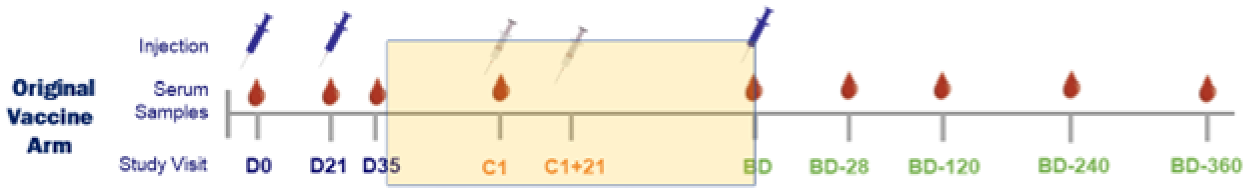

Figure 1: Flow-chart of the stage 2 correlates study that evaluates Delta antibody as a correlates of risk and of protection of Delta COVID-19.

performed in individuals with no evidence of SARS-CoV-2 infection through 108 days (vs. 6 days in the Stage 1 correlates analysis) post D35. The sampling approach samples Delta COVID-19 endpoint cases from the PPBN original vaccine arm.

The primary study endpoint is Delta COVID-19 occurring after the data cut April 19, 2021 [that was used for the Stage 1 correlates analysis of Fong et al. (2023)] and after 108 days post the D35 visit through to booster dose (BD) and the start of the Omicron wave 2021-12-10.

Among eligible non-cases, the Stage 2 sampling plan selects a stratified random sample of Original vaccine arm participants from the original immunogenicity subcohort analyzed in Fong et al. (2023), using balanced sampling across the 10 demographic strata the same as implemented previously. The sampling design prioritizes sampling participants with Day 0 and Day 35 data already available on antibody to the vaccine strain (D614 for binding antibody, D614G/WA-1 for pseudovirus neutralizing antibody); moreover, only participants with no evidence of SARS-CoV-2 infection (symptomatic COVID-19 or undiagnosed anti-NP/PCR positive infections) through to the data cut 2022-08-22, are eligible. The original immunogenicity subcohort is used because vaccine-strain directed antibody data are already available, which will enrich the correlates analyses. Only Original vaccine arm participants are included because blood samples were stored at 2 weeks post dose 2 from all study participants in this randomized study arm but were not stored at this time point from all study participants in the placebo arm, such that it generally would not be possible to measure peak Ab post dose 2 for placebo recipients who crossed over to the vaccine arm. In addition, to be eligible for sampling, non-cases must have a BD sample available, for aiding Objective 3. Because Objective 3 can be potentially assessed without this sample, the descriptive tables 2 and 3 below verify that there are enough eligible non-cases with a BD sample available to keep this requirement.

For the group PPBN Original vaccine arm recipient COVID-19 severe cases occurring at least 7 days post D35 through to the booster dose (BD) and occurring between the two data cut dates of April 19, 2021 and March 26, 2022, all such severe cases with lineage not known to be Delta are also selected for sampling. (Case severity is a preliminary call at the time of making these tables but will be finalized later.) This is done to maximize the number of severe breakthrough cases for analysis, not absolutely requiring Delta lineage, a factor that is required for the correlates analysis of the symptomatic COVID-19 endpoint.

Based on sample availability, the sampling plan is as follows:

- Delta Cases: Delta Ab are measured for all PPBN Original vaccine arm recipient COVID-19 Delta cases occurring at least 7 days post D35 through to the first booster dose (BD) and occurring between the two data cut dates of April 19, 2021 and March 26, 2022. Specifically, for all 366+36=402 Delta or severe cases with DD1 sample in the sampling design document Table 2, measure:
  - Delta Ab at D35, DD1, and C1, where Ab at C1 is measured only if C1 occurs before the COVID-19 event time and if the C1 sample is available. (In most cases C1 occur before even time. All C1 samples may be sent to the lab for ease of logistical implementation.)
  - WA-1 Ab at (D35, DD1, and C1), both for aiding interpretation of results and for studying whether vaccine-strain antibody provides a better or worse correlate for Delta COVID-19 than Delta antibody.
- Non-Cases: Delta Ab are measured from a demographic-stratified random sample of non-cases in the Original vaccine arm immunogenicity subcohort with no evidence of SARS-CoV-2 infection through March 26, 2022. Specifically:
  - For all 279 non-cases with both C1 and BD samples in the sampling design document Table 3, measure Delta Ab at D35, C1 and BD. (D35 samples are already at the lab, C1 and BD samples will be sent to the lab.)
  - For all 649 non-cases in the sampling design document Table 3, measure Delta Ab at D35. (D35 samples are already at the lab.)

In total, we expect to obtain antibody data from:

1. 1051 ptids = 366 + 36 + 649
2. 2413 samples = (366+36)*3 + 279*3 + (649 - 279)

Note that for the 366+36 Delta or Severe cases with DD1 sample in the sampling design document Table 2, both Delta and WA-1 Ab are to be measured at D35, DD1 and C1.

Of the 366 Delta cases, 33 are in the Senior age group, 15 are severe, and 2 are severe and Senior.

5 Unsupervised Exploratory Analyses

Two time points are of interest: D35 and C1. Interpretation of C1 immunogenicity is subject to the caveat that the median of interval between D35 and C1 is about 60 days. BD1 is not of interest because the median of interval between D35 and BD1 is about 330 days.

Only seronegative subjects within the vaccine arm are of interest. In terms of country, both US and Mex subjects are included, unlike the correlates analysis, which is restricted to the US.

Only subjects from the immunogenicity subcohort are included in this analysis. Within the subcohort, the number of ptids with C1 markers is a little less than half of the ptids with D35 markers. The weight used for D35 immunogenicity is called wt.immuno.D35 (ph1.immuno.D35, ph2.immuno.D35); similarly, the weight used for C1 immunogenicity is called wt.immuno.C1 (ph1.immuno.C1,

ph2.immuno.C1). Note that (i) ph1.immuno.C1 is set to be equal to ph1.immuno.D35, (ii) ph2.immuno.C1 requires ph2.immuno.D35. Hence, it is a subset of ph2.immuno.D35, and (iii) for C1, no imputation is done across markers so as not to interfere with exposure-proximal correlates analysis. Both wt.immuno.D35 and wt.immuno.C1 are only used for case outcome-status blinded immunogenicity inferential analyses and not for correlates analyses.

5.1 Tabular description of antibody marker data

Tables of immunogenicity will be reported separately by assay, which amounts to the following variables:

1. $\log_{10}$ nAb titer to D614G
2. $\log_{10}$ nAb titer to Delta (B16172)
3. $\log_{10}$ anti-Spike IgG to D614
4. $\log_{10}$ anti-Spike IgG to Delta
5. $\log_{10}$ anti-Spike IgG to alternate 1 Delta
6. $\log_{10}$ anti-Spike IgG to alternate 2 Delta
7. $\log_{10}$ anti-Spike IgG to Alpha
8. $\log_{10}$ anti-Spike IgG to Beta
9. $\log_{10}$ anti-Spike IgG to Gamma
10. $\log_{10}$ anti-Spike IgG to Omicron

Assay readouts accounting for assay limits (before multiplying the readouts by constants)

The readouts for the ancestral-specific nAb ID50 D614G titer and IgG Spike D614 concentration are converted from arbitrary units (AU/ml) to international units (IU50/ml and BAU/ml) using constant multiplicative conversion factors of 0.0653 for ID50 and 0.009 for IgG, respectively. The same conversions were used in Fong et al. (2023).

The AU/ml readouts for the Delta-specific nAb ID50 titer and IgG Spike D614 concentration are also multiplied by 0.0653 and 0.009, respectively, to be consistent with the ancestral-specific readouts, although their units remain AU/ml because international units do not exist for these Delta strain readouts.

The assay limits and labeling are recorded in the assay metadata file in the correlates2_reporting2 Github repo.

Definition of participants with a positive response

- Participants with a positive (detectable) pseudovirus neutralization response at each predefined timepoint are defined as participants who had ID50 value at the time point greater than or equal to the antigen-specific LOD; otherwise the response is not detectable. Values below the LOD are assigned value LOD/2.

- Participants with a positive (quantifiable) bAb response at each pre-defined timepoint are defined as participants who had IgG value at the time point greater than or equal to the antigen-specific LLOQ; otherwise the response is not quantifiable. Values below the antigen-specific LLOQ are assigned the value LLOQ/2.

The tables will include, for each pre-defined post-baseline time point:

1. For each antibody marker, the estimated percentage of participants defined as responders will be provided with the corresponding 95% CIs using the Clopper-Pearson method.
2. Geometric mean titers (GMTs) and geometric mean concentrations (GMCs) will be summarized along with their 95% CIs using the t-distribution approximation of log-transformed concentrations/titers
3. The differences in the responder rates, 2FRs, 4FRs between groups will be computed along with the two-sided 95% CIs by the Wilson-Score method without continuity correction (Newcombe, 1998) (the groups for comparison are as described in the previous bullet).

All of the above point and confidence interval estimates will use inverse probability of antibody marker sampling weighting in order that estimates and inferences are for the population from which the whole study cohort was drawn.

Tables will be provided separately for (1) baseline negative individuals, (2) baseline negative individuals by subgroup defined as in Table 1. Each table will show data for all available time points in the original vaccine arm.

Table 1: Baseline Subgroups that are Analyzed[1].

**Age:** 18-64, ≥ 65
**Coexisting conditions:** Yes, No
18-64 Coex. cond., 18-64 No coex. cond., ≥ 65 Coex. cond., ≥ 65 No coex. cond.
**Sex:** Male, Female
**Age x Sex:**
18-64 Male, 18-64 Female, ≥ 65 Female, ≥ 65 Male
**Hispanic or Latino Ethnicity:** Hispanic or Latino, Not Hispanic or Latino
**Race or Ethnic Group:**
White Non-Hispanic[2], Black, Asian, American Indian or Alaska Native (NatAmer)
Native Hawaiian or Other Pacific Islander (PacIsl), Multiracial,
Other, Not reported, Unknown
**Underrepresented Minority Status in the U.S.:**
Communities of color (Comm. of color), White[2]
**Age x Underrepresented Minority Status in the U.S.:**
Age ≥ 65 Comm. of color, Age < 65 Comm. of color, Age ≥ 65 White, Age ≥ 65 White

[2]White Non-Hispanic is defined as Race=White and Ethnicity=Not Hispanic or Latino. All of the other Race subgroups are defined solely by the Race variable, with levels Black, Asian, American Indian or Alaska Native, Native Hawaiian or Other Pacific Islander, Multiracial, Other, Not reported, Unknown. Communities of color is defined by the complement of being known White Non-Hispanic.

For comparing antibody levels between groups, the following groups are compared:

- Within baseline negative vaccine recipients, compare each of the following pairs of subgroups listed in Table 1: Age ≥ 65 vs. age < 65; risk for severe COVID: at risk vs. not at risk; age ≥ 65 at risk vs. age ≥ 65 not at risk; age < 65 at risk vs. age < 65 not at risk; male vs. female; Hispanic or Latino ethnicity: Hispanic or Latino vs. Not Hispanic or Latino; Underrepresented minority status: Communities of color vs. White Non-Hispanic (within the U.S.).

5.2 Graphical description of antibody marker data

The Day 35 antibody marker data collected from the immunogenicity subcohort participants will be described graphically. These data are representative of the entire study cohort. Importantly, only antibody data from the immunogenicity subcohort are included (i.e., no data from cases outside the subcohort are included). This makes the analyses unsupervised (independent of case-control status), enabling interrogation and optimization of the antibody biomarkers prior to the inferential correlates analyses.

Plots are developed for the following purposes. All of the analyses are only done within baseline negative vaccine recipients. In addition, many of the descriptive analyses will also be done separately for each demographic subgroup of interest listed above. For descriptive plots of individual marker

data points that pool over one or more of the baseline strata subgroups, plots show all observed data points.

Three sets of ph2 indicators and weights are used: ph2.immuno.D35/wt.immuno.D35, ph2.immuno.C1/wt.immuno.C1, and ph2.immuno.BD1/wt.immuno.BD1. ph2.immuno.C1 is nested within ph2.immuno.D35 and ph2.immuno.BD1 is nested within ph2.immuno.C1. For analyses using more than one time point, either wt.immuno.C1 or wt.immuno.BD1 is used.

The following descriptive graphical analyses are done. All readouts will be plotted on the $\log_{10}$ scale, with plotting labels on the natural scale.

1. The distribution of each antibody marker readout at Day 35 and C1 will be described with plots of empirical reverse cumulative distribution functions (rcdfs) and boxplots (including individual data points) . Inverse probability of sampling into the subcohort weights are used in the estimation of the rcdf curves; henceforth we refer to these weights as "inverse probability of sampling" (IPS) weights.

2. The correlation of each antibody marker readout between D35 and C1, is examined. Pairs plots/scatterplots will be used, annotated with inverse probability-weighted Spearman rank correlations.

3. The correlation of each pair of Day 35 antibody marker readouts are compared. Pairs plots/scatterplots and inverse probability-weighted Spearman rank correlations are used, with inverse probability-weighted Spearman rank correlations computed as described above. The same analyses are done for each pair of C1 antibody marker readouts.

4. Point estimates of D35 and C1 marker positive response rates. The point and 95% CI estimates include all of the data and use IPS weights.

6 Correlates of Risk Analysis Plan

6.1 Descriptive statistics

In constrast with the immuno reports, inverse-probability weighting will use wt.D35_108. Only 4 of the 10 markers will be studied to focus on ancestral and Delta-specific antibody markers: pseudoneutid50_D614G, pseudoneutid50_Delta, bindSpike_D614, and bindSpike_Delta1.

Tabular output

For each marker:

- Number (%) positive responses (including denominator that is the estimated number of participants in the population in the cell) with 95% CI at each time point (columns) by Delta case, severe case, or non-case status (rows). 95% CI calculated based on Clopper-Pearson method. Table pools participants over the baseline strata. The time points are D35/C1/DD1 for cases and D35/C1/BD for non-cases.

- Geometric mean (95% CI) of quantitative marker at each time point (columns) by Delta case, severe case, or non-case status (rows). 95% CIs using the t-distribution approximation

of $\log_{10}$-transformed marker (base 10 of the logarithm is always used). Table pools over baseline strata. The time points are D35/C1/DD1 for cases and D35/C1/BD for non-cases.

- Differences in positive response rates (95% CI) between severe cases and non-cases at each time point (columns; 1 row only). 95% CI the Wilson-Score method without continuity correction (Newcombe, 1998). Table pools baseline strata. The time points are D35/C1/DD1 for cases and D35/C1/BD for non-cases. Repeat table where severe cases are swapped with Delta cases.
- Geometric mean ratio (95% CI) of quantitative marker between severe cases and non-cases at each time point (column; 1 row only). Table pools over baseline strata. The time points are D35/C1/DD1 for cases and D35/C1/BD for non-cases. Repeat table where severe cases are swapped with Delta cases.

Graphical Output

Set 1 plots: D35 Ab distributions by case/non-case

1. D35 antibody for the 2 $\log_{10}$ nAb ID50 titer markers (to D614G and to B16172). Within each panel there are side-by-side violin/boxplots for cases and non-cases.
2. Repeat 1 for the 2 $\log_{10}$ IgG anti-Spike markers (to D614 and to B16172)
3. Repeat 1 for transformed $\log_{10}$ marker values where the $\log_{10}$ marker values are transformed to percentiles in the population defined by ph1.D35_108==1, ewcdf($\log_{10}$ marker value, weight)($\log_{10}$ marker value), for each assay.

Set 2 plots: Longitudinal plots D35 to C1 to DD1/BD

1. For $\log_{10}$ nAb ID50 titer to D614G, plot 6 side-by-side violin/box plots, the first 3 for D35 non-cases, C1 non-cases, and BD non-cases, with lines connecting individual's data points, and the last 3 for D25 cases, C1 cases, DD1 cases, with lines connecting individual's data points. To the right of this plot, place the parallel results for $\log_{10}$ nAb ID50 titer to B16172.
2. Repeat 1. for $\log_{10}$ anti-Spike IgG (for D614 and B16172)

Set 3 plots: Correlation plots across markers at a given time point

1. For all 10 markers at D35, a pairs plot similar to those in Gilbert et al. (2022). Spearman rank correlation coefficients are included (including IPS weights).
2. Repeat 1. restricting to the 6 markers of focus as defined in Section 6.1.

Set 4 plots: Correlation plots for a given marker across time points

1. For each of the 10 markers, a figure with 2 panels, for the marker measured over the time points D35, C1, and BD for non-cases (column 1) and over D35, C1, and DD1 for cases (column 2). Spearman rank correlation coefficients are included.

6.2 Assessing Objective 1, 2 (≈peak Ab Correlates of Risk)

For the CoR Objectives 1 and 2, the planned analysis is similar to the originally published Stage 1 CoR analysis, implementing baseline-covariate marginalized Cox regression in the stratified random sample of two-dose vaccine recipients. The Cox regression modeling is done using study time to be consistent with what was done originally for PREVENT19.

Specifically, output for the analyzed markers listed in Section 6.1 is as follows.

1. (Obj. 1,2) Univariable Cox model results for each quantitative marker (hazard ratio, 95% CI, 2-sided p-value)
2. (Obj. 1,2) Univariable Cox model results for each tertilized marker (hazard ratios, 95% CIs, 2-sided p-values, Generalized Wald p-values)
3. (Obj. 1,2) Univariable Cox model marginalized marker-conditional mean cumulative incidence curves over time through to the last time point t_0, for Low, Medium, High tertile marker subgroups.
4. (Obj. 1,2) Univariable Cox model marginalized marker-conditional mean cumulative incidence curve over time through to the last time point t_0, with marker subgroups defined by the continuous value of the marker.

6.2.1 Covariates adjusted for in CoR and CoP analyses

We will adjust for Senior (age≥65) in the Delta COVID analyses and age in the severe COVID analyses. We do not adjust for the risk score included in the stage 1 analysis because of a lack of prediction power. Age is a known confounder for severe COVID. For Delta COVID, we choose a dichotomized version because of the complicated behavioral relationship between risk and age.

6.3 Assessing Objectives 3 (Exposure-Proximal Correlates of Risk)

We adopt a regression calibration (Prentice, 1982) based approach for estimating exposure-proximal immune correlates. Consider a hazard model for time to event of Delta COVID among vaccine recipients,

$$\lambda(s) = \lambda_0(s)\mathrm{e}\left(\beta_1 x(s-\tau) + \beta_2 W\right) I(\tau < s), \quad (1)$$

where s is calendar time, τ is peak time, $x(t)$ is true underlying immune response at time t post-peak, and W is the baseline covariate age group (≥ 65 vs < 65).

For each immune response (nab-ID50 D614G, nab-ID50 Delta, Anti-Spike D614, Anti-Spike Delta), we use linear effect model to model the log10 immune response trajectory over log(days post-peak + 35), with fixed effect for log(days post-peak + 35), age, sex and random intercept for individuals, adjusting for case-control sampling weights. Based on the LME model fit we estimate the expected value of immune response at any day post peak conditional on age, sex, and observed history of immune response measures and estimate the Cox model parameters by maximizing the partial likelihood based on induced hazard (Prentice, 1982). Hazard ratio curve given exposure-proximal

correlate x (i.e., HR per 10^x fold increase in biomarker level)

$$HR(x) = exp(\beta_1 x),$$

is then estimated based on β estimates. Nonparametric bootstrap with 500 samples will be used to construct 95% pointwise confidence interval for $HR(x)$.

7 Specifications for general issues faced for most analyses

7.1 Imputation of demographics variables for stratification and merging of sparse strata for weights computation

Two case strata are defined. First, a Delta COVID stratum is defined based on the indicator KnownOrImputedDeltaCOVIDInd_21Apr19to22Mar26; second, a severe COVID stratum is defined based on the indicator SevereCOVIDInd_21Apr19to22Mar26. The control strata depend upon the demo variables (age, at risk, minority) as in the Stage 1 correlates study.

8 Additional data analysis issues

8.1 Exclude participants reporting being HIV positive from the correlates analysis

Because the lentivirus-based pseudovirus neutralization assay uses an HIV backbone, the presence of anti-retroviral drugs in serum can give a false positive neutralization signal. For this reason, the original immune correlates analysis Gilbert et al. (2022) excluded participants who self-reported being HIV positive, because they would likely be taking anti-retroviral drugs. Consistent with the previous correlates analysis, this SAP also excludes participants who self-reported being HIV positive.

8.2 Missing lineages

Some endpoint cases will likely have missing lineage/spike sequence. If the COVID-19 endpoint diagnosis date is $\leq$ Dec 10, 2021 and $\geq$ 108 days post D35 visit, then the lineage will be hard-imputed to be Delta B16172.

8.3 End of followup and non-case definition

In the sampling plan, the non-case was defined with a right boundary of March 26, 2022 (the date Aug 22, 2022 was the actual data cut date, but Mar 26 was used for sampling). The sampling was done separately for cases and non-cases. On the other hand, our analysis plan re-defines the end of follow-up to be Dec 10, 2021. The Omicron cases that occurred after Dec 10, 2021 are right censored in the correlates analysis and hence are non-cases, but they are not represented in the ph2 samples. This results a small imperfection in the ph2 samples that we have.

8.4 Definition of severe COVID-19

Severe COVID-19 endpoints were defined as in the supplement of Dunkle et al. (2022), i.e. a first episode of RT-PCR positive COVID-19 with one or more of the following symptoms:

- Tachypnea ($\geq$ 30 breaths per minute at rest)
- Resting heart rate $\geq$ 125 beats per minute
- Oxygen saturation: $<$ 93 % on room air or partial pressure of oxygen in the alveolus/fraction of inspired oxygen $<$ 300 mmHg
- High flow oxygen therapy or non-invasive ventilation/non-invasive positive; non-invasive positive pressure ventilation (e.g., continuous positive airway pressure or bilevel positive airway pressure)
- Mechanical ventilation or extracorporeal membrane oxygenation
- One or more major organ system dysfunction or failure to be defined by diagnostic testing/clinical syndrome/interventions, including any of the following:
  - Acute respiratory failure, including acute respiratory distress syndrome
  - Acute renal failure
  - Acute hepatic failure
  - Acute right or left heart failure
  - Septic or cardiogenic shock (with shock defined as systolic blood pressure $<$ 90 mm Hg OR diastolic blood pressure $<$ 60 mm Hg
  - Acute stroke (ischemic or hemorrhagic)
  - Acute thrombotic event: acute myocardial infarction, deep vein thrombosis, pulmonary embolism
  - Requirement for: vasopressors, systemic corticosteroids, or hemodialysis
- Admission to an intensive care unit
- Death

Note that all participants with a single vital sign abnormality placing them in the severe category must also have met the criteria for mild COVID-19 (detailed in Dunkle et al. (2022)).

References

Dunkle, L.M., Kotloff, K.L., Gay, C.L., Áñez, G., Adelglass, J.M., Barrat Hernández, A.Q. et al (2022), "Efficacy and safety of NVX-CoV2373 in adults in the United States and Mexico," *New England Journal of Medicine*, 386, 531–543.

Fong, Y., Huang, Y., Benkeser, D., Carpp, L.N., Áñez, G., Woo, W. et al (2023), "Immune correlates analysis of the PREVENT-19 COVID-19 vaccine efficacy clinical trial," *Nature Communications*, 14, 331.

Gilbert, P.B., Montefiori, D.C., McDermott, A.B., Fong, Y., Benkeser, D., Deng, W. et al (2022), "Immune correlates analysis of the mRNA-1273 COVID-19 vaccine efficacy clinical trial," *Science*, 375, 43–50.

Newcombe, R. (1998), "Interval Estimation for the Difference Between Independent Proportions: Comparison of Eleven Methods," *Statistics in Medicine*, 17, 873–90.

Prentice, R.L. (1982), "Covariate measurement errors and parameter estimation in a failure time regression model," *Biometrika*, 69, 331–342.
